# Small-pore hydridic frameworks store densely packed hydrogen

**DOI:** 10.1038/s41557-024-01443-x

**Published:** 2024-02-06

**Authors:** Hyunchul Oh, Nikolay Tumanov, Voraksmy Ban, Xiao Li, Bo Richter, Matthew R. Hudson, Craig M. Brown, Gail N. Iles, Dirk Wallacher, Scott W. Jorgensen, Luke Daemen, Rafael Balderas-Xicohténcatl, Yongqiang Cheng, Anibal J. Ramirez-Cuesta, Michael Heere, Sergio Posada-Pérez, Geoffroy Hautier, Michael Hirscher, Torben R. Jensen, Yaroslav Filinchuk

**Affiliations:** 1 Department of Chemistry, Ulsan National Institute of Science and Technology, Ulsan, Republic of Korea; 2 Graduate School of Carbon Neutrality, Ulsan National Institute of Science and Technology, Ulsan, Republic of Korea; 3 Institute of Condensed Matter and Nanosciences, Université catholique de Louvain, Louvain-la-Neuve, Belgium; 4 Department of Chemistry and Interdisciplinary Nanoscience Center, Aarhus University, Aarhus, Denmark; 5 Center for Neutron Research, National Institute of Standards and Technology, Gaithersburg, MD USA; 6 Department of Crystallography, Helmholtz-Zentrum Berlin, Berlin, Germany; 7 School of Science, RMIT University, Melbourne, Victoria Australia; 8 Chemical and Environmental Sciences Lab, General Motors R&D Center, Warren, MI USA; 9 Hyrax intercontinental, Bloomfield, MI USA; 10 Spallation Neutron Source, Oak Ridge National Laboratory, Oak Ridge, TN USA; 11 Institute for Applied Materials (IAM), Karlsruhe Institute of Technology (KIT), 76344 Eggenstein-Leopoldshafen and Heinz Maier-Leibnitz Zentrum (MLZ), Technische Universität München, Garching, Germany; 12 Technische Universität Braunschweig, Institute of Internal Combustion Engines, Braunschweig, Germany; 13 Institut de Química Computacional i Catàlisi, Departament de Química, Universitat de Girona, Girona, Catalonia Spain; 14 Thayer School of Engineering, Dartmouth College, Hanover, NH USA; 15 Max Planck Institute for Intelligent Systems, Stuttgart, Germany; 16 Advanced Institute for Materials Research (WPI-AIMR), Tohoku University, Sendai, Japan

**Keywords:** Materials chemistry, Surface chemistry

## Abstract

Nanoporous materials have attracted great attention for gas storage, but achieving high volumetric storage capacity remains a challenge. Here, by using neutron powder diffraction, volumetric gas adsorption, inelastic neutron scattering and first-principles calculations, we investigate a magnesium borohydride framework that has small pores and a partially negatively charged non-flat interior for hydrogen and nitrogen uptake. Hydrogen and nitrogen occupy distinctly different adsorption sites in the pores, with very different limiting capacities of 2.33 H_2_ and 0.66 N_2_ per Mg(BH_4_)_2_. Molecular hydrogen is packed extremely densely, with about twice the density of liquid hydrogen (144 g H_2_ per litre of pore volume). We found a penta-dihydrogen cluster where H_2_ molecules in one position have rotational freedom, whereas H_2_ molecules in another position have a well-defined orientation and a directional interaction with the framework. This study reveals that densely packed hydrogen can be stabilized in small-pore materials at ambient pressures.

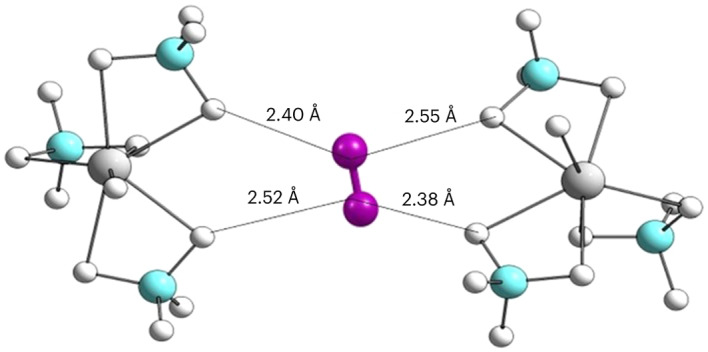

## Abstract (web summary)

Although hydrogen gas could serve as a promising future fuel, its high-capacity storage is a challenge. Now, a nanoporous magnesium borohydride framework is shown to store hydrogen as densely packed penta-dihydrogen clusters having well-defined orientations and directional interactions with the framework.

## Main

The widespread use of hydrogen as a fuel in personal and public transport vehicles is limited by the great challenge of hydrogen storage. Current technology mainly focuses on molecular hydrogen storage in either gaseous (for example, $p_{\rm{H}_2} = 700\, {\rm{bar}}$) or liquid (at cryogenic temperatures) phases^1,2^. Unfortunately, there are limits to the volumetric and gravimetric storage density that current technologies can provide. Hydrogen, as a molecule, can physically adsorb in a porous material by weak van der Waals interactions (London dispersion forces) in a process called physisorption^3,4^. Highly porous materials offer high gravimetric hydrogen uptake, but the volumetric storage capacity still needs improvement^5^.

A nanoporous cubic magnesium borohydride, γ-Mg(BH_4_)_2_ (density of *ρ* = 0.550 g cm^−3^, space group *Ia*-3*d*) has a net of linear channels, with 33% free pore volume^6^. The pore diameter is ∼9 Å, and the smallest aperture measures ∼5.8 Å, enabling this compound to adsorb small molecules such as dichloromethane, nitrogen or hydrogen. This porous hydride has a non-flat, partially negatively charged inner surface, where the hydridic H^δ−^ atoms are exposed into the pores. Using synchrotron radiation X-ray powder diffraction (SR-XRPD), nitrogen molecules were located close to the centre of the pore, corresponding to one molecule per pore and thus reaching the limiting composition γ-Mg(BH_4_)_2_·2/3N_2_. The hydrogen molecular positions could not be determined independently in previous investigations due to hydrogen’s small X-ray scattering length and were assumed to occupy the same position as N_2_ (ref. ^6^). In this Article we report hydrogen with weak van der Waals interactions and an electrostatic interaction with the hydridic framework, forming dense penta-dihydrogen clusters.

## Results and discussion

### Neutron powder diffraction

**Fig. 1:**
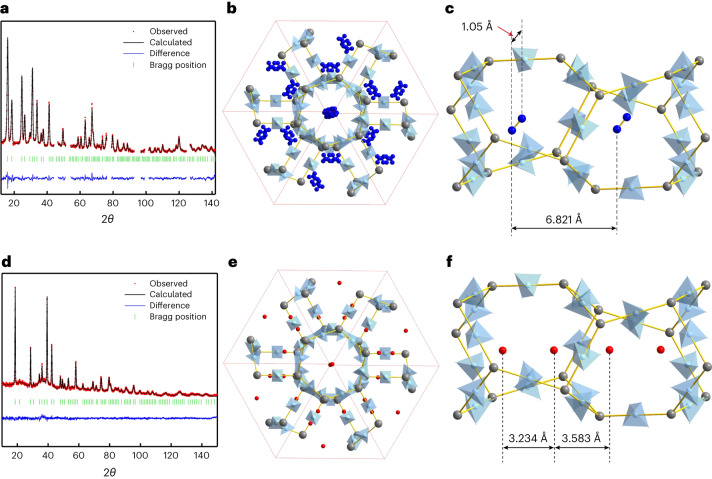
NPD profiles for nitrogen and deuterium-loaded γ-Mg(^11^BD_4_)_2_, along with projections of the crystal structures. **a**–**c**, The nitrogen-loaded γ-Mg(^11^BD_4_)_2_·2/3N_2_ sample at 100 K and $p_{\rm{N}_{2}} = 3\, {\rm{bar}}$ (Helmholtz-Zentrum Berlin data, *λ* = 1.7982 Å). **a**, Rietveld refinement profile. The angular ranges containing contributions from the Al sample holder were excluded from the refinement. **b**, The superposition of nitrogen adsorption sites in the crystal structure of nanoporous γ-Mg(BH_4_)_2_, viewed along the [111] direction. Mg atoms are shown as grey spheres, BH_4_ groups as blue tetrahedra, disordered N_2_ molecules as blue spheres, and the unit cell is defined by red lines. **c**, The porous channel running along the cubic diagonal displaying an N_2_ molecule in the centre of the pore, viewed along the [1–11] direction. The distance between two neighbouring N_2_ molecules is 6.821 Å. **d**–**f**, The deuterium-loaded γ-Mg(^11^BD_4_)_2_ sample at 10 K (NIST data, *λ* = 2.079 Å). **d**, Rietveld refinement profile, using the new structural model that allows for the double hydrogen capacity, γ-Mg(^11^BD_4_)∙1.33D_2_. **e**, Superposition of deuterium adsorption sites (denoted D11) viewed along the [111] direction, D_2_-centroids are shown as red spheres. **f**, The porous channel running along the cubic diagonal, displaying two D_2_ per pore, taking positions close to the apertures, viewed along the [1–11] direction. The distances of intra-/inter-pore D_2_ are 3.234 Å and 3.583 Å, respectively. Source data

Using neutron powder diffraction (NPD), we accurately determined the position of the hydrogen atoms in the framework structure, as well as the adsorption sites of the molecules. Isotopically enriched γ-Mg(^11^BD_4_)_2_ and deuterium (D_2_) gas make NPD the ideal diffraction technique to locate hydrogen atoms by using the large neutron coherent cross-section of deuterium. High-resolution, high-flux neutron powder diffractometers are suitable for fast, in situ studies involving rapidly changing gas–solid systems, particularly when one of the gases is deuterium^7^. For the data analysis, we initially performed a Rietveld refinement of the NPD data to confirm the ‘empty’ framework structure of γ-Mg(BD_4_)_2_ (Supplementary Fig. 1 and Supplementary Table 1) and the SR-XRPD-based structural model γ-Mg(BH_4_)_2_·2/3N_2_, which corresponds to full occupation of the pore with one nitrogen molecule, positionally disordered around the pore’s centre (Fig. 1a, Supplementary Fig. 2 and Supplementary Table 2).

We discovered that D_2_ is not placed in the centre position of the pore, as is the case for N_2_ (Fig. 1b,c; a comparison of the Rietveld refinement profiles for the two localization models is provided in Supplementary Fig. 3). Instead, the D_2_ molecules are located close to the aperture of the pore and the two D_2_ molecules in the pore are symmetry-equivalent and described with a single crystallographic site, denoted D11 (Fig. 1e,f).

The limiting composition of this structure corresponds to 4/3 D_2_ per Mg(BD_4_)_2_, that is, Mg(BD_4_)_2_·1.33D_2_, and this is easily saturated at $p_{\rm{D}_{2}} = 27\,{\rm{mbar}}$ and *T* = 25 K. In the structural model, deuterium is represented by a quantum rotor that is often referred to as a ‘super atom’^8^, with a centroid of a sphere of nuclear density around the centre of mass of the D_2_ molecule; that is, the molecule has full rotational freedom around the centre of mass. It is described with a single positional parameter, one occupancy factor (which accounts for two atoms in a molecule, that is, an occupancy of 2 indicates one molecule) and one atomic displacement parameter. The D11 site is located on the three-fold axis, forming a 1D chain along the [111] direction of the cubic unit cell (Fig. 1e). The guest–guest contacts between the centres of the deuterium molecules within the same pore and between different pores are 3.234(5) Å and 3.583(5) Å, respectively (Fig. 1f). These distances are substantially shorter than the distances between H_2_ centroids in solid hydrogen (∼3.8 Å)^9^, allowing a pore in γ-Mg(^11^BD_4_)_2_ to accommodate two D_2_ molecules closer to the apertures, whereas only one larger N_2_ occupies the centre of the pore. Note that the kinetic diameters of D_2_/H_2_ and N_2_ are similar, with *d*(H_2_/D_2_) = 2.89 Å and *d*(N_2_) = 3.64 Å (Fig. 1c).

The host–guest interaction is defined by the contacts involving the framework’s deuterium atoms, that is, the partially negatively charged hydrogen atoms of the borohydride groups, at 2.992(6) Å away from the D_2_ centroid, exhibiting strong H^δ−^···H_2_ adsorption at near-ambient pressure. Metal–organic frameworks (MOFs) and hydrogen hydrates^10^ do not contain hydridic hydrogen, so they do not exhibit similar molecular arrangements. The other example, metal hydride clathrates, do not contain original hydrogen molecules due to the transfer of electrons from the metal atoms to the hydrogen atoms, and are stable only at high pressures^11^. The affinity of H_2_ and H^δ−^ is also illustrated by the fact that deuterium molecules remain close to the aperture and not to the centre of the pore at low and intermediate hydrogen loadings. This is illustrated by γ-Mg(BD_4_)_2_·0.35D_2_ and γ-Mg(BD_4_)_2_·1.05D_2_, respectively, at $p_{\rm{D}_{2}} = 0.21$ and 0.43 mbar and *T* = 25 K (Supplementary Table 4 and Supplementary Fig. 4 summarize the results of the neutron diffraction study). Thus, the D_2_ molecule does not move to the centre of the pore, where more space is available, even at low loadings. We can conclude that the specific H^δ−^···H_2_ interaction leads to well-defined H_2_ adsorption at the D11 site, both at low and high loadings.

Re-evaluating the previously published H_2_ adsorption in γ-Mg(BH_4_)_2_ isobars extracted from SR-XRPD data^6^ using the ‘D11 model’ provides an improved fit to the observed diffraction data collected at hydrogen pressures of 3.34, 33.7 and 105 bar and temperatures down to 80 K. The extracted isobars have more regular shapes and also give the maximum capacity of 4/3 H_2_ per formula unit, in good agreement with the neutron diffraction data. Supplementary Section 2 provides comparisons between the N_2_ model and the D11 model.

Structure revision using NPD and SR-XRPD data firmly shows that the physisorbed hydrogen density in γ-Mg(BH_4_)_2_·1.33H_2_ reaches ∼5.0 mass fraction (wt%). Considering the 0.60 cm^3^ g^−1^ total pore volume in γ-Mg(BH_4_)_2_, the observed density of hydrogen molecules in the pores, 82 g H_2_ l^−1^, is higher than the density of liquid hydrogen at 21 K and 1 bar (71 g H_2_ l^−1^) and close to the density of solid hydrogen (86 g H_2_ l^−1^).

**Fig. 2:**
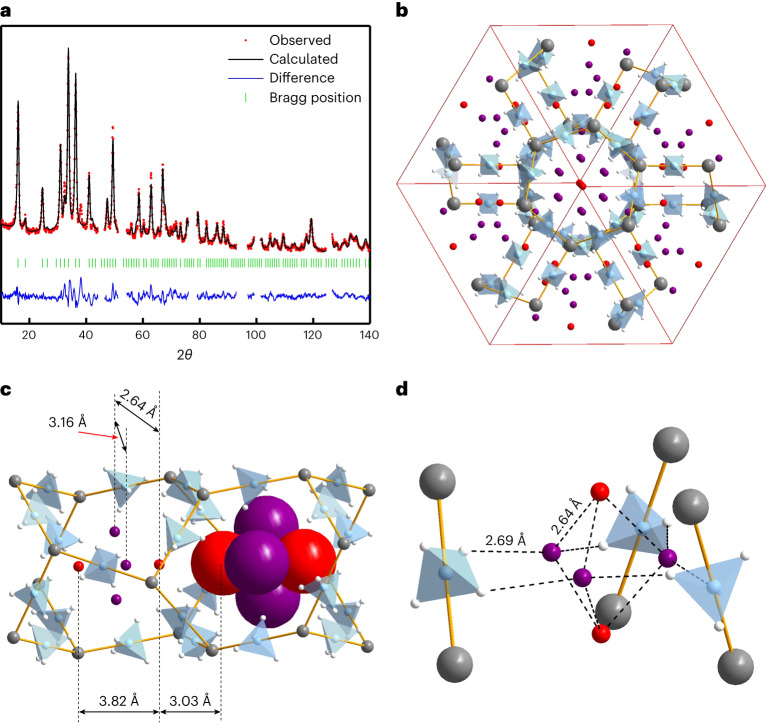
Deuterium-loaded γ-Mg(^11^BD_4_)_2_ at 25 K and 203 mbar. **a**, Rietveld refinement NPD data profile, at *λ* = 1.799 Å. Angular ranges containing contributions from the Al sample holder were excluded. **b**, Crystal structure containing two D_2_ sites with the limiting composition of γ-Mg(BH_4_)_2_∙2.33D_2_. Mg atoms are shown as grey spheres and BH_4_ groups as blue tetrahedra. The unit cell is defined by red lines, the D11 positions of the D_2_ centroids are shown as red spheres and the D22 positions of the D_2_ centroids as purple spheres. **c**, The porous channel running along the cubic diagonal, displaying the two D_2_ sites. The spheres in the right pore are shown with the van der Waals radius of the hydrogen molecule in the solid state at ambient pressure (1.52 Å)^26^. **d**, The environment of five H_2_ molecules occupying the same pore, highlighting short B−H^δ−^···H_2_ and H_2_···H_2_ contacts. Source data

At higher deuterium pressures, at 25 K, we observed a second step of gas uptake. Analysis of the NPD data reveals that a second D_2_ site, denoted D22, starts to become occupied (Fig. 2b), reaching saturation at $p_{\rm{D}_{2}} = 205\,{\rm{mbar}}$ and *T* = 25 K. D22 is not positioned on the three-fold axis, running through the centre of the channel, thus creating a short (self-excluding) D22…D22 distance of 1.94 Å between the two bypassing channels. As a consequence, the limiting occupancy of the D22 site is 50%. Careful re-analysis of the diffraction data measured at $p_{\rm{D}_{2}} = 27\,{\rm{mbar}}$ and *T* = 25 K indicates that the D22 site is 14% filled at this pressure, while at 203 mbar it becomes fully occupied (50%), reaching the limiting composition of γ−Mg(BD_4_)_2_·2.33D_2_, that is, simultaneous occupation of both D11 and D22 positions. Rietveld refinement profiles are provided in Fig. 2a and crystallographic data in Supplementary Table 6. Extra weak diffraction peaks appear in the NPD patterns of γ-Mg(BD_4_)_2_·2.33D_2_, showing that the body-centred structure becomes primitive, most probably due to an order of D22 sites in a lower-symmetry space group (possibly *Pa*−3).

The crystallographic data thus reveal the composition γ-Mg(BH_4_)_2_·2.33H_2_ at saturation, corresponding to 8.0 mass fraction (wt%) physisorbed hydrogen, and taking into account the hydrogen of the framework, this translates to a total gravimetric and volumetric hydrogen content of 21.7 mass fraction (wt%) and 129 g l^−1^, respectively. The physisorbed hydrogen (2.33 H_2_) is packed extremely densely in the pores, at ∼144 g H_2_ per litre of pore volume, which is twice the density of liquid hydrogen. This high density stems from the short contact distances between the hydrogen atoms of the BH_4_ group and the centres of the H_2_ molecules (the shortest is D1…D22 of 2.69(1) Å) and within the trigonal bipyramidal cluster composed of five H_2_ molecules shown in Fig. 2c (the shortest is D11…D22 of 2.64(1) Å). The latter compares well with the 2.66 Å H_2_···H_2_ distance in phase I of solid H_2_ at 5.4 GPa^12^.

### Inelastic neutron scattering

Spectroscopic characterization was performed using inelastic neutron scattering (INS) measurements of γ-Mg(BD_4_)_2_ on the empty framework and with physisorbed H_2_ using the high-resolution spectrometer VISION at the high-flux neutron spallation source at Oak Ridge National Laboratory (ORNL)^13^. INS is a technique that is particularly useful for identifying the rotational excitations of the hydrogen molecule (H_2_)^9,14^.

**Fig. 3:**
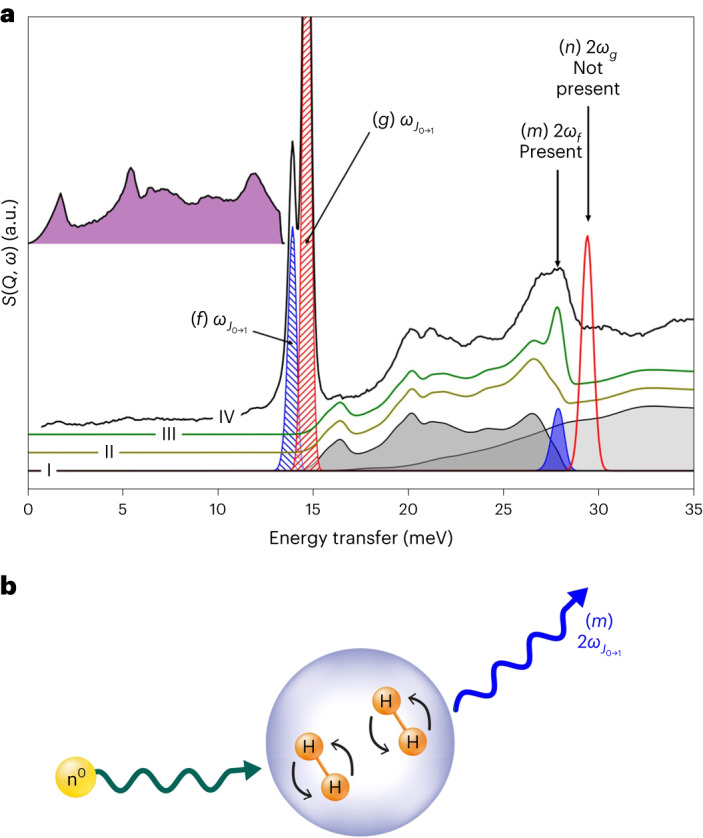
Inelastic neutron scattering spectra of deuterium-loaded γ-Mg(BD_4_)_2_. **a**, INS spectra of Mg(BD_4_)_2_ loaded with 4 mmol of *p*-H_2_ (black). The vibrational DoS (purple upper area) is convoluted with the rotor transitions that are shown as red and blue hashed areas (peaks *f* and *g*) (Supplementary Section 5 presents the full peak assignment). The resulting convolution is shown as the greyed-out areas in trace I to obtain the overtones and combinations. The sum of the overtones is shown in trace II. Trace III includes the contribution of the concurrent excitation of *J*_0→1_ on two neighbouring hydrogen molecules. **b**, Scheme of SQRE. A single neutron interacts simultaneously with two neighbouring H_2_ molecules. The resulting neutron transfers the energy required for two rotational transitions ${2{\omega }_{{J}_{0\to 1}}}$ (27.8 meV). Only the SQRE of the rotational transition at 13.9 meV shows in the spectrum. All INS spectra presented in this Article were measured at 5 K. Source data

Figure 3a shows the INS spectrum of *para*-hydrogen (*p*-H_2_) adsorbed on γ-Mg(BD_4_)_2_, corresponding to Mg(BD_4_)_2_∙1.46H_2_, with the background signal associated with the sample holder and the empty framework subtracted. With a correction for the ∼30% amorphous non-porous magnesium borohydride, the effective loading of the crystalline porous fraction is 2.06 H_2_ per Mg atom, indicating full loading of the D11 site and substantial loading of the D22 site. The spectrum contains two rotational peaks at 13.9 meV and 14.6 meV. The higher energy transition is consistent with a free hydrogen rotor (Fig. 3a, red hashed area), similar to solid hydrogen and hydrogen under confinement^15^. Accordingly, it can be assigned to the D11 site. The second rotational peak with lower intensity and lower energy is consistent with a perturbed rotor (blue hashed area)^9,16,17^, and can be attributed to the D22 site.

The total INS spectrum can be constructed using the contributions from the density of states (DoS) of the rattling modes of the hydrogen molecule, obtained from the spectra of normal hydrogen (*n*-H_2_, that is, a mixture of *ortho* (*o*-H_2_) and *para* (*p*-H_2_) hydrogen), as presented in refs. ^18,19^. The convolution of the rotational transitions with the DoS yields the total spectrum (Supplementary Section 5.5). As seen in Fig. 3, trace II, the reconstructed spectrum lacks intensity at 27.8 meV. This peak at 27.8 meV has an energy that matches twice the transition ${J}_{0\to 1}$ of the rotational peak (blue hashed area). However, there is no rotational transition of *p*-H_2_ that can account for this intensity (Supplementary Table 8). A similar effect can be seen when measuring hydrogen under high pressure (Supplementary Section 5.5 and Supplementary Fig. 21). The small peak present in solid hydrogen at 29.1 meV at 100 bar increases manyfold at 2,500 bar. This peak is a consequence of a simultaneous quantum rotational excitation (SQRE) of two distinct *p*-H_2_ molecules. The pressure makes these molecules interact with each other, so a single neutron scattering interaction with one of the molecules induces a simultaneous transition in the other molecule as well (Supplementary Section 5.3). The neutron transfers the energy required for both excitations, ${\omega }_{\rm{eff}}={2{\omega }_{{J}_{0\to 1}}}$. Only the low-energy rotational transition (Fig. 3, peak *f*) exhibits this behaviour. The peak at 14.6 meV does not show the equivalent transition at 29.2 meV, so these transitions correspond to two different adsorption sites.

By looking at the effect of pressure on highly enriched *p*-H_2_ and *n*-H_2_ (Supplementary Section 5.5) on the phonon spectrum of hydrogen, we can calibrate a ‘representative density’ required for the effect of pressure to be observable. The corresponding pressure of 2,915 bar is consistent with a hydrogen density of 132 g l^−1^ and an estimated average H_2_–H_2_ intermolecular distance of 3.29 Å (Supplementary Section 5.6).

Supplementary Fig. 18 shows the effect of temperature on the INS spectra for the composition Mg(BD_4_)_2_∙1.46H_2_. Observing the evolution of the peaks with temperature, we see a different behaviour corresponding to two different sites for hydrogen molecules. The peaks at 13.9 meV and 27.8 meV, assigned to D22, disappear simultaneously when the temperature reaches 95 K. The peak at 14.7 meV, attributed to D11, is still observed up to 185 K. Thus, INS enables an independent determination of the high H_2_ density inside the pore, and the rotational spectrum confirms the existence of two distinct sites for the hydrogen molecules, fully in agreement with the neutron diffraction data.

### Volumetric hydrogen adsorption measurements

**Fig. 4:**
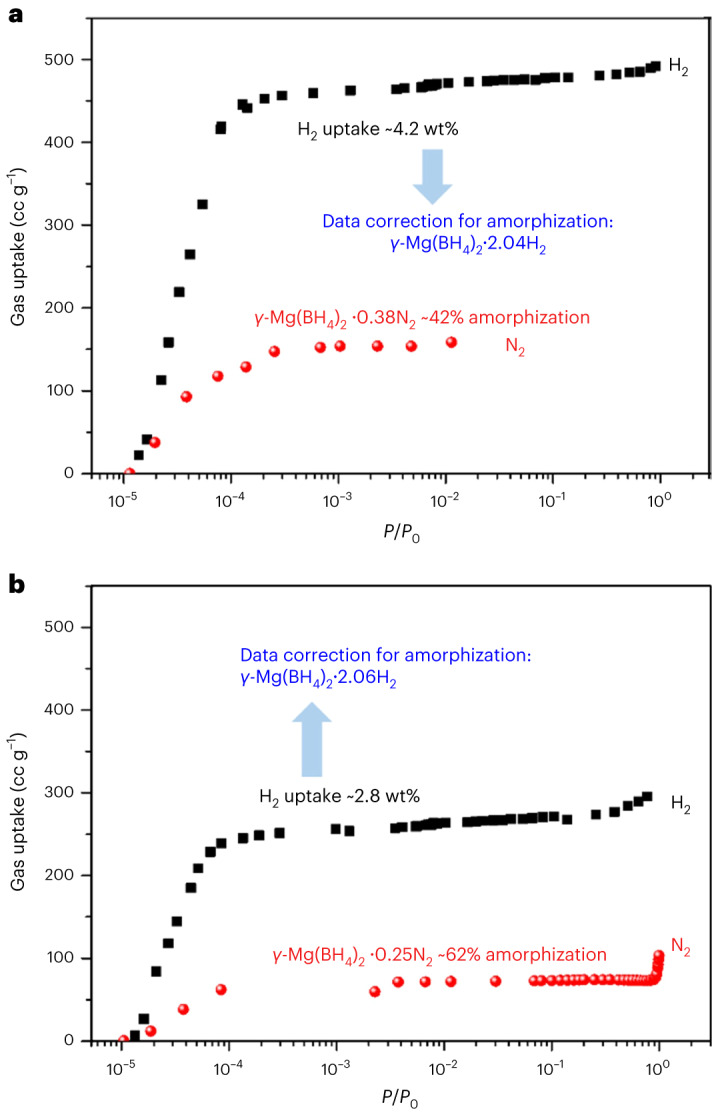
Adsorption isotherms of N_2_ at 77 K and H_2_ at 20 K, respectively. **a**, The first batch sample with short-term storage, exhibiting 42% amorphization. Data correction for the amount of the amorphous phase results in γ-Mg(BH_4_)_2_∙2.04H_2_. **b**, The second batch sample with long-term storage, exhibiting 62% amorphization. Data correction for the amount of the amorphous phase results in γ-Mg(BH_4_)_2_∙2.06H_2_. Source data

To further verify the presence of two hydrogen adsorption sites and to characterize their thermodynamics with a macroscopic measurement, we used a volumetric Sievert’s apparatus, based on the measurement of the pressure variation with time for well-calibrated volumes of gas equilibrated with the sample. Unlike diffraction methods, high-resolution adsorption measurements are not only probing the crystalline phase γ-Mg(BH_4_)_2_; an amorphous fraction in all samples also needs to be considered. We quantified the crystalline fraction of γ-Mg(BH_4_)_2_ by attributing the amount of adsorbed nitrogen, as determined from a volumetric isotherm, only to the adsorption in the crystalline phase (see Supplementary Section 4 for details and Fig. 4 for two examples). Notably, the volumetric H_2_ uptake, corrected for the presence of the amorphous fraction, consistently shows γ-Mg(BH_4_)_2_∙∼2.05H_2_, very close to the limiting composition obtained from the crystallographic data (γ-Mg(BH_4_)_2_·2.33H_2_).

We observe about three times larger adsorption of H_2_ as compared to N_2_ for each sample. This is possibly a very rare example of two similar probe molecules, H_2_ and N_2_, with very different interactions with the topology of the surface, as pointed out previously^20^. Similar to the gas uptake in Fig. 4, the Brunauer–Emmett–Teller (BET) specific surface area of γ-Mg(BH_4_)_2_ determined from the N_2_ isotherm at 77 K is only *S*_BET_(N_2_) = 610 m^2^ g^−1^, whereas that calculated from the H_2_ isotherm at 20 K is *S*_BET_(H_2_) = 1,787 m^2^ g^−1^ or 1,577 m^2^ g^−1^, using a cross-sectional area of the hydrogen molecule based on the liquid or solid density, respectively (Fig. 4a). Furthermore, the total specific pore volume (SPV, max. uptake per sample mass/liquid gas density) of γ-Mg(BH_4_)_2_ determined from the N_2_ isotherm at 77 K is only 0.12 ml g^−1^, whereas the SPV calculated from the H_2_ isotherm at 20 K is 0.51 ml g^−1^. The crystallographic investigation revealed that the pore size and pore aperture in γ-Mg(BH_4_)_2_ allow both H_2_ and N_2_ to enter and reach saturation. Thus, this study provides unambiguous evidence for the strong correlation between the measured specific surface area and the probe molecule owing to different interactions with a non-flat surface topology^20^ rather than restricted access to the porous area of the structure mediated by pore aperture size. Please note that, even though the total uptake is reduced by aging due to the sample’s amorphization (Fig. 4b), the probe molecule effect consistently exhibits a similar tendency (for the second batch, *S*_BET_(N_2_) = 310 m^2^ g^−1^ and *S*_BET_(H_2_) = 1,110 m^2^ g^−1^ or 975 m^2^ g^−1^, cross-sectional area based on liquid or solid H_2_, respectively, and SPV(N_2_) = 0.08 ml g^−1^ and SPV(H_2_) = 0.41 ml g^−1^; Fig. 4b). Importantly, the ratios between the total uptakes of H_2_ and N_2_, as well as of the respective BET areas and SPVs, are very close to the ratio of the H_2_ and N_2_ limiting uptakes seen by diffraction, namely 2.33/0.66 = 3.5. Moreover, diffraction studies show that the guest–host and guest–guest interactions are responsible for the discrepancy in the surface area analysis, rather than the pores being too small for a particular molecule, as would be the case in size exclusion.

**Fig. 5:**
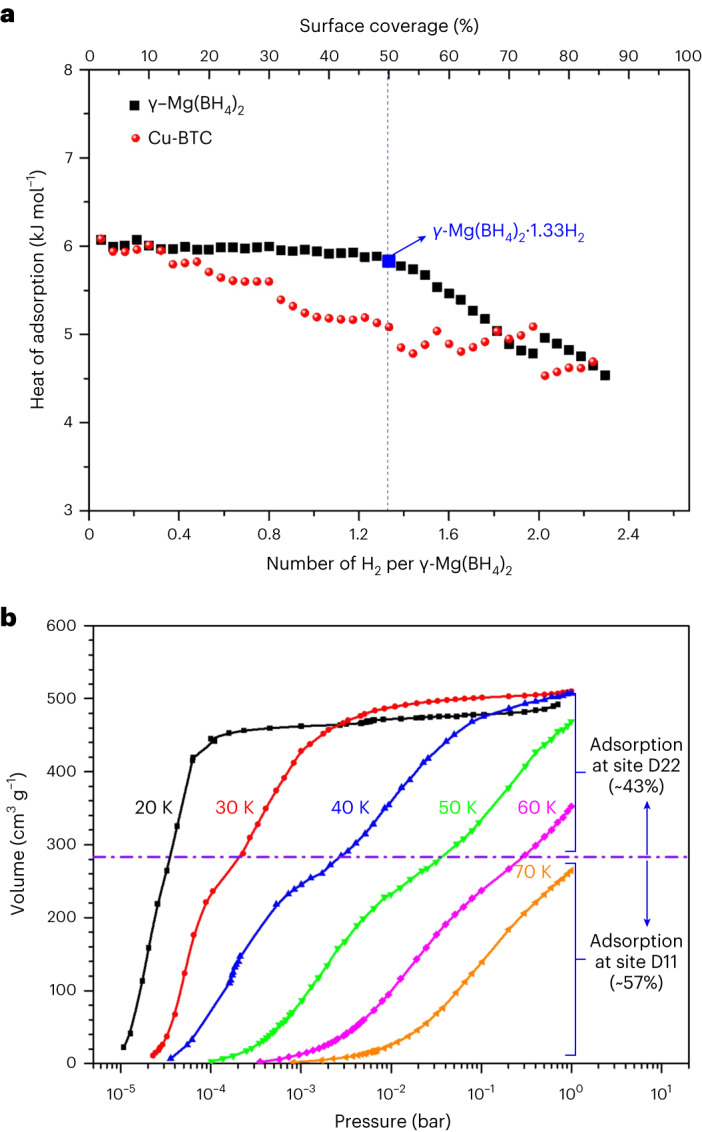
Characterization of hydrogen adsorption in γ-Mg(BH_4_)_2_ by volumetric methods. **a**, Comparison of the isosteric heat of hydrogen adsorption for γ-Mg(BH_4_)_2_ and a MOF compound (Cu–BTC, which has a similar pore diameter and H_2_ uptake) as a function of surface coverage, recalculated to the H_2_ loading per formula unit. Black squares indicate γ-Mg(BH_4_)_2_ and red circles Cu–BTC^21^. **b**, High-resolution low-pressure H_2_ adsorption isotherms for γ-Mg(BH_4_)_2_ at various temperatures between 20 and 70 K. With increasing temperature, two clear steps in the adsorption isotherms become visible. Source data

Temperature-dependent gas adsorption experiments also provide access to the isosteric heats of adsorption, which characterize the strength of host–guest and guest–guest interactions. The isosteric heat of hydrogen adsorption as a function of the surface coverage is shown in Fig. 5a for γ-Mg(BH_4_)_2_ and a copper benzene-1,3,5-tricarboxylate (Cu–BTC) MOF possessing a similar pore diameter and H_2_ uptake. The hydrogen adsorption enthalpy is almost constant at 6.1 kJ mol^−1^ up to ∼50% surface coverage and then decreases to 4.5 kJ mol^−1^, whereas that of similar MOFs, such as Cu–BTC, typically decreases with coverage^21^. The decrease in the isosteric heat of adsorption at loading above 1.33 H_2_ per Mg can be explained by the host–guest and guest–guest repulsion involving atoms in the D22 site, which also coincides with the unit cell volume expansion by 0.67% (whereas no expansion is observed upon filling the D11 site). Further evidence is also observed in the high-resolution low-pressure H_2_ adsorption isotherm, as shown in Fig. 5b. In the logarithmic pressure scale, the isotherm exhibits a two-step behaviour, which indicates sequential adsorption at two major sites with different retention energy. Assuming monolayer adsorption in these sites, the maximum hydrogen content expected in the first adsorption site (D11) is ∼57% of the total uptake. Thus, the remaining ∼43% is assigned to be the second site (D22), which also agrees very well with the NPD results.

### Periodic density functional simulations

**Fig. 6:**
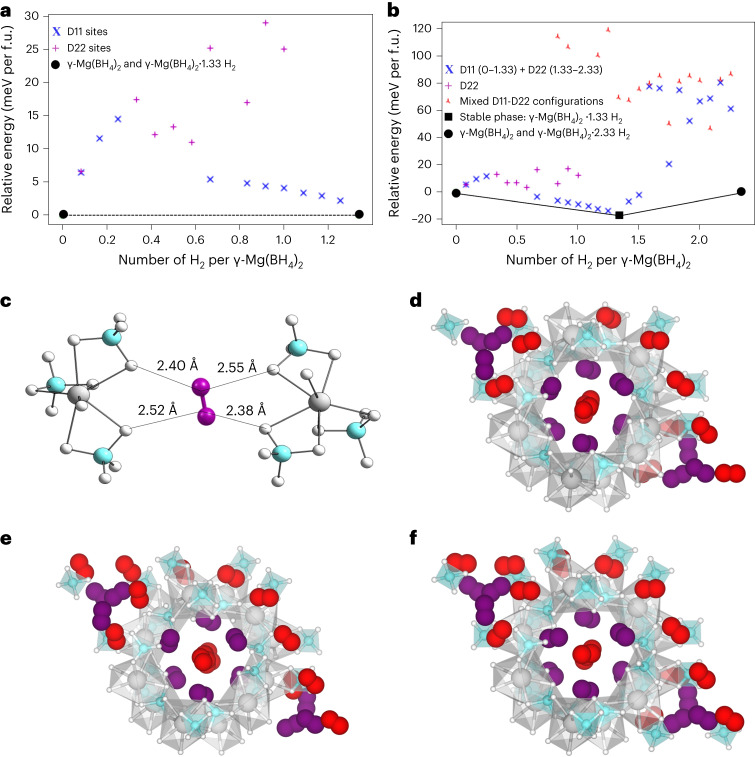
Thermodynamic convex hull analysis of H_2_ adsorption on γ-Mg(BH_4_)_2_ and schematic representation of (B−H^δ−^)_2_···H−H···(H^δ−^−B)_2_ connectivity on γ-Mg(BH_4_)_2_ and the H_2_ distribution. **a**, Convex hull analysis for a concentration of H_2_ from zero to 1.33 H_2_ per γ-Mg(BH_4_)_2_. The DFT energies refer to the empty framework γ-Mg(BH_4_)_2_ and γ-Mg(BH_4_)_2_·1.33H_2_. Blue and purple crosses represent configurations of γ-Mg(BH_4_)_2_·*x*H_2_ with adsorption on the D11 and D22 sites, respectively. The *y* axis represents the energy per γ-Mg(BH_4_)_2_ formula unit (f.u.). **b**, Convex hull analysis for a concentration of H_2_ from zero to 2.33 H_2_ per γ-Mg(BH_4_)_2_. The DFT energies are referred to the empty framework γ-Mg(BH_4_)_2_ and γ-Mg(BH_4_)_2_·2.33H_2_. The γ-Mg(BH_4_)_2_·1.33H_2_ structure (full D11 site adsorption) is stable and on the convex hull. Blue crosses show the relative energy per γ-Mg(BH_4_)_2_ of the full D11 loading followed by D22, purple crosses show isolated adsorption on D22, and red crosses the mixed configurations where D11 and D22 sites are partially occupied. **c**, Adsorption mode of H_2_ on the D22 site in fully loaded γ-Mg(BH_4_)_2_·2.33H_2_. The hydrogen atoms of the hydrogen molecules are connected to H^δ−^ with shorter distances than the D11 sites. **d**–**f**, Sketches of energetically equivalent γ-Mg(BH_4_)_2_·2.33H_2_. Red and purple H_2_ represent D11 and D22 sites, respectively. Note that H_2_ directionality on the D22 sites does not vary. Source data

H_2_ adsorption on γ-Mg(BH_4_)_2_ was further explored using periodic density functional simulations (Supplementary Section 6). The first insight from theory relates to the binding energy of H_2_ in the hydride. The computed reaction energy for one H_2_ per unit cell that contains 12 Mg atoms (γ-Mg(BH_4_)_2_ + 1/12 H_2_ → γ-Mg(BH_4_)_2_∙1/12 H_2_) is around −3 kJ mol^−1^ when binding on D11 and −2.9 kJ mol^−1^ when binding on D22. However, as the loading of H_2_ increases, the preference for D11 versus D22 increases (Supplementary Fig. 25). The maximum value of binding energy was found for fully loaded D11 sites, γ-Mg(BH_4_)_2_·1.33H_2_, with an adsorption energy of −10.5 kJ mol^−1^ for γ-Mg(BH_4_)_2_ + 1.33 H_2_ → γ-Mg(BH_4_)_2_∙1.33H_2_. The large binding energy obtained for the full occupation of D11 sites hints at a preference for this configuration. Figure 6a, which plots the energy of all the γ-Mg(BH_4_)_2_∙xH_2_ configurations (with H_2_ on D11 or D22) versus the empty framework γ-Mg(BH_4_)_2_ and γ-Mg(BH_4_)_2_∙1.33H_2_ with a fully occupied D11 site, confirms this view. This stability analysis is often referred to as a convex hull construction (Supplementary Information). At low temperatures (our computations are at 0 K), the H_2_-loaded γ-Mg(BH_4_)_2_ behaves as a two-phase system (γ-Mg(BH_4_)_2_ and γ-Mg(BH_4_)_2_·1.33H_2_) and not a solid solution. During H_2_ uptake, one can find regions without adsorbed H_2_ molecules and other regions of the material with all the D11 sites fully occupied. This thermodynamic analysis agrees with the low-temperature hydrogen adsorption data (Fig. 5b), where a steep uptake of H_2_ is observed at 20 K. From a structural point of view, the adsorption of H_2_ on all available D11 sites implies slightly larger guest–guest and host–guest distances than for D22 and thus allows hydrogen molecules to have full rotational freedom. Focusing on γ-Mg(BH_4_)_2_·1.33H_2_, the electron localization function plots, illustrated in Supplementary Fig. 26, show that the host–guest interaction of the D11 position is purely a van der Waals interaction without charge transfer. The relative computed energies of different H_2_ orientations at full occupancy of the D11 site are degenerate, confirming that H_2_ behaves as a free rotor (Supplementary Fig. 27). In other words, the host–guest interaction is not affected by the orientation of the H_2_ molecules placed in D11. The guest–guest and host–guest distances of the D11 site do not vary, thus showing the full dynamic disorder of H_2_, in agreement with the experiments.

After the full uptake of hydrogen on the D11 site, more H_2_ can be adsorbed on D22. Supplementary Fig. 28 shows the binding energy for filling the D22 sites. In agreement with the preference for fully loading the D11 sites, we observed that the filling of the D22 sites after fully loading the D11 sites is preferred over any mixed configuration where D11 and D22 sites are partially occupied. A convex hull analysis for the entire range of H_2_ loading (up to 2.33 H_2_) shows (Fig. 6b) that after occupying D11 fully, the system prefers energetically to occupy the D22 site as well as forming Mg(BH_4_)_2_·2.33H_2_. Our thermodynamic analysis reveals a two-phase system, γ-Mg(BH_4_)_2_·1.33H_2_–γ-Mg(BH_4_)_2_·2.33H_2_, where partial filling the D11 and D22 sites is always less stable (see Supplementary Section 6 for a detailed explanation). This is due to formation of the energetically more favoured trigonal bipyramidal penta-dihydrogen clusters, in full accord with the diffraction experiments (Fig. 2c and Supplementary Fig. 29). From the point of view of energetics, we observed that completing the penta-H_2_ cluster by filling the three D22 sites is favoured by 1 kJ mol^−1^ per H_2_ molecule compared to the partially filled D22 sites and incomplete penta-H_2_ clusters. With respect to structural concerns, hydrogen at D22 is slightly closer to the hydridic framework than D11, which clearly introduces directionality in the interaction. Hydrogen molecules placed on D22 sites are in a well-defined orientation relative to the framework. This creates an interaction between the neutral molecule and the partly negatively charged framework, B−H^δ−^···H_2_, characterized as an electrostatic interaction (Fig. 6c and Supplementary Fig. 30). In fact, a hexa-hydrogen bond, (B−H^δ−^)_2_···H−H···(H^δ−^−B)_2_, is discovered with a strength of 8.5 kJ mol^−1^ per H_2_ molecule. The well-defined directionality of H_2_ molecules placed in D22 consistently occurs in all the configurations having identical energy independently of D11 orientation (Fig. 6b–d), which agrees with the experimental INS spectra where one peak is assigned to a perturbed rotor. Indeed, single-point calculations of H_2_ on D22 reveal that the exact directionality has a notable impact on the relative total energy (Supplementary Fig. 31). Thus, although hydrogen molecules on the D11 position have full rotational freedom, H_2_ on the D22 position has a very well-defined directional interaction with the framework.

Nanoporous γ-Mg(BH_4_)_2_ is a hydridic framework with partially negatively charged hydrogen atoms forming the pore’s inner surface. Both N_2_ and H_2_ molecules can enter the small pores, but the gas uptake for H_2_ is a factor of three larger. N_2_ is found in the pore’s centre, whereas the H_2_ molecules have their own sites close to the partially negatively charged hydrogen atoms of the BH_4_ groups. This work highlights an opportunity for the development of hydridic porous frameworks for high-density hydrogen storage or as future materials potentially having high-*T* superconductivity and stability approaching ambient conditions. The high H_2_ density in the pores is due to the anisotropic shape of the H_2_ molecules normally seen at near-ambient pressures as close-packed spheroids. In addition, at very high pressures, hydrogen molecules may show even more complex ordered patterns^22^. The association of H_2_ molecules into so-called hydrogen clusters^23^, where the intermolecular distances of H_2_ are in fact lengthened, has been reported. However, here we observe, by experiments and theoretical calculations, the formation of a cluster of five hydrogen molecules, where two are freely rotating around their centre of mass, only bonded by van der Waals interactions. This contrasts with the other three, which form a hexa-hydrogen bond, (B−H^δ−^)_2_···H−H···(H^δ−^−B)_2_. Moreover, theoretical calculations reveal that the formation of penta-dihydrogen clusters is favoured over a statistical distribution of hydrogen on the two positions. These types of hydrogen interaction are different from what was previously defined theoretically as trihydrogen bonds involving hydridic hydrogen or charge-inverted hydrogen bonds^24,25^.

## Methods

### Synthesis of magnesium borohydride

Polymorphs of magnesium borohydride were synthesized as previously described^6^. Di-butylmagnesium, Mg(*n*-Bu)_2_, was added dropwise to a solution of dimethylsulfide borane complex, (CH_3_)_2_S·BH_3_, in toluene at room temperature and under an inert atmosphere and stirring of the solution. The solution was filtered off after 2 h of stirring at room temperature using a Schlenk filtration apparatus. The white crystalline solid was subsequently washed with toluene (anhydrous, 3 × 10 ml). The obtained reaction product, magnesium borohydride hemi dimethylsulfide, Mg(BH_4_)_2_·1/2S(CH_3_)_2_, was initially dried for 12 h at room temperature on a vacuum line (2 × 10^−2^ mbar). The open structured polymorph, γ-Mg(BH_4_)_2_, was obtained by heating Mg(BH_4_)_2_·1/2S(CH_3_)_2_ at 75 °C in dynamic vacuum, whereas the more dense polymorph, α-Mg(BH_4_)_2_, was obtained by heating the solvate at 140 °C in a flow of dry argon. Both of the final reaction products consist of a major fraction of crystalline material, either γ-Mg(BH_4_)_2_ or α-Mg(BH_4_)_2_, and a minor fraction of amorphous magnesium borohydride. The amount of amorphous material can be quantified using volumetric measurements of nitrogen adsorption, as described in Supplementary Section 4.

### Hydrogen isotherm measurements

An automated Sievert’s type apparatus (PCTPro-2000) was used with a so-called micro-doser (MD) from HyEnergy. The original set-up was upgraded with a heating and cooling device to regulate the sample temperature. The adsorption and desorption isotherms (0–20 bar) were measured at various temperatures (77–298 K) in a sample cell volume of ∼1.3 ml using ultrahigh-purity hydrogen gas (99.999 %). Mg(BH_4_)_2_ (163 mg and 125 mg for γ- or α-Mg(BH_4_)_2_, respectively) was evacuated under ultrahigh vacuum at 335 K overnight before the measurements to remove any adsorbed gas from the surface. The isosteric heat of adsorption was calculated from the absolute adsorbed hydrogen according to a variant of the Clausius–Clapeyron equation (details of the calculation are provided in Supplementary Section 4).

### Cryogenic H_2_ BET measurement

The hydrogen adsorption isotherms of Mg(BH_4_)_2_ at 19.5 K were measured with laboratory-designed volumetric adsorption equipment with a temperature-controlled cryostat, as described in detail elsewhere^27^. Approximately 20 mg of γ- or α-Mg(BH_4_)_2_ was activated under ultrahigh vacuum at 335 K overnight before each measurement. For the laboratory-designed cryostat, the temperature control was calibrated by measuring the liquefaction pressure for hydrogen and nitrogen in the empty sample chamber at various temperatures.

### NPD at National Institute of Standards and Technology

NPD patterns were measured on the high-resolution powder diffractometer BT1 at the National Institute of Standards and Technology (NIST) Center for Neutron Research (NCNR). The wavelength used was *λ* = 2.074 Å. The sample was loaded into a vanadium sample holder placed inside a closed-cycle refrigerator. First, the system was evacuated overnight to empty the pores. Deuterium loading into the porous structure was performed in a two-step procedure. A known amount of gas was put into the system, which was slowly cooled to liquefaction, and then the excess deuterium was pumped off upon heating slightly above boiling point. The empty γ-Mg(^11^BD_4_)_2_ was characterized, as well as a fully D_2_-loaded framework. Both measurements were at 10 K.

### NPD at Helmholtz-Zentrum Berlin

NPD patterns were measured on the high-resolution powder diffractometer E9 at the BER-II research reactor at Helmholtz-Zentrum Berlin. The wavelength used was *λ* = 1.7982 Å. The γ-Mg(^11^BD_4_)_2_ sample was loaded into a vanadium sample holder placed inside a liquid-helium ‘Orange’ cryostat. The system was evacuated and loaded with various amounts of deuterium gas at 25 K using DEGAS gas loading systems. Four NPD patterns were collected at equilibrium pressures of 0.21, 0.45, 27 and 205 mbar at 25 K. The deuterium gas was evacuated by application of vacuum at 100 K (confirmed by a subsequent measurement of the empty porous γ-Mg(^11^BD_4_)_2_ sample) after which 3 bar of N_2_ gas was applied at 100 K.

### Analysis of diffraction data

The identification of guest molecules was done by optimization in global space, using FOX software^28^, followed by a complete Rietveld refinement in Fullprof^29,30^.

### In situ INS experiments

INS spectra were measured at the vibrational spectroscopy beamline VISION at Spallation Neutron Source, ORNL. A cryocooler cycle system was used to control the temperature of the sample in a range of 5 K to 100 K. The *p*-H_2_ was prepared by liquefying ultrahigh-purity normal hydrogen over Oxisorb (CrO nanoparticles deposited over silica) at 17 K and bleeding the vapour off this system at 22 K. The samples were loaded in aluminium containers that were connected to a gas dosing system in the beamline. The samples were dosed in situ in the cryostat at 35 K and cooled to base temperature, 5 K, for measurements. We dosed different amounts of almost pure *p*-H_2_ and normal hydrogen (*n*-H_2_). Normal hydrogen is an equilibrium mixture at room temperature consisting of 25% *p*-H_2_ and 75% *o*-H_2_.

### Computational details

The periodic density functional theory (DFT)-based calculations were carried out using the Vienna Ab initio Simulation Package (VASP)^31^. The core electrons effect was described by the projector augmented wave (PAW) method by Blöchl^32^ as implemented by Kresse and Joubert^33^. The kinetic energy cutoff for the plane-wave basis set was truncated at 500 eV. The threshold for the convergence of the electronic optimization was 10^−5^ eV, and the relaxation of the atomic positions was allowed until the forces acting on all the atoms were smaller than 0.015 eV Å^−1^. The Perdew−Burke−Ernzerhof (PBE) exchange-correlation functional^34^ was used, taking spin polarization into account. The long-range dispersion interactions were added to the PBE functional via the zero damping D3 (zero) semi-empirical methods proposed by Grimme^35^. A *Γ*-centred Monkhorst−Pack^36^ grid of 2 × 2 × 2 *k*-points in reciprocal space was used for simulation of the Mg(BH_4_)_2_ material. The γ-Mg(BH_4_)_2_ structure had the space group *Ia*$\bar{3}$*d*. Due to the computational cost, we employed the primitive cell of γ-Mg(BH_4_)_2_ available in the Materials Project Database^37^ (mp-1200811), which contains 12 magnesium, 24 boron and 96 hydrogen atoms. The host–guest interaction was obtained by subtracting the energy of isolated γ-Mg(BH_4_)_2_ and the gas-phase H_2_ molecule from the *n*-H_2_ in the γ-Mg(BH_4_)_2_ system.

### Convex hull analysis

The convex hull is a geometric construction that helps to determine the stable or equilibrium phases of a material system under specific conditions. The convex hull provides a global view of the relative stabilities of structures, allows determination of the the stability of different phases, and predicts phase transformations that may occur under certain conditions. It is useful to predict the most stable phases and identify regions where two or more phases can coexist.

The convex hull determines the thermodynamical stability of all the computed phases compared with the reference phases, plotting their energy as a function of the composition. In our case, we use the convex hull to plot the stability of H_2_ loading on γ-Mg(BH_4_)_2_. To elucidate whether different adsorptions of H_2_ are stable, we computed the energy of any configuration of H_2_ adsorbed in Mg(BH_4_)_2_ as

1 $${E}={E}({{\rm{Mg}}}({\rm{BH}}_{4})_{2}\cdot {{x}}{\rm{H}}_{2})-\frac{x}{y}E({{\rm{Mg}}}({\rm{BH}}_{4})_{2}\cdot {{y}}{\rm{H}}_{2})-\left(1-\frac{x}{y}\right)E({{\rm{Mg}}}({\rm{BH}}_{4})_{2})$$

where ${E}({{\rm{Mg}}}({\rm{BH}}_{4})_{2}\cdot {{x}}{\rm{H}}_{2})$ is the DFT total energy of the phase with a particular number of H_2_ molecules adsorbed, *E*(Mg(BH_4_)_2_) is the total DFT energy of the bare material and ${E}({{\rm{Mg}}}({\rm{BH}}_{4})_{2}\cdot {{y}}{\rm{H}}_{2})$ is the DFT total energy of the fully loaded system. In our particular case, we will use ${{E}\left({{\rm{Mg}}}({\rm{BH}}_{4})_{2}\cdot 1.33{\rm{H}}_{2}\right)}$ or ${{E}({{\rm{Mg}}}({\rm{BH}}_{4})_{2}\cdot 2.33{\rm{H}}_{2})}$ as references of the fully loaded compounds. The ${{{\rm{Mg}}}({\rm{BH}}_{4})_{2}\cdot 2.33{\rm{H}}_{2}}$ composition is the structure with the maximum loading of H_2_ molecules, and the 1.33 H_2_/Mg ratio is the structure with the fully loaded D11 site. Thus, the formation energy of the intermediately loaded phase can be calculated by reference to the fully loaded and empty material phases.

With respect to the phases with partial H_2_ loading, the negative values of *E* indicate that the structure is stable with respect to the references. However, stability with respect to the reference components is not a sufficient criterion. For overall thermodynamic phase stability, the formation energy of the particular structure of interest needs to be compared against all other phases. The convex hull connects the phases that are lower in energy than any other phase. Thus, phases that belong to the convex hull are thermodynamically stable, whereas phases with an energy above the convex hull are metastable or unstable.

Finally, note that we have plotted two convex hulls, modifying one of the references. As the energies directly depend on the compounds used as reference, the energies of the partially filled phases also change.

## Online content

Any methods, additional references, Nature Portfolio reporting summaries, source data, extended data, supplementary information, acknowledgements, peer review information; details of author contributions and competing interests; and statements of data and code availability are available at 10.1038/s41557-024-01443-x.

### Supplementary information

Supplementary Information Six chapters containing Supplementary Figs. 1–31, Tables 1–16, experimental details, methodology of DFT simulations and their analysis.

Supplementary Data 1 NPD data on Mg(^11^BD_4_)_2_ collected at 10 K under vacuum at NIST

Supplementary Data 2 NPD data on Mg(^11^BD_4_)_2_ collected at 10 K, with D_2_ at NIST

Supplementary Data 3 NPD data on Mg(^11^BD_4_)_2_ collected at 100 K, 3 bar of N_2_ at E9@HMI

Supplementary Data 4 NPD data on Mg(^11^BD_4_)_2_ collected at 25 K, 0.21 mbar of D_2_ at E9@HMI

Supplementary Data 5 NPD data on Mg(^11^BD_4_)_2_ collected at 25 K, 0.43 mbar of D_2_ at E9@HMI

Supplementary Data 6 NPD data on Mg(^11^BD_4_)_2_ collected at 25 K, 27 mbar of D_2_ at E9@HMI

Supplementary Data 7 NPD data on Mg(^11^BD_4_)_2_ collected at 25 K, 203 mbar of D_2_ at E9@HMI

### Source data

Source Data Fig. 1 Origin files

Source Data Fig. 2 Origin file

Source Data Fig. 3 Pdf in high res

Source Data Fig. 4 Pdf and Excell files, Eps and text files

Source Data Fig. 5 Pdf and Excel files, Eps and text files

Source Data Fig. 6 Excel and ppt files

## Data Availability

The data supporting the findings of this work are available within the Article, the Supplementary Information, uploaded as data files or from the corresponding authors upon request. Source data are provided with this paper.
