# Supplementary material for: Small-pore hydridic frameworks store densely packed hydrogen
Source: Nat Chem. 2024 Feb 6;16(5):809–16. doi: 10.1038/s41557-024-01443-x (PMC11087247; doi:10.1038/s41557-024-01443-x)
Supplement: Supplementary file 1 — Six chapters containing Supplementary Figs. 1–31, Tables 1–16, experimental details, methodology of DFT simulations and their analysis. [file 41557_2024_1443_MOESM1_ESM.pdf]

nature chemistry

Article https://doi.org/10.1038/s41557-024-01443-x

Small-pore hydridic frameworks store densely packed hydrogen

In the format provided by the authors and unedited

Supplementary Information for

Table of content

Chapter 1 (pages 2-6). Neutron diffraction study of the empty Mg($^{11}BD_4$)$_2$ framework, N_2-loaded γ-Mg($^{11}BD_4$)$_2$·0.66N_2 and D_2-loaded Mg($^{11}BD_4$)$_2$ framework up to the composition γ-Mg($^{11}BD_4$)$_2$·1.33D_2. Crystal structure of α- and γ–Mg(BH_4)$_2$.

Chapter 2 (pages 7-10). Re-evaluation of the synchrotron radiation X-ray powder diffraction (SR-XPD) data collected under hydrogen pressures.

Chapter 3 (pages 11-12). Neutron diffraction study of the D_2-loaded Mg($^{11}BD_4$)$_2$ framework, up to the composition γ-Mg($^{11}BD_4$)$_2$·2.33D_2.

Chapter 4 (pages 13-21). Volumetric measurements. Quantification of the crystalline fraction of γ-Mg(BH_4)$_2$ using volumetric measurements of nitrogen adsorption isotherm. Further sample analysis – possible surface oxidation. Reproducibility, repeatability, and reversibility of gas adsorption measurements. Crystal structure of α- and γ–Mg(BH_4)$_2$ and their hydrogen storage capacities.

Chapter 5 (pages 22-37). Inelastic Neutron Scattering - *In situ* study of H_2-loaded Mg($^{11}BD_4$)$_2$ framework.

- **5.1** Quantum rotation of H_2.
- **5.2** INS roto-vibrational analysis.
  - **5.2.1** Para and ortho INS spectra
  - **5.2.2** INS as function of pH_2 loading
- **5.3** Simultaneous quantum rotational excitation (SQRE).
- **5.4**. Calculation of overtone vibrational modes and SQRE.
- **5.5** Comparison with H_2 at high-pressure.
- **5.6** Density of hydrogen confined in Mg(BD_4)$_2$

Chapter 6. DFT simulations (pages 38-63).

References (page 64)

Chapter 1. Neutron diffraction study of the empty $Mg(^{11}BD_4)_2$ framework, N_2-loaded γ-$Mg(^{11}BD_4)_2 \cdot 0.66N_2$ and D_2-loaded $Mg(^{11}BD_4)_2$ framework up to the composition γ-$Mg(^{11}BD_4)_2 \cdot 1.33D_2$.

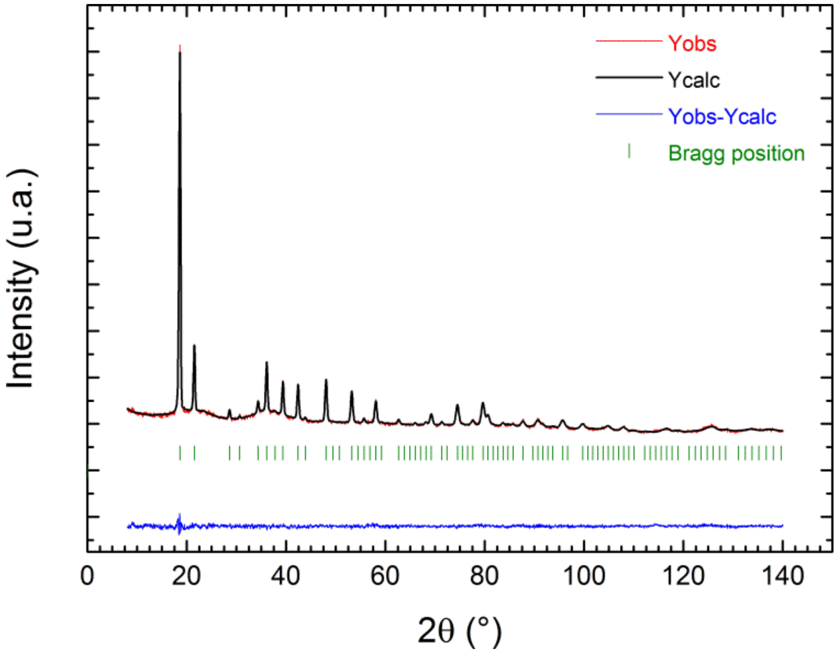

Fig. 1. Rietveld refinement profile for the 'empty-pore' γ-$Mg(^{11}BD_4)_2$ sample, using NPD data (NIST) collected at 10 K, λ = 2.079 Å.

Table 1. Structural data for 'empty-pore' γ-$Mg(^{11}BD_4)_2$ sample from NPD experiment (NIST) at 10 K, λ = 2.079 Å. Values in parentheses indicate one standard deviation.

Atom	Wyckoff site	*x*	*y*	*z*	Occupancy	B (Å²)
'Empty-pore' γ-$Mg(^{11}BD_4)_2$, space group *Ia*-3*d*, Z = 24, *a* = 15.7401(11) Å, *V* = 3899.7(5) Å^3, R_p = 23.7%, R_{wp} = 15.7%, χ^2 = 0.866, R_F = 6.07%.						
Mg	24*d*	1/4	1/8	1/2	1	1.7(2)
^{11}B	48*g*	0.3130(3)	*x*-1/4	3/8	1	1.66(9)
D1	96*h*	0.2888(3)	0.0202(3)	0.4363(4)	1	3.53(6)
D2	96*h*	0.3001(3)	0.1391(3)	0.3814(2)	1	3.53(6)

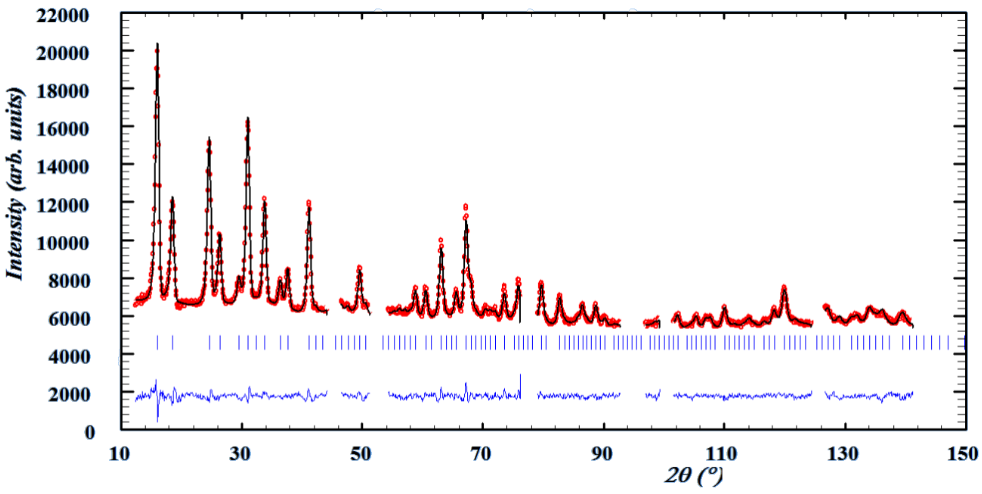

Fig. 2. Rietveld refinement profile for the nitrogen-loaded γ-Mg($^{11}BD_4$)$_2$ sample at 100 K and p(N_2) = 3 bar (HZB). The angular ranges containing contributions from the Al sample holder were excluded from the refinement, λ = 1.7982 Å.

Table 2. Structural data for the N_2-loaded γ-Mg($^{11}BD_4$)$_2$ sample from NPD experiment (HZB) at 100 K and 3 bar of gas pressure, λ = 1.7986 Å.

Atom	Wyckoff site	*x*	*y*	*z*	Occupancy	B (Å²)
N_2-loaded γ-Mg($^{11}BD_4$)$_2$, space group *Ia*-3*d*, Z = 24, *a* = 15.7498(4) Å, *V* = 3906.85(2) Å^3, R_p = 10.2%, R_{wp} = 9.25%, χ^2 = 2.17, R_F = 2.32%.						
Mg	24*d*	1/4	1/8	1/2	1	1.1(1)
^{11}B	48*g*	0.3130(2)	*x*-1/4	3/8	1	1.42(9)
D1	96*h*	0.2910(3)	0.0197(2)	0.4352(3)	1	3.21(7)
D2	96*h*	0.3015(2)	0.1402(2)	0.3817(2)	1	3.21(7)
N1	96*h*	0.6349(9)	0.6714(18)	0.6650(19)	0.1805(12)*	4.7(7)
N2	96*h*	0.628(2)	0.628(2)	0.6193(18)	0.1805(12)*	4.7(7)

* This value for the N-atoms' occupancy corresponds to 0.722(5) N_2 molecules per Mg atom. This value is correlated with the refined ADPs, and is reasonably close to the limiting composition of 0.667 N_2 molecules per Mg atom.

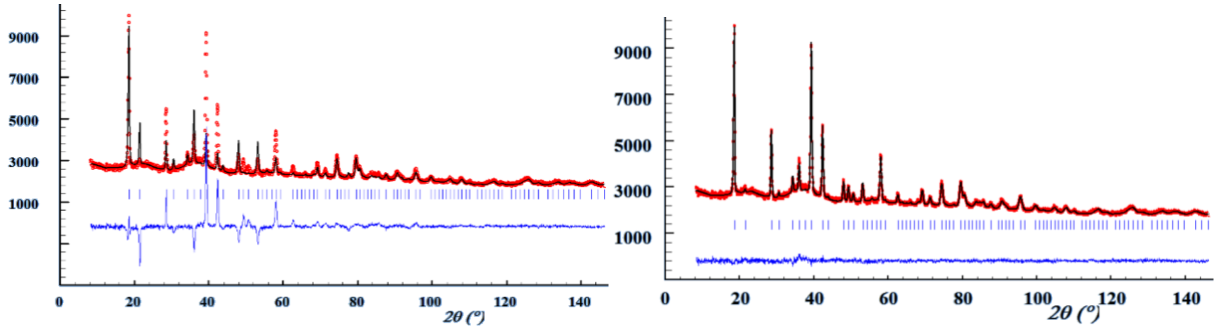

Fig. 3. Rietveld refinement profile for NPD NIST data collected at 10 K on the D_2-loaded γ-Mg(^{11}BD$_4$)$_2$ sample using: left, the structural model for nitrogen localization based on XPD data assuming the limiting composition γ-Mg(^{11}BD$_4$)$_2$·0.66D_2 (*6*), and right, the revised model that allows for the double hydrogen capacity, γ-Mg(^{11}BD$_4$)$_2$·1.33D_2. λ = 2.079 Å. This new structural model, denoted H11/D11 model, describe the hydrogen molecule as a sphere by one set of coordinates placed in the molecular centre of mass, denoted a 'superatom'.

Table 3. Structural data for D_2-loaded γ-Mg(^{11}BD$_4$)$_2$ sample from NPD experiment (NIST) at 10 K. λ = 2.079 Å.

Atom	Wyckoff site	*x*	*y*	*z*	Occupancy	B (Å²)
D_2-loaded γ-Mg(^{11}BD$_4$)$_2$, space group *Ia*-3*d*, Z = 24, *a* = 15.7424(3) Å, *V* = 3901.3(1) Å^3, R_p = 21.0%, R_{wp} = 15.4%, χ^2 = 0.889, R_F = 4.83%.						
Mg	24*d*	1/4	1/8	1/2	1	0.8(1)
^{11}B	48*g*	0.3115(3)	*x*-1/4	3/8	1	0.1(1)
D1	96*h*	0.2886(3)	0.0195(3)	0.4349(4)	1	2.90(7)
D2	96*h*	0.3008(3)	0.1375(3)	0.3800(2)	1	2.90(7)
D11	32*e*	0.6843(2)	*x*	*x*	2.156(18)*	11.1(3)

* This value for the D_2 superatom occupancy corresponds to 1.078(9) occupancy of the individual atoms. This value is slightly over 1 due to a correlation in the ADP (refined B = 11.2(3) Å^2), which falls within the expected range for the "superatom" model. When the B-factor is fixed to 8, the occupancy of the "superatom" refines exactly to 1. This corresponds to 1.33 D_2 per Mg atom.

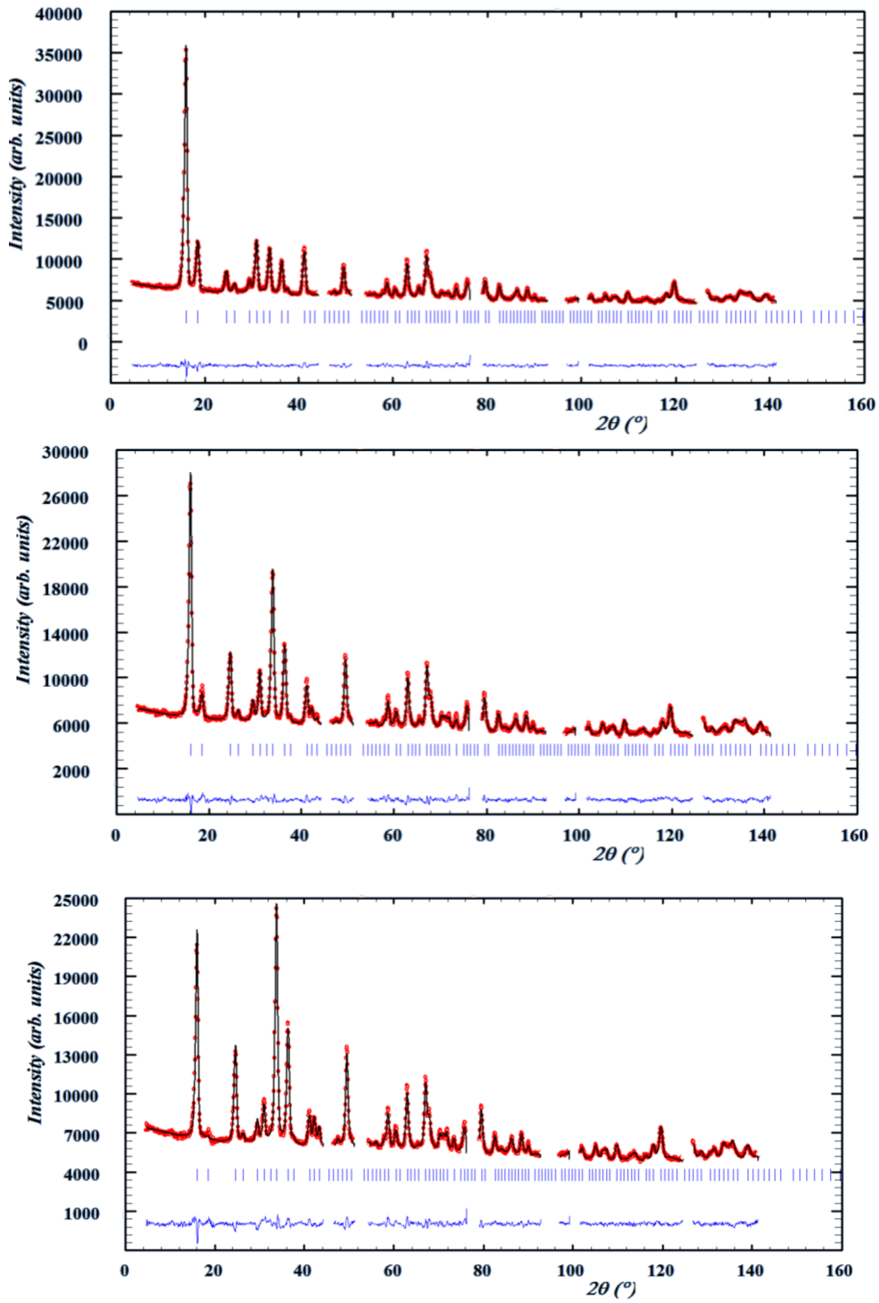

Fig. 4. Rietveld refinement profiles for the deuterium-loaded γ-Mg($^{11}BD_4$)$_2$ at 25 K and $p(D_2)$ = 0.21 mbar (top), 0.43 mbar (middle), 27 mbar (bottom) equilibrium pressures (HZB), refined with one superatom D_2 site. The angular ranges containing contributions from the Al sample holder were excluded from the refinement. λ = 1.7982 Å.

Table 4. Structural data for the D_2-loaded γ-$Mg(^{11}BD_4)_2$ from NPD experiment (HZB) at 25 K and 0.21 mbar, 0.43 mbar, 27 mbar equilibrium pressures, refined with one superatom (centroid) D_2 site. λ = 1.7982 Å.

Atom	Wyckoff Site	*x*	*y*	*z*	Occupancy	B (Å²)
γ-$Mg(^{11}BD_4)_2 \cdot 0.35D_2$ at 25 K and 0.21 mbar D_2, space group *Ia*-3*d*, Z = 24, *a* = 15.7678(4) Å, *V* = 3920.2(2) Å³, R_p = 9.5%, R_{wp} = 8.26%, χ^2 = 2.21, R_F = 2.10%.						
Mg	24*d*	1/4	1/8	1/2	1	0.9(1)
^{11}B	48*g*	0.31256(19)	*x*-1/4	3/8	1	0.78(7)
D1	96*h*	0.2903(2)	0.0204(2)	0.4354(3)	1	2.55(5)
D2	96*h*	0.3014(2)	0.1399(2)	0.38137(18)	1	2.55(5)
D11	32*e*	0.6597(5)	*x*	*x*	0.526(8)*	11.2
γ-$Mg(^{11}BD_4)_2 \cdot 1.05D_2$ at 25 K and 0.43 mbar D_2, space group *Ia*-3*d*, Z = 24, *a* = 15.7687(4) Å, *V* = 3920.9(2) Å³, R_p = 10.5%, R_{wp} = 9.31%, χ^2 = 2.75, R_F = 2.89%.						
Mg	24*d*	1/4	1/8	1/2	1	1.3(1)
^{11}B	48*g*	0.31091(19)	*x*-1/4	3/8	1	0.65(7)
D1	96*h*	0.2890(2)	0.0188(2)	0.4358(3)	1	2.73(5)
D2	96*h*	0.3004(2)	0.1383(2)	0.3815(2)	1	2.73(5)
D11	32*e*	0.6791(2)	*x*	*x*	1.576(7)*	11.2
γ-$Mg(^{11}BD_4)_2$ at 25K and 27 mbar D_2, space group *Ia*-3*d*, Z = 24, *a* = 15. 7741(4) Å, *V* = 3925.0(2) Å³, R_p = 10.9%, R_{wp} = 9.71%, χ^2 = 2.99, R_F = 2.33%.						
Mg	24*d*	1/4	1/8	1/2	1	1.0(1)
^{11}B	48*g*	0.3116(2)	*x*-1/4	3/8	1	0.42(7)
D1	96*h*	0.2891(2)	0.0184(2)	0.4355(3)	1	2.59(6)
D2	96*h*	0.3007(3)	0.1383(2)	0.3804(2)	1	2.59(6)
D11	32*e*	0.6849(2)	*x*	*x*	2.29(1)*	11.2

* The value of the superatom occupancy corresponds to double occupancy of the individual D-atoms. The full site occupation (2 for the superatom) corresponds to 1.33 D_2 per Mg atom.

Chapter 2. Re-evaluation of the synchrotron radiation X-ray powder diffraction (SR-XPD) data collected under hydrogen pressures.

The new H_2 localization obtained from the NPD experiments (denoted H11/D11) allowed us to re-evaluate the *in situ* SR-XPD data collected on γ–$Mg(BH_4)_2$ under different H_2 pressures and temperatures (*6*). X-ray diffraction did not allow to localize hydrogen molecules in the porous γ–$Mg(BH_4)_2$ structure, so it was assumed that hydrogen was placed at the same position as nitrogen. Although such XPD data were fitted well using the 'nitrogen structural model' for localizing hydrogen (see Fig. 5a), but the quality of the Rietveld refinement was improved considerably using the new H11/D11-model extracted from NPD data and the same SR-XPD data (see Fig 5b): χ^2 decreased to less than half the original value, from 1310 to 610. Importantly, the new geometric (H11/D11) model corrects the original estimate of the maximum experimental hydrogen content from γ–$Mg(BH_4)_2\cdot 0.80H_2$ (*6*) to γ–$Mg(BH_4)_2\cdot 1.32H_2$ (this work). The obtained value is very close to the values obtained from NPD data and to the value expected at full occupancy of the H_2 site (1.33 H_2 per Mg). Structural parameters for the H11/D11 model refined using SR-XPD data measured at 80 K and 105 bar of H_2 are listed in Table 5.

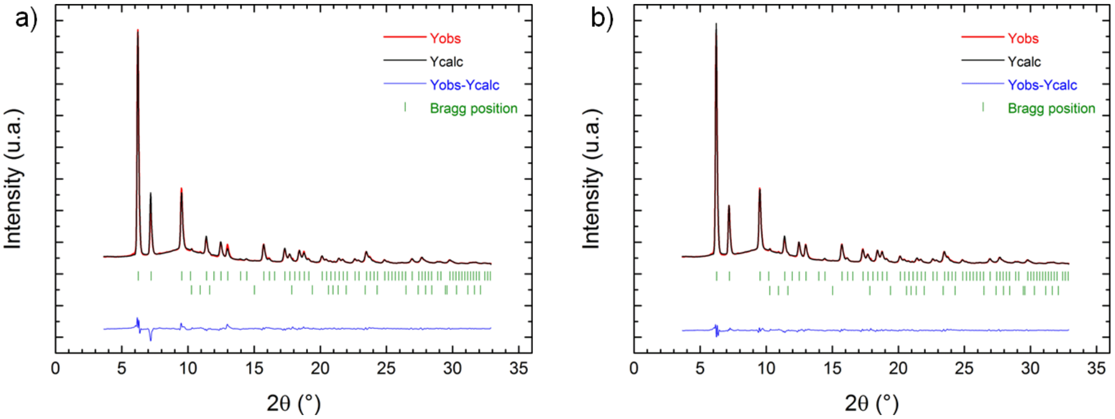

Fig. 5. Rietveld refinement profile for the SR-XPD data collected on γ-$Mg(BH_4)_2$ at 80 K and 105 bar of H_2 using a) the X-ray model for nitrogen localization used previously in Ref (*6*), b) the revised NPD model, giving the resulting composition γ–$Mg(BH_4)_2\cdot 1.32H_2$. The lower line of Bragg reflection tick marks refer to ice forming on the outer side of the cold glass capillary. λ = 0.700930 Å.

Table 5. Structural parameters for γ-$Mg(BH_4)_2$ exposed to $p(H_2)$ = 105 bar at 80 K, using SR-XPD data and the revised model of hydrogen localization. Nearly double occupancy of H_2 "superatom" indicates the full occupation of the site and thus the composition γ−$Mg(BH_4)_2\cdot 1.32H_2$. λ = 0.700930 Å.

Atom	Wyckoff site	*x*	*y*	*z*	Occupancy	B (Å²)
Space group *Ia*-3*d*, Z = 24, *a* = 15.7771(6) Å, *V* = 3927.1(3) $Å^3$<br>R_p = 8.3%, R_{wp} = 8.8%, χ^2 = 610, R_F = 2.2%.						
Mg	24*d*	1/4	1/8	1/2	1	0.9(1)
B	48*g*	0.30981	*x*-1/4	3/8	1	0.9(1)
H1	96*h*	0.28571	0.02071	0.43730	1	2.9(1)
H2	96*h*	0.29741	0.13551	0.13551	1	2.9(1)
H11	32*e*	0.6843	*x*	*x*	1.98(4)*	16.9(1)

* This value for the H_2 superatom occupancy practically corresponds to full occupancy of the site by H_2 molecules, the latter corresponds to 1.33 H_2 per Mg atom.

Hydrogen (H_2) and nitrogen (N_2) adsorption in γ-$Mg(BH_4)_2$ was previously investigated by synchrotron radiation X-ray powder diffraction (SR-XPD), but this data only allow to localize nitrogen (*6*). Thus, during Rietveld refinement of the X-ray data hydrogen was assumed to be placed at the same crystallographic position as nitrogen. Below, we re-analyse this data using the previous 'N_2-model' for hydrogen and compare that to results using the new 'D_2-model' (with D11 site).

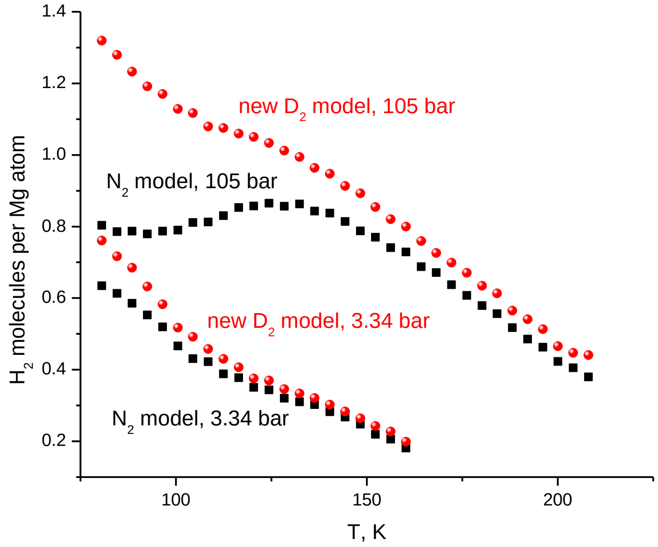

Fig. 6. Hydrogen adsorption isobars for γ-$Mg(BH_4)_2$ extracted from *in situ* powder X-ray diffraction data (*6*) using the previous structural model based on localisation of nitrogen (N_2-model) (black squares) and the new NPD model (with D11 sites, red circles). Note that there is no decrease in adsorbed amount of H_2 at lower temperature when using the new H11/D11 model.

Using the new H11/D11 model and previously reported *in situ* SR-XPD data measured at 3.34 bar and 105 bar, the H_2 site occupancies were refined as a function of temperature. Fig. 6 shows the amount of H_2 adsorbed per Mg atom, both for the old (black squares) and the new (red circles) models. The major differences are observed for the temperatures below 150 K, where an unexplained decrease in capacity is eliminated using the new D11 model.

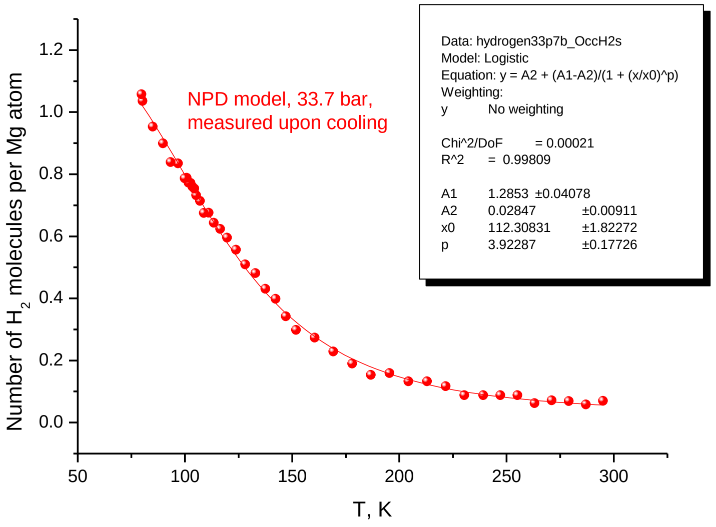

Fig. 7. Amount of adsorbed hydrogen in γ-$Mg(BH_4)_2$ extracted from *in situ* SR-XPD data collected at constant pressure of $p(H_2)$ = 33.7 bar and temperature varied from 80 to 300 K. The fit of the logistic function to the experimental isobar yields the H_2 capacity per Mg atom at 1.29(4), which suggest an almost fully occupied site (1.33).

We employed the revised H11/D11 model to characterize hydrogen adsorption at 33.7 bar over a wide range of temperatures, from liquid nitrogen to ambient, using previously unpublished SR-XPD data. The fit of the logistic function to the data is shown in Fig. 7, although only a part of the Sigmodal-curve was measured. The H_2 capacity per Mg atom (denoted A1 in the logistic model) on saturation refines to, A1 = 1.29(4), which suggest an almost fully occupied H11/D11 site (1.33). Very similar values are obtained at higher pressure, A1 = 1.30(2) at $p(H_2)$ = 105 bar, and even for the low pressure of $p(H_2)$ = 3.34 bar, A1 = 1.4(2). The latter two values of A1 are extracted using the data shown in Fig. 6. It is highly satisfactory that the saturation limit can be extrapolated so well using the XPD-adsorption data covering only the lower half of the loading curve at 3.34 bar. Although the uncertainties become higher at lower pressures, the fit of the logistic function to the data allows estimation of the limiting adsorption capacity. The A2 parameter reflects the capacity on complete desorption, which is indeed zero within three standard uncertainties. The temperature at which half of the maximum hydrogen adsorption capacity is adsorbed is $x_0 = 112 \pm 2$ K.

Chapter 3. Neutron diffraction study of the D_2-loaded $Mg(^{11}BD_4)_2$ framework, up to the composition of γ-$Mg(^{11}BD_4)_2{\cdot}2.33D_2$.

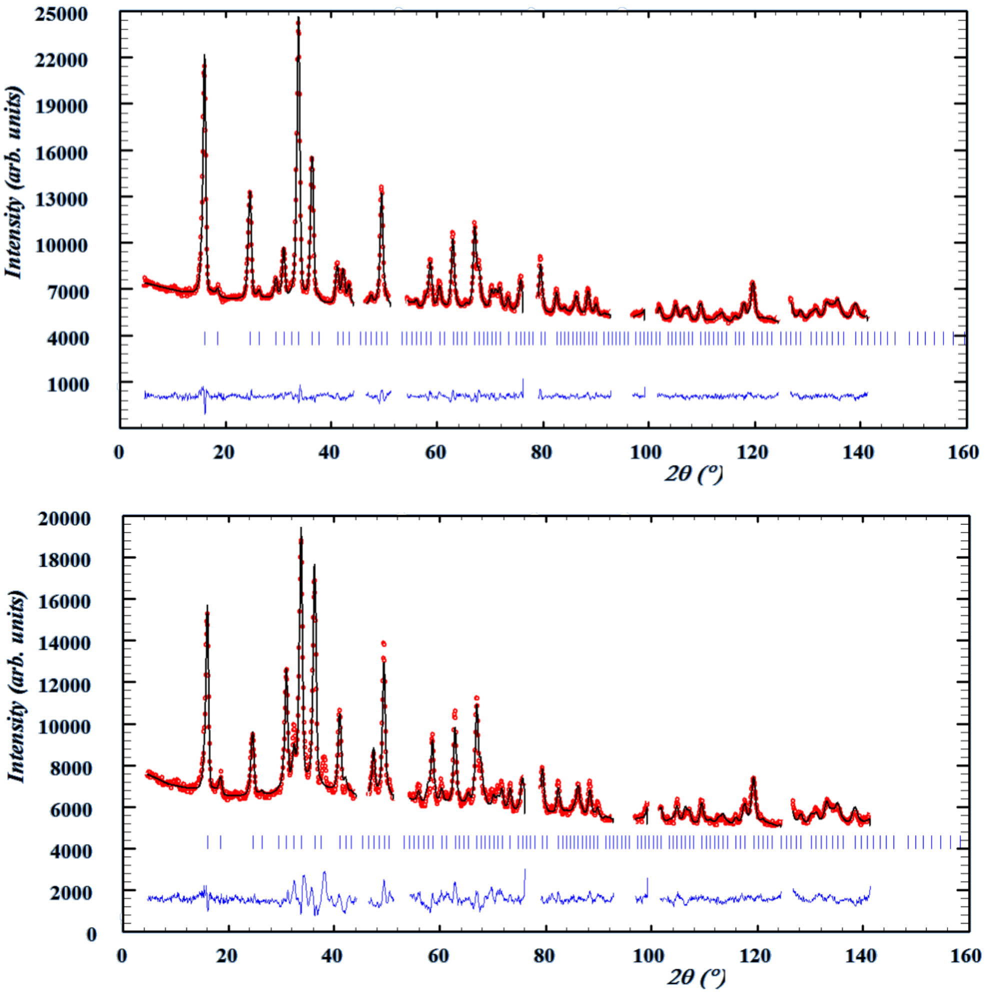

Fig. 8. Rietveld refinement profiles for the deuterium-loaded γ-$Mg(^{11}BD_4)_2$ sample at 25 K and 27 mbar (top) and 203 mbar (bottom) equilibrium pressures (HZB), refined with two superatom D_2 sites (D11 and D22). The angular ranges containing contributions from the Al sample holder were excluded from the refinement. λ = 1.7982 Å.

Table 6. Structural data for the D_2-loaded γ-Mg($^{11}BD_4$)$_2$ from NPD experiment (HZB) at 25 K and 27 mbar and 203 mbar equilibrium pressures, refined with two superatom D_2 sites (D11 and D22). λ = 1.7982 Å. The last column indicates the amount of D_2 molecules per Mg atom, calculated by multiplying the Wyckoff site multiplicity by the occupancy factor. The limiting amount of D11 occupation is 1.33 D_2/Mg at saturation, and of D22 occupation is 1.00 D_2/Mg.

Atom	Wyckoff Site	*x*	*y*	*z*	Occupancy	B (Å²)	D_2/Mg
γ-Mg($^{11}BD_4$)$_2$·1.47D_2 at 25 K and 27 mbar D_2, space group *Ia*-3*d*, Z = 24, *a* = 15.7738(3) Å, *V* = 3924.7(1) Å^3, R_p = 9.81%, R_{wp} = 8.57%, χ^2 = 2.39, R_F = 2.12%.							
Mg	24*d*	1/4	1/8	1/2	1	1.1(1)	
^{11}B	48*g*	0.31105(19)	*x*-1/4	3/8	1	0.70(7)	
D1	96*h*	0.2888(2)	0.0185(2)	0.4357(3)	1	2.94(6)	
D2	96*h*	0.2998(3)	0.1386(2)	0.3811(2)	1	2.94(6)	
D11	32*e*	0.68489(18)	*x*	*x*	2.183(14)*	10.6(2)	1.46
D22	48*g*	0.5310(19)	0.7190(19)	5/8	0.141(7)*	10.6(2)	0.14
γ-Mg($^{11}BD_4$)$_2$·2.33D_2 at 25 K and 203 mbar D_2, space group *Ia*-3*d*, Z = 24, *a* = 15.8039(7) Å, *V* = 3947.3(3) Å^3, R_p = 18.7%, R_{wp} = 17.1%, χ^2 = 6.72, R_F = 6.05%.							
Mg	24*d*	1/4	1/8	1/2	1	1.4(2)	
^{11}B	48*g*	0.3132(3)	*x*-1/4	3/8	1	0.93(9)	
D1	96*h*	0.2903(4)	0.0212(4)	0.4333(5)	1	3.10(8)	
D2	96*h*	0.2996(4)	0.1388(4)	0.3812(3)	1	3.10(8)	
D11	32*e*	0.6947(4)	*x*	*x*	2.05(3)*	16.5(6)	1.37
D22	48*g*	0.5434(6)	0.7066(6)	5/8	0.971(13)*	16.5(6)	0.97

* The value of the superatom occupancy corresponds to double occupancy of the individual D-atoms. The refined occupancies of the two superatom sites correspond to 1.47 D_2 per Mg atom (assuming the full occupancy for D11) at 27 mbar equilibrium pressure and to 2.33 D_2 per Mg atom (assuming the full occupancy of D11 and half occupancy for D22) at 203 mbar equilibrium pressure.

# Chapter 4. Volumetric measurements.

Quantification of the fraction of crystalline γ-$Mg(BH_4)_2$ using nitrogen adsorption isotherms.

During the synthesis procedure of γ-$Mg(BH_4)_2$ at elevated temperatures (75 °C when the template molecule $(CH_3)_2S$ is removed), already a fraction of the material is amorphized. However, this fraction is staying constant over the timescale of our experiments. Even after long-term storage for several months at room temperature, the amorphization process continues to proceed extremely slowly. Some samples are amorphisizing faster than others. This difference may be due to different amounts of residual solvent remaining in the sample, which may eventually catalyse the transformation. This transformation is independent of exposure to air. The slow amorphization process is attributed to the conversion into a denser amorphous phase. The γ-$Mg(^{11}BD_4)_2$ used in NPD experiments did not show a noticeable amorphization even 5 years after the initial experiments. Pressure-induced amorphization is well described in the literature as a collapse of the very open structure of γ-$Mg(BH_4)_2$ (*38*) and is also known for MOF type materials.

The amorphization also strongly affects the gas adsorption properties for the bulk γ-$Mg(BH_4)_2$ sample. Since our bulk samples of γ-$Mg(BH_4)_2$ contain different fractions of crystalline porous phase depending on the synthesis and duration of storage, we have to quantify this fraction, which is responsible for the gas uptake. This can be performed by a combination of neutron, X-ray diffraction data, and N_2 adsorption experiments. The crystalline, open structure of γ-$Mg(BH_4)_2$ adsorbs 2/3 N_2 molecule per Mg atom as demonstrated by diffraction experiments whereas the amorphous, collapsed phase shows no gas uptake. Therefore, we attribute the amount of nitrogen adsorbed, as determined from a volumetric isotherm, solely to the adsorption that occurs within the crystalline γ-$Mg(BH_4)_2$ structure. Direct comparison of N_2 and H_2 isotherms of bulk γ-$Mg(BH_4)_2$ measured on the same sample batch are shown in Fig. 4 in the manuscript. It is important to note that the difference in H_2 or N_2 uptake between a) short-term (3~4 weeks) storage and b) long-term (approximately 12 months) storage shown in Fig. 4 is solely due to the aging time of the sample during storage. By converting the volumetric N_2 uptake at saturation to the number of N_2 molecules per Mg in crystalline and porous γ-$Mg(BH_4)_2$ (2/3 N_2 per Mg), it is possible to estimate the amorphous fraction of $Mg(BH_4)_2$ in a bulk sample. Remarkably, the volumetric H_2 uptake corrected for the amorphous phase consistently exhibits γ-$Mg(BH_4)_2 \cdot 2.04\sim2.06\ H_2$, very close to the limiting composition obtained from the crystallographic data (γ−$Mg(BH_4)_2 \cdot 2.33H_2$).

Further sample analysis – possible surface oxidation. Surface contamination or oxidation of γ-$Mg(BH_4)_2$ can be excluded during the physisorption measurements of hydrogen since both γ-$Mg(BH_4)_2$ and hydrogen are known to be very unreactive at these low temperatures. The gasses used for adsorption measurements are of very high purity (H_2 99.999 %), without any trace of oxygen and water. Furthermore, $Mg(BH_4)_2$ does not spontaneously react with oxygen when exposed to air, but it is a hygroscopic material that absorbs water.

Reactions between borohydrides and possible surface oxides or hydroxides have not previously been observed at low temperatures. Also, the samples described in Ref. (*38*) were studied for oxidation by EDX compositional mapping (see Fig. 2 in Ref. (*38*)), showing only minor oxidation in the thin surface layer. Since the samples in the present work are prepared in the same laboratory by the same protocols as in Ref. (*38*), this can be seen as sufficient evidence of no surface oxidation.

Reproducibility, repeatability, and reversibility of gas adsorption measurements. All adsorption measurements have been followed directly by a desorption branch showing negligible hysteresis. Several volumetric hydrogen physisorption measurements have been repeated on the same sample to confirm the repeatability and reversibility of the adsorption process. The 77 K isotherm was measured with different pressure step sizes and no difference in uptake was observed, which demonstrates clearly that the isotherms are in equilibrium. Furthermore, this cycling without loss of uptake rules out any surface oxidation or sample degradation during the timescale of these measurements.

Crystal structure of α- and γ–Mg(BH₄)₂ and their hydrogen storage capacities. The highly symmetric cubic crystal structure of γ–$Mg(BH_4)_2$ (space group *Ia*-3*d*), which possesses a 3D net of interpenetrated channels is shown in Fig. 9a. The B-$H^{\delta-}$ bonds contribute to forming a partly negatively charged interior of γ–$Mg(BH_4)_2$. The narrowest part of the channels, 5.8 Å in diameter, is defined by the space between the hydrogen atoms of BH_4 groups that comprise the walls of the channel. γ–$Mg(BH_4)_2$ contains 33 % empty space in the structure, suggesting a pronounced gas adsorption potential for this material.

It was previously discovered that nitrogen molecules are located at the centre of the pore of γ–$Mg(BH_4)_2$, i.e. at the crystallographic position (0.125, 0.125, 0.125). As pointed out in this work, *hydrogen has other adsorption sites*. Initially, the D11 position is occupied, which is the crystallographic 32*e* position situated on the cubic diagonal, i.e. the 3-fold-axis, where $x = y = z$. This position is significantly shifted from the centre towards the apertures. At higher loadings, hydrogen also occupies the D22 position, which is the crystallographic 48*g* site placed on the 2-fold axis. Both sites can be seen as special positions, i.e. positioned on the point group symmetry elements, but they have independent parameters for refinement. The occupancy of the D11 position reaches 100 % at full hydrogen loading at sufficiently low *T* and sufficient hydrogen partial pressure. In contrast, the D22 has an occupancy of 50 % at full loading due to a short mutually exclusive distance between two symmetry-equivalent D22 positions. The symmetry of the γ–$Mg(BH_4)_2$ structure is very complex due to a high number of crystallographic positions with different multiplicities.

Another less porous hexagonal polymorph of magnesium borohydride, α–$Mg(BH_4)_2$ (space group $P6_122$), is also investigated in this study, and its structure is illustrated in Fig. 9b. This polymorph contains unoccupied voids accounting for 6.4 vol% of the structure. The volume of each void compares approximately to the size of a water molecule (*39*).

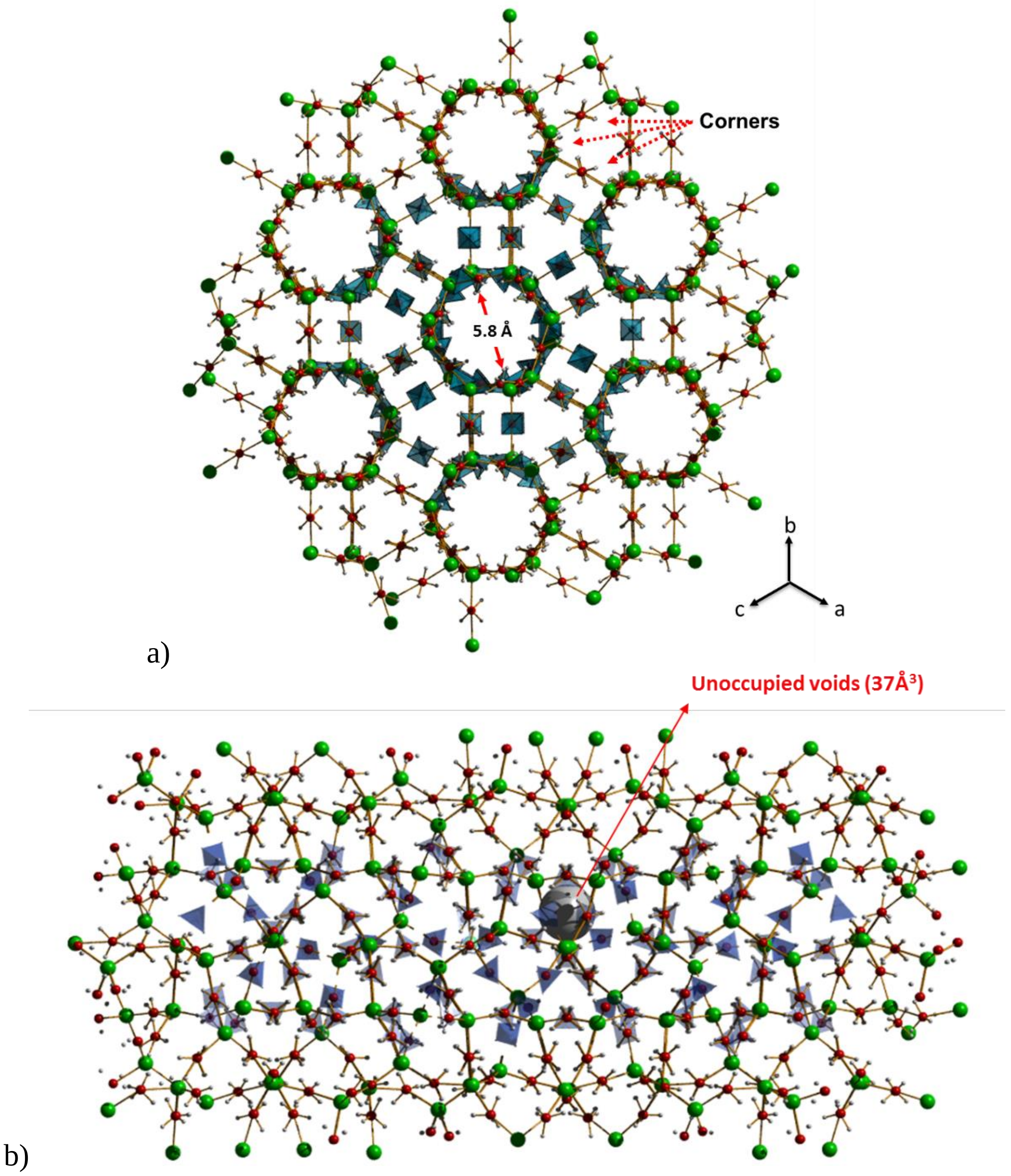

Fig. 9. The structure of **a)** γ-$Mg(BH_4)_2$ and **b)** α-$Mg(BH_4)_2$. The structure of γ-$Mg(BH_4)_2$ contains empty channels with an aperture of 5.8 Å and the empty volume accounts for ~33 % of the space in the structure. The structure of α-$Mg(BH_4)_2$ contains unoccupied voids of 37 Å^3 accounting for 6.4 % of the space in the structure (*39*). Symbols: Mg (green), B (red), and H (gray). The BH_4 group inside the unit cell is shown as a dark blue tetrahedral.

High-pressure hydrogen adsorption isotherms. Excess hydrogen isotherm adsorption curves of the α– and γ–$Mg(BH_4)_2$ measured at 77 K and 298 K are shown in Fig. 10. The data measured for γ–$Mg(BH_4)_2$ at 77 K exhibits the characteristic IUPAC type-I curve shape, which is typical for microporous materials. The maximum measured excess hydrogen uptake of γ–$Mg(BH_4)_2$ is 3.4 mass fraction (wt%) and 0.12 mass fraction (wt%) H_2 at 77 K and room temperature (RT), $p(H_2)$ = 20 bar, respectively. This is in agreement with the high porosity observed by crystallographic investigations, which reveals a 3D net of interpenetrated channels having a wide mean pore diameter (see Table 7).

In contrast, the excess hydrogen uptake of the polymorph α–$Mg(BH_4)_2$ is significantly lower over the entire pressure range at either temperature. α–$Mg(BH_4)_2$ only absorbs 0.26 mass fraction (wt%) H_2 at 77 K, $p(H_2)$ = 20 bar, which compares to 0.07 H_2 molecule per Mg atom. According to previous crystallographic investigations (*39*), the unoccupied voids (37 Å^3) might be large enough to accommodate a small molecule, such as H_2O (kinetic diameter ~2.6 Å). However, this theoretical value was obtained using a probe 'atom' with a radius of 1.0 Å and may be a suitable predictor of the hydrogen adsorption isotherm since the typical range of intermolecular H···H interaction in α–$Mg(BH_4)_2$ is around 2.7 Å to 2.9 Å. Therefore, the void windows are smaller or may be similar in size to the kinetic diameter of a hydrogen molecule, i.e. 2.8 Å to 2.9 Å. Thus, the small pore size in α–$Mg(BH_4)_2$ may pose a strong kinetic barrier, leading to diffusion-limited hydrogen uptake and release in this material. It is notable though, that the well-studied α–$Mg(BH_4)_2$ demonstrates hydrogen adsorption in the small pores, making it the second nano-porous hydride after γ–$Mg(BH_4)_2$ to do so.

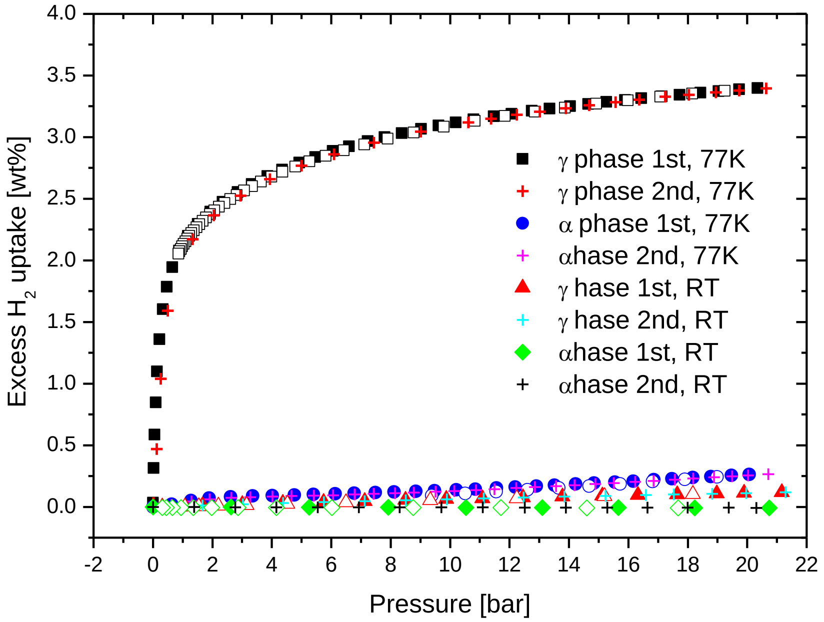

Fig. 10. Reversibility of hydrogen uptake and release for α and γ–$Mg(BH_4)_2$ samples; squares, circles, triangles and diamond stand for 77 K - γ–$Mg(BH_4)_2$, 77 K - α–$Mg(BH_4)_2$, RT - γ–$Mg(BH_4)_2$ and room temperature (RT) - α–$Mg(BH_4)_2$, respectively. Crosses indicate the second measurement of all samples. Closed symbols show the adsorption isotherm and open symbols show the desorption isotherm. Prior to the measurement, the samples were evacuated in high vacuum at room temperature overnight, as described above.

Table 7. Structural characteristics of the porosity of α- and γ–$Mg(BH_4)_2$.

	N_2 BET [a] (m²/g)	H_2 BET [b] (m²/g)	H_2 Uptake [c] (wt.%)	ρ [d] (g/cm³)	Pore window [e] (Å)	Pore Diameter (Å)
γ – $Mg(BH_4)_2$	610	1787	4.2	0.55	5.8	7.0 [f]
α – $Mg(BH_4)_2$	-	-	-	0.78	2.8	4.1 [g]

[a] **N_2 BET**: nitrogen BET specific surface area at 77 K ($p/p_0 = 3\times10^{-5}$~0.02), [b] **H_2 BET**: hydrogen BET specific surface area at 20 K ($p/p_0 = 5\times10^{-4}$~0.02) using a cross-sectional area of the hydrogen molecule based on the liquid density, [c] **H_2 uptake**: Saturated hydrogen uptake at 19.5 K and 0.7 bar, [d] **ρ**: Skeletal density, [e] **Pore window**: Narrowest channel as pore window considering the van der Waals radius of atoms in the framework, [f] **Pore diameter**: calculated by BJH Adsorption dV/dD pore volume distribution, [g] **Pore diameter**: calculated from crystallographic data.

Calculation of excess adsorption. To remove an influence of the temperature gradient between the cooled sample cell and gas reservoir with a pressure transducer (298 K) during isotherm measurement in the temperature range 77 to 127 K, non-adsorbing samples (sea sand) possessing the same volume were measured under identical conditions. By subtracting these two measurements, the influence of the temperature gradients as well as systematic errors cancels out. Thus, the excess amount of hydrogen $n_{excess}(p,T)$ adsorbed can be calculated by:

$$n_{\mathrm{excess}}(p,T) = n_{\mathrm{experiment}}(p,T) - n_{\mathrm{sea\ sand}}(p,T)$$

The adsorbed amount is reported in wt% defined as the mass of hydrogen m_{ads} per mass of the system, which consists of the sample mass m_S and the adsorbed hydrogen.

$$\text{Hydrogen uptake (wt\%)} = \frac{m_{\mathrm{ads}}}{m_s + m_{\mathrm{ads}}}$$

Calculation of absolute adsorption from excess adsorption. The excess uptake should be corrected in order to exactly represent the amount of hydrogen adsorbed on the surface, and that is the so-called absolute uptake. For estimating the absolute amount adsorbed, the volume of the adsorbed layer (V_{ads}) needs to be known. The density of the adsorbed layer is close to the density of the liquid ρ_{lq} and therefore the volume of the adsorbed layer is approximately

$$V_{ads} = \frac{n_{excess} \cdot M_{H2}}{\rho_{lq}}$$

where M_{H2} is the molar mass of hydrogen.

If there was no adsorption, in the volume of the adsorbed layer the amount (n_{gas}) of gas would be present due to the external pressure. This can be calculated from the real gas equation

$$n_{gas} = \frac{P \cdot V_{ads}}{Z \cdot R \cdot T_{Cold}}$$

with R the gas constant, Z the correction factor for non-ideal gas

Z = (1.000547- (6.07•10^{-7})T+(0.000912-(1.0653•10^{-6})T)•P+((7.373407-0.0901T)•10^{-7})•P^{2}) at temperature T(K) and pressure P(atom).

The absolute amount of adsorbed gas $\boldsymbol{n_{abs}}$ is therefore:

$$\boldsymbol{n_{abs} = n_{gas} + n_{excess} = n_{excess} \cdot (1 + \frac{P \cdot M_{H2}}{Z \cdot \rho_{lq} \cdot R \cdot T_{Cold}})}$$

Evaluation of the isosteric heat of adsorption. The isosteric heat of adsorption is typically calculated from hydrogen isotherm measurements, e.g. at liquid nitrogen (77 K) and liquid Ar (87 K) temperature. This simple calculation, however, results in a high uncertainty due to the small temperature range. Therefore, hydrogen adsorption isotherms for γ–Mg(BH$_4$)$_2$ was measured over a wider temperature range within 77 to 297 K, allowing the determination of the heat of adsorption for a wide range of surface coverage into minimize the uncertainty.
Fig. S11 shows the temperature variation of the excess hydrogen adsorption curves, which provides the strength of the binding potential for hydrogen in γ–Mg(BH$_4$)$_2$. The steep initial increase of H_2 adsorption indicates a strong interaction between hydrogen and the scaffold, and therefore saturation is reached at low pressure. The isosteric heat of adsorption is calculated from the absolute adsorption isotherms and the Clausius-Clapeyron equation at different temperatures (77 K to 117 K). In Fig. 5, the isosteric heat of adsorption is shown. Analysis of the hydrogen adsorption enthalpy gives a maximum value of 6.1 kJ/mol at near zero surface coverage, decreasing to 4.5 kJ/mol with increasing H_2 loading.

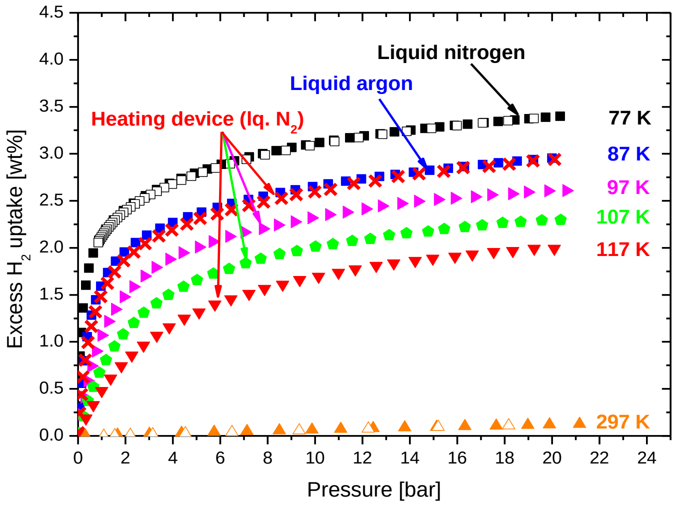

Fig. 11. Excess hydrogen uptake in γ–Mg(BH$_4$)$_2$ at temperatures between 77 K and room temperature. Filled symbols for adsorption, and open symbols for desorption.

The isosteric heat of adsorption is calculated from the measured absolute isotherms according to

$$\Delta H = R \cdot \left(\frac{\partial \ln(P)}{\partial \frac{1}{T}}\right)_{\theta}$$

where θ is the surface coverage, R is the gas constant, P is the pressure, and T the temperature. Therefore ln(P) is plotted versus the reciprocal temperature $1/T$ for different surface coverage θ. This is shown in Fig. 12 for γ–$Mg(BH_4)_2$. The slope of the linear fit to this data for each surface coverage θ is proportional to the isosteric heat of adsorption.

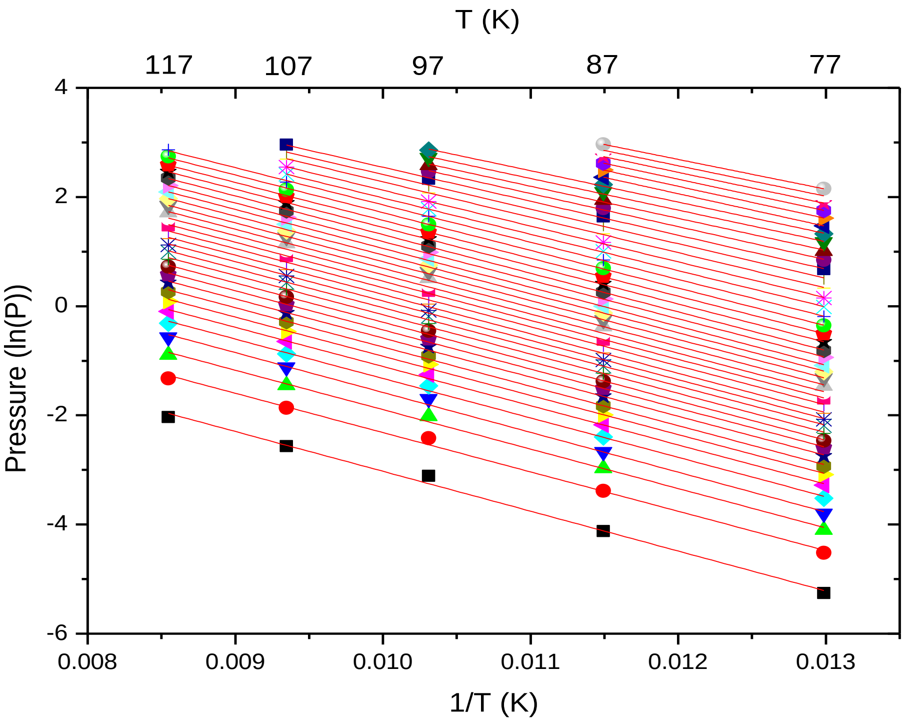

Fig. 12. The plot of ln(P) versus $1/T$ for γ–$Mg(BH_4)_2$–H_2 system.

***Hydrogen sorption isochoric measurement; Temperature dependent pressure change*.**

To understand in detail the hydrogen kinetic effect from the pore window of α- and γ–$Mg(BH_4)_2$, it is important to investigate the influence of temperature in the hydrogen storage system. Therefore, the activated material in the sample holder is loaded with 802 mm Hg hydrogen at room temperature and the connection valve is closed so that the volume available for the gas remains constant. Then, the sample holder is cooled to approximately 11 K where hydrogen should be liquid or solid. The pressure is monitored while the sample is heated to different intermediate temperatures; each temperature is kept constant for approximately 2 min and then the pressure is recorded.

For the empty sample holder, the pressure is almost 0 mmHg at around 11 K as the hydrogen in the sample holder is liquefied. Slightly above 15 K, the phase transition from liquid to gas is observed by a sudden increase of the pressure in the sample holder. Then, the slope becomes smaller with increasing temperature as the hydrogen remains in the gaseous phase and the pressure increases only due to thermal expansion, as shown in Fig. S13.

With γ–$Mg(BH_4)_2$ in the sample holder, the pressure develops differently. The pressure remains constant (~0 mm Hg) up to approximately 30 K. Afterwards, the pressure increases linearly until approximately 140 K. Thereafter the pressure matches that of the empty sample holder. This

different pressure behaviour is mainly caused by hydrogen adsorption on the material. At higher temperatures, hydrogen starts to desorb, resulting in increased pressure in the sample holder.
In contrast, for α-$Mg(BH_4)_2$ the pressure develops similarly to that observed using an empty sample holder. This means that the pores inside α-$Mg(BH_4)_2$ are not accessible for hydrogen and the small pore window is effectively closed even at high temperatures.

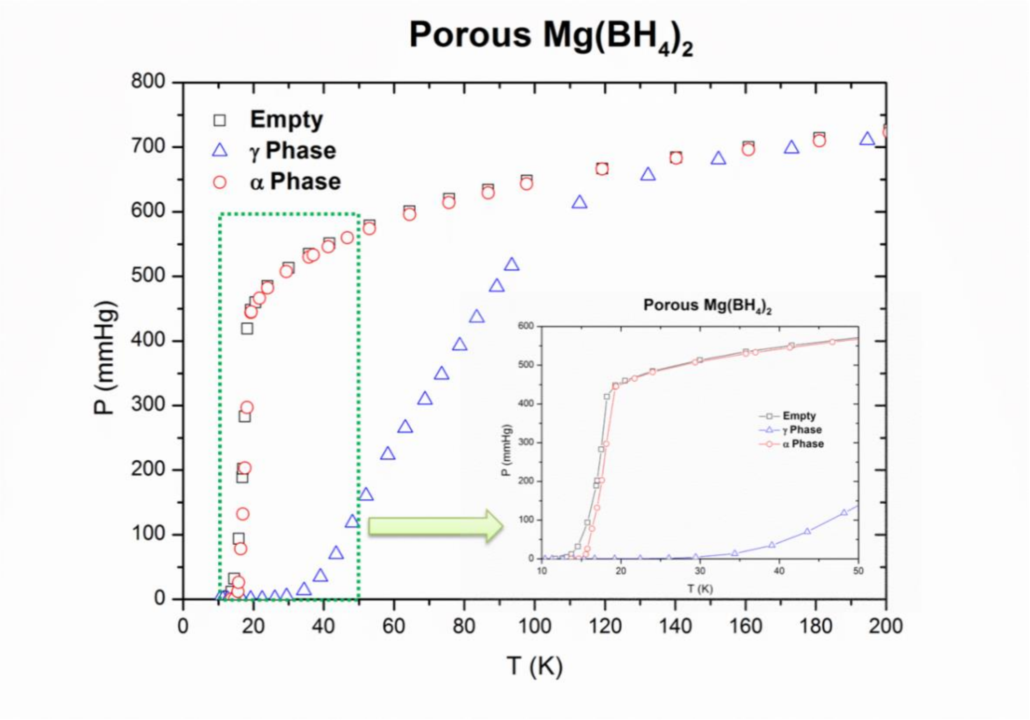

Fig. 13. Pressure change in the sample holder with temperature variation. Square: the empty sample holder, circle: sample holder containing α-$Mg(BH_4)_2$, triangle: sample holder containing γ–$Mg(BH_4)_2$.

Comparison of hydrogen loading density with metal-organic frameworks (MOFs).
Compared to hydrogen adsorption in MOFs, we discovered a new type of host-guest interaction of H_2 in γ–$Mg(BH_4)_2$, which may be used for the rational design of new porous materials or be expanded to utilisation for other gases. This host-guest interaction leads to a very high density of hydrogen molecules inside the pores. Importantly, one of the two H_2 sites (D22) has a particular orientation, i.e. not spherical but elliptical shape. In contrast, this high density has not been observed in metal-organic frameworks (MOFs) or covalent-organic frameworks (COFs), which are crystalline materials with a well-defined pore size distribution. So far very few detailed investigations of hydrogen adsorption in high-capacity MOF/COF-type materials at low temperatures, such as 20 K, have been conducted. Here are a few examples;
The metal-organic framework MIL-101 has a trimodal pore size distribution with pore openings of 7, 29, and 34 Å, but lacks a partly negatively charged inner surface as compared to γ-$Mg(BH_4)_2$. High-resolution hydrogen adsorption for MIL-101 measured at 19.5 K and pressures below 57 kPa reveal a successive filling of the pores with hydrogen. The BET surface area and micropore volume determined by hydrogen (19.5 K) and nitrogen adsorption (77 K) reveal similar results (*27*). Thus, no indication of any increased hydrogen density is observed for MIL-101, despite the confinement of hydrogen in the narrow pores of the MOF cage.
The metal-organic framework CPO-27-Co with large hexagonal one-dimensional channels, 10 Å in diameter, and N_2 BET surface area of 1012 m^2/g (*40*) resemble γ–$Mg(BH_4)_2$, except for the lack of a negatively charged inner surface. For Fe-MOF-74 or CPO-27-Fe, INS investigations show a shorter metal–adsorbate bond distance for sites with increased adsorption enthalpy. However, no shorter distance between the adsorbate molecules is observed (*41*).
A MOF possessing a bimodal pore size distribution and BET area similar to γ–$Mg(BH_4)_2$, is HKUST-1 or Cu-BTC, where the diameter of the small pore is about 5 Å. Neutron powder diffraction (measured at 5 K) after stepwise D_2 dosing at 77 K shows a successive filling of D_2 from small to large pores, however, no strongly increased hydrogen density has been observed (*42*). A similar filling of the two pores is obtained by detailed nitrogen adsorption experiments at 77 K showing a comparable uptake of hydrogen and nitrogen molecules (*43*).
In general, the density of the adsorbed hydrogen in MOFs is correlated with the N_2 BET area, having a value similar to that of a liquid monolayer. In framework structures that have open metal sites, physisorbed hydrogen may locally have a higher density (*44*). The example of γ-$Mg(BH_4)_2$, which has an exceptionally high hydrogen density in the (hydridic) pores, can thus be seen as unique.

Chapter 5. Inelastic Neutron Scattering - *In situ* study of H_2-loaded $Mg(^{11}BD_4)_2$ framework.

5.1 Quantum rotation of H_2

The quantum rigid rotor and the particle in a box are two of the most important applications of the Schrödinger equation. In the case of a diatomic molecule, the translational energy levels can be calculated using a simple model of a particle within a three-dimensional box, and rotational energy levels are given by $E_J = J(J+1)B_{rot}$, where B_{rot} is the rotational constant (7.35 meV for solid hydrogen) and $J = 0, 1, 2, \cdots$ is the rotational quantum number (Fig. 14) (*45*).

The hydrogen molecule is composed of two indistinguishable fermions that produce two different nuclear spin isomers and must preserve the overall wave function anti-symmetry. H_2 molecules with antiparallel nuclear spins can only exist with even rotational states ($J = 0, 2, \cdots, \text{even}$) parahydrogen (p-$H_2$) While molecules with parallel nuclear spins must have odd rotational states ($J = 1, 3, \cdots, \text{odd}$), orthohydrogen (o-$H_2$) Due to the zero-point energy, molecular hydrogen's behavior is dictated by quantum molecular dynamics, which is influenced by interaction potentials that may vary in a spatial dimension. Confined quantum particles entrapped in cage-like potentials provide an opportunity to probe the coupled translational-rotational states under model-like conditions (*46*).

INS spectra obtained from highly confined hydrogen using normal hydrogen (n-H_2) represent a convolution of o-H_2 and p-H_2 excitations, which complicates subsequent interpretation. Even at boiling temperature (~20 K), a mixture of o-H_2 and p-H_2 persists for long times due to the inefficiency of angular momentum exchange in the absence of a catalyst. Owing to the different cross-sections of o-H_2 and p-H_2 is possible to differentiate the vibrational from the rotational features of hydrogen. We thus followed the method of Ulivi et al (*47*). We performed two independent measurements using different ortho-para concentrations (one formed from n-H_2, and another using nearly pure p-H_2) to extract the pure component spectra by a linear combination (Fig. 16) and make unambiguous assignments.

The spectral intensity is given by:

$$S_{Total}(Q,\omega) \sim S_{Self_{CoM}}(Q,\omega) \otimes \sum_{JJ'} \delta(\omega - \omega_{JJ'}) \cdot v(J,J',Q)$$

where ω is the energy tranfer, Q is the momentum tranfer, $S_{Self_{CoM}}(Q,\omega)$ is the dynamical structure factor for the motion of the center of mass (also called Density of States DoS), the $\otimes$ symbol denotes a convolution product. The Dirac δ functions are centered at the energies of the rotational transitions, $\omega_{JJ'}$ of the H_2 molecule. The intensity factor $v(J,J',Q)$ is function of the momentum transfer Q and depends on the rotational transition $J \rightarrow J'$ of the molecule.

INS is not subject to selection rules. Therefore all transitions are allowed. At low temperatures, only the lowest rotational levels are populated, $J = 0, 1$. The intensity factors are calculated using the rigid rotor approximation (*48*). The transitions contributing to the overall intensity are, for o-H_2, the rotational elastic $J_{1\rightarrow1}$ and the inelastic $J_{1\rightarrow2}$, while for p-H_2 only the $J_{0\rightarrow1}$has an appreciable contribution, the $J_{0\rightarrow0}$ is weighted by the coherent cross section of hydrogen and can be neglected. In practice, this means that for n-H_2, the intensity below 12 meV corresponds to the rattling transitions of the oH_2 molecules (Fig. 16 left). In the case of highly enriched p-H_2, there is no appreciable intensity below 13 meV. The intensity above the rotational features (red and blue peaks in Fig. 16) is the convolution of the rotational transitions with the vibrational tranistions.

In the case of n-H_2 all transitions have intensity. Table 8 shows the observed transitions in the spectrum and the experimental positions of the peaks (the resolution of VISION is $\Delta\omega/\omega = 2\%$. It can be seen that all observed transitions are compatible with a value of $B_{rot} = 7.22$ meV.
The presence of higher order rotational features in the spectra is highly unusual, it is a consequence of the trapping of the hydrogen molecule inside the cavity and a strong reduction of the Debye-Waler factor due to such confinement.

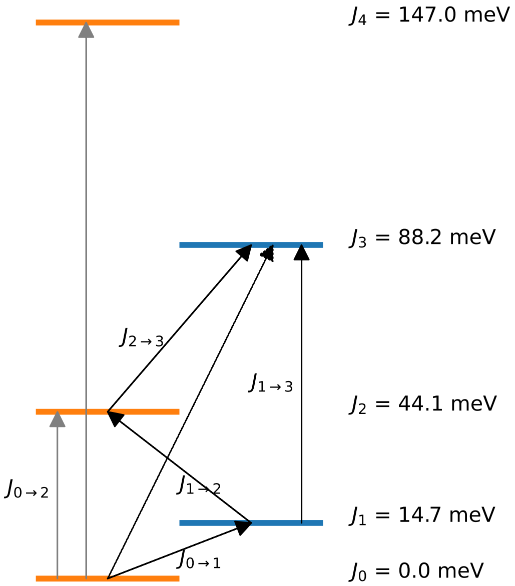

Fig. 14. Scheme of rotational energy transitions of H_2. The transitions in black are observable transitions while the grey arrows indicate a non-observable transition.

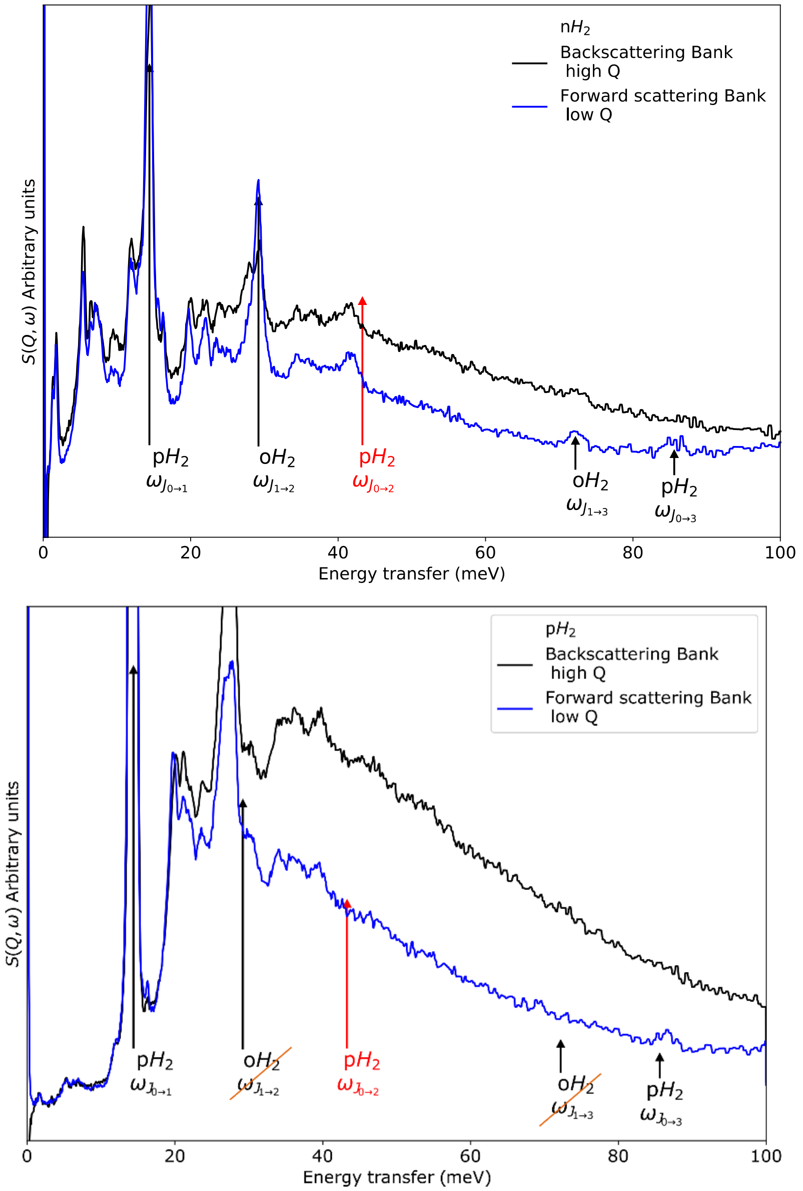

Fig. 15. INS spectra of n-H_2 **(upper)** and p-H_2 **(bottom)** in $Mg(BD_4)_2$ for back and forward scattering. The arrows indicate the rotational transitions from para and ortho hydrogen. For nH_2, we observe the high energy transitions $J_{1\to3}$ and $J_{0\to3}$ that are not seen in most cases due to the high momentum transfer in instrument VISION. For pH_2, the transitions $J_{1\to3}$ and $J_{1\to2}$ are not observable because they start from the ortho state J_1. The transition $J_{0\to2}$ is not observed as it has a negligible cross section (*49*) (see Table 8).

Table 8. Rotational energy transitions corresponding to the B_{rot} = 7.22 meV. Experimental data see Fig. 3 in the main text of the article. Uncertainties are 1 standard deviation.

$B_{rot} = 7.22$ meV	ω_{rot}	Exp		ω_{rot}	Exp		ω_{rot}	Exp
$J_{0\to1}$	14.4	14.4 ± 0.2	$J_{0\to2}$	43.3	-----	$J_{0\to3}$	86.6	85.6 ± 1.7
$J_{1\to2}$	28.9	29.2 ± 0.4	$J_{1\to3}$	72.2	72.3 ± 0.7	$J_{1\to4}$	130.0	-----

5.2 INS roto-vibrational analysis

5.2.1 Para and normal hydrogen in γ–$Mg(BD_4)_2$

Fig. 16 (left) shows the INS spectra of γ–$Mg(BD_4)_2$ with a load of 2.8 mmol of n-H_2 (black trace), n-H_2 is around the statistical mixture at room temperature (75% o-H_2 and 25% p-H_2). The area shown in gray is the signal of orthohydrogen and it is proportional to the vibrational transitions σ_{vib} of the hydrogen molecule. The vibrational transitions are given by low-energy phonons that correspond to the density of states (DoS) of the hydrogen molecule. The o-H_2 spectrum (gray shaded area) show five well-defined phonon transitions located at 1.8 meV (a), 5.5 meV (b), 6.4 meV (c), 7.2 meV (d) and 11.9 meV (e). The peaks that are shown in red and blue are the peaks of parahydrogen rotational transitions $\sigma_{rot_{J(0\rightarrow1)}}$ with a cross-section of ~2x80 barn. The intensity observed in the data above 15 meV are the overtones of vibrational phonon modes (DoS) of the o-H_2 given by its combination with the rotational transitions $\sigma_{rot+vib} \sim \sigma_{rot_{J(0\rightarrow1)}} \otimes \sigma_{vib}$.

Fig. 16 (right) shows two rotational peaks that correspond to the transition ($J_{0\rightarrow1}$) located at 13.9 meV ((f) perturbed - red) and 14.6 meV ((g) unperturbed - blue). We used a Gaussian fitting for both rotor peaks. The analysis determines the area of each peak in arbitrary units, for the unperturbed (39.56) and the perturbed (14.26). The ratio of the areas that correspond to the population is 2.77 for the maximum load, Table 9 has all the relevant information for the peak fitting at maximum coverage.

The spectral intensity for molecular hydrogen for a roto-vibrational transition is given by $\sigma_{rot+vib} \sim \sigma_{rot} \otimes \sigma_{vib}$. For the case of pH_2, the cross-section of the transition $J_{0\rightarrow0}$ is very small ($\sigma_{rot_{J(0\rightarrow0)}} \sim 0$ barn) (*49*). Hence, the vibrational model of the pH_2 are not well-visible below the rotational transition. In contrast, for oH_2, the cross-sectional area of the vibrational modes is given by $\sigma_{vib} \sim 2 \times 80$ barn. This difference in cross-sectional area readily visible when comparing the spectra of n-H_2 and p-H_2 (see Fig. 16 left – grey area).

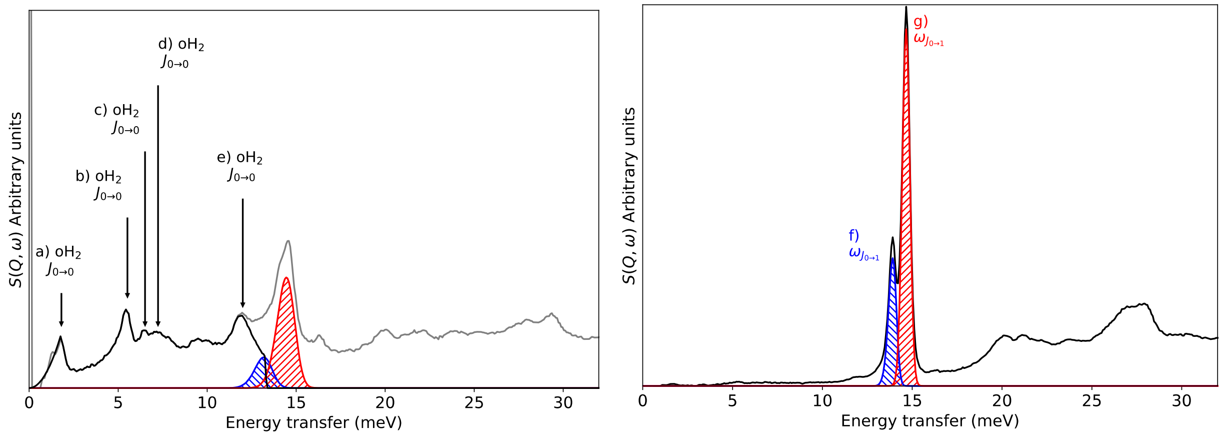

Fig. 16. INS spectra of hydrogen in $Mg(BD_4)_2$. **Left**, spectrum of 16.8 mmol/g of n-H_2 ($Mg(BD_4)_2 \cdot 1.04H_2$), **right**, the spectrum of 23.5 mmol/g of p-H_2 ($Mg(BD_4)_2 \cdot 1.46H_2$). The peaks in red represent the rotational transition of $J_{0\to1}$, centered around 14.6 meV, while the blue peak is a $J_{0\to1}$ centered around 13.9 meV. The greyed area in the left panel is the extracted vibrational density of states of the hydrogen molecule.

Table 9. Gaussian analysis parameters of the rotor transitions ($J_{0\to1}$).

	**Peak**	**Center [meV]**	**Area [a.u.]**	**FWHM**	$\boldsymbol{\sigma}$	$\boldsymbol{\gamma}$
***n-H₂* (16.8 mmol/g)**	*perturbed* (f)	13.20	4.05	1.18	0.654	-1.33
	unperturbed (g)	14.45	14.56	1.14	0.654	-1.33
***p-H₂* (23.5 mmol/g)**	*perturbed* (f)	13.90	15.20	0.50	0.264	-1.02
	unperturbed (g)	14.65	40.90	0.50	0.264	-1.02

5.2.2 INS as function of p-H_2 loading and temperature

Fig. 17 show the INS spectra as function of p-H_2 loading collected at 5 K. At the lowest loading (5.88 mmol/g), the spectra show an overall higher resolution allowing us to clearly observe peaks at 1.6 meV, 16.3 meV and 22.0 meV that overlap or are overwhelmed at higher loadings. At lower concentrations, the vibrational transitions are better defined. A peak at 27.8 meV is well defined and doesn't overlap other peaks. The overall intensity of the spectra increases with increasing p-H_2 loading and with all the features remaining similar at similar energies for all dosing points. Table 10 show the results of the Gaussian analysis of the rotational peaks at 13.9 and 14.6 meV.

Fig. 18 show the INS spectra of the highest loading of p-H_2 (23.5 mmol/g) ($Mg(BD_4)_2 \cdot 1.46H_2$) as function of temperature (5 – 215 K). The peak at 13.9 meV and 27.8 meV disappear simultaneously when the temperature reaches 95 K. The peak at 14.7 meV is clearly visible up to 155 K and some intensity remain even at 185 K.

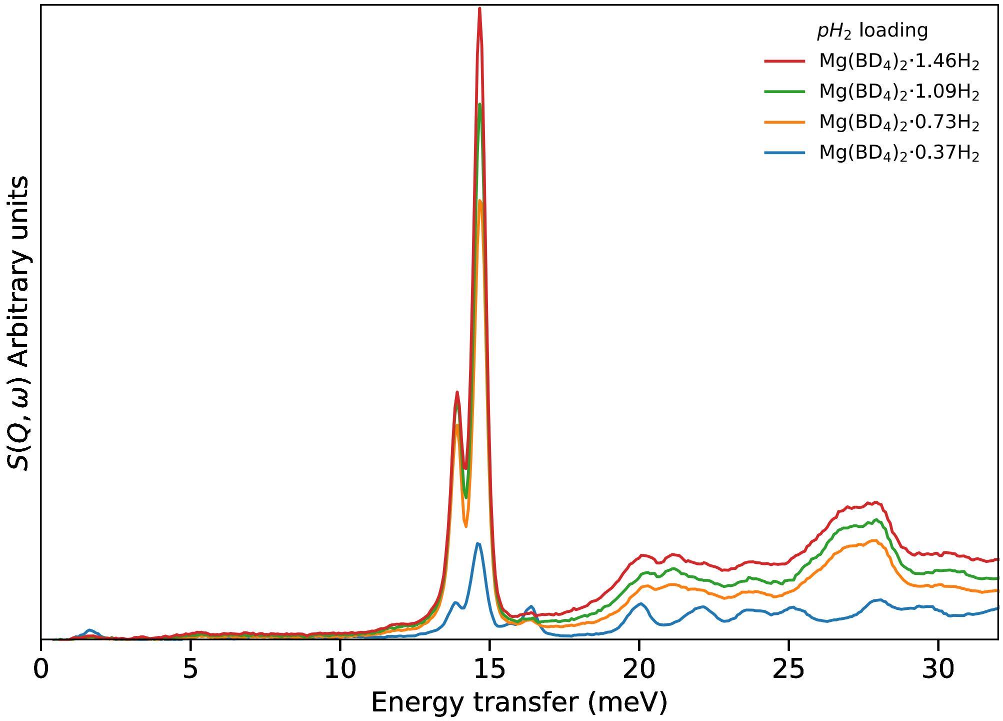

Fig. 17. INS spectra of $Mg(BD_4)_2$ for different p-H_2 loading collected at 5 K. The spectra show an increasing uptake with loading, and similar features at all energies.

Table 10. Fitting parameters for the rotational features of p-H_2 as a function of coverage. Peak 1 is "perturbed rotor" while peak 2 is standard pH_2 $J_{0\to1}$ rotor transition. The loading by H_2 per Mg atom is not corrected for the fraction of the amorphous $Mg(BD_4)_2$ phase, estimated to represent ~30%, see the main text.

Loading nominal (mmol)	Loading (mmol/g)	H_2 per Mg atom	Peak 1 center (meV)	Peak 2 center (meV)	Ratio peak areas	Area Peak1+ Peak2 (normalized)	Area spectra (normalized)
1.0	5.88	0.37	13.82	14.63	2.85	0.180	0.305
2.0	11.8	0.73	13.89	14.69	2.19	0.715	0.656
3.0	17.6	1.09	13.89	14.68	2.34	0.860	0.816
4.0	23.5	1.46	13.90	14.67	2.69	1	1

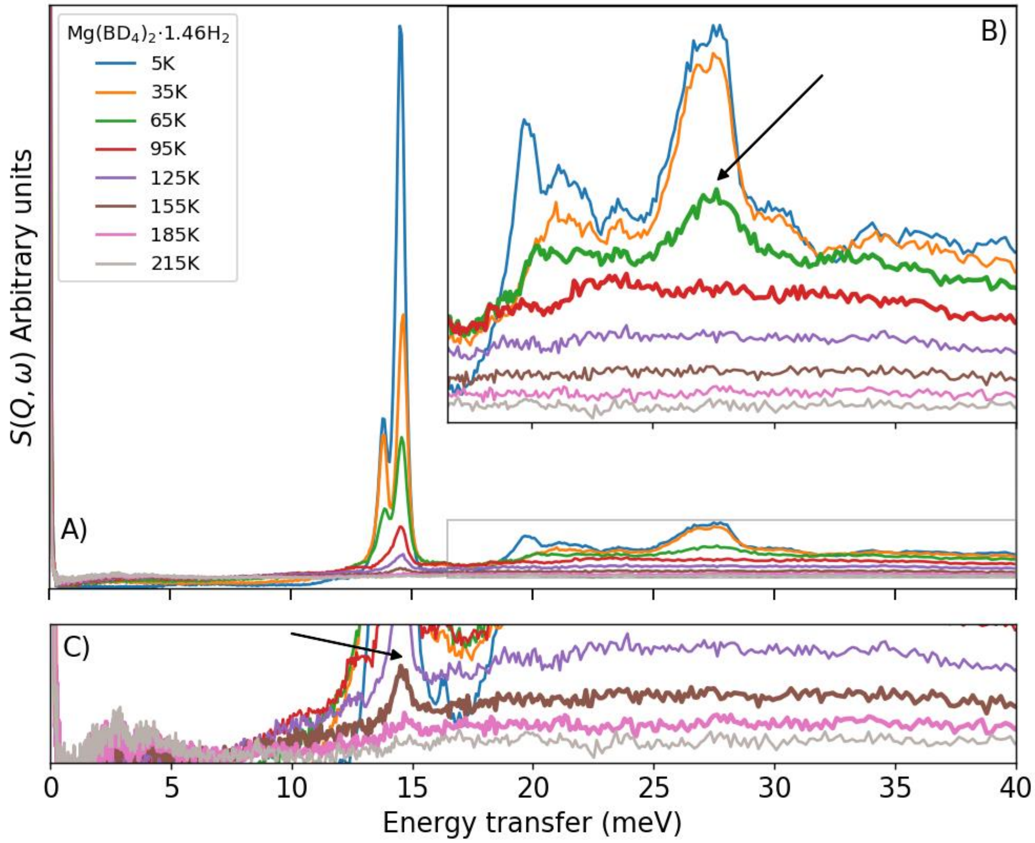

Fig. 18. A) INS spectra of 23.5 mmol/g of p-H_2 ($Mg(BD_4)_2 \cdot 1.46H_2$) collected at different temperatures. B) Zoom view of the peak around 17 meV, the peak at 27.8 meV disappears when temperature reaches 95 K. C) Zoom view for higher temperatures. The peak at 14.7 meV is observable up to 185 K.

5.3. Simultaneous quantum rotational excitation (SQRE)

To fully account for all the intensity distribution of the pH_2 (Fig. 19) spectra, we note that there is a peak at 27.8 meV that has an energy that is twice the energy of the "perturbed rotor" peak (blue hatched area). If we have almost pure pH_2, there is no rotational transition that has such energy transfer (see Table 8). In the case of p-H_2 under pressure (Supplementary section 5.5) there is a peak that appears at an energy twice that of the rotational line when the pressure increases. This peak is due to a process that involves "one neutron" simultaneously exciting two rotational transitions on two neighboring molecules or a *simultaneous quantum rotational excitation* (SQRE). This should not be confused with multiple scattering. This a single quantum event that constitutes evidence of the strong coupling between these hydrogen molecules due to pressure.

Fig. 19 (red and blue area) shows the result of the convolution of the different rotor peaks. These peaks are calculated by convoluting the perturbed rotor (13.9 meV - blue) and unperturbed rotor (14.6 meV - red) with themselves, respectively. These convolutions produce the blue (27.8meV) and red (29.2 meV) modes, respectively. Of these peaks, only the one at 27.8 meV is observed for the experiment using p-H_2.

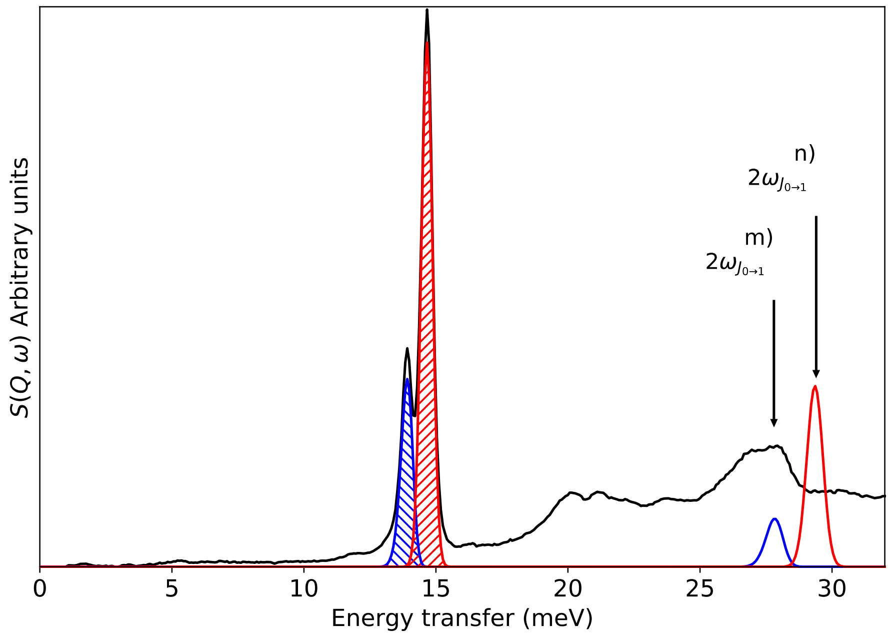

Fig. 19. INS spectra of $Mg(BD_4)_2$ with a load of 23.5 mmol/g of p-H_2 (black). Two additional peaks are marked in blue and red located at 27.8 and 29.2 meV corresponding to the convolution of each peak with itself.

5.4. Calculation of overtone vibrational modes and SQRE

The higher energy part of the INS spectra (16 < E < 30 meV) above the rotational peak has several peaks that compose the overtones. These overtones are the combination of the vibrational modes (DoS) with the rotational peaks. Fig. 20 shows the product of convoluting the o-H_2 DoS (purple upper area) with the p-H_2 rotational lines (blue + red hatched areas). The resulting overtone curve (dark grey) is composed by the peaks located at (h) 16.3 meV, (i) 20.1 meV, (j) 21.3 meV, (k) 23.6 meV, and (l) 26.6 meV, which correspond to the overtones of the phonon modes (a,b,c,d,e), respectively. Second order overtones (light grey area) are calculated by convoluting the overtones (dark grey area) with the o-H_2 DoS (purple upper area).

The experimental data show another peak located exactly at 27.8 meV, this peak correspond to the SRQE (blue area) of the 'perturbed' rotor (see Fig. 19). The sum of the first and second order overtones (dark yellow) plus the calculated SQRE (blue area) result in a curve (green) that reproduce the experimental p-H_2 INS spectra. The experiment (black) shows no signal located at 29.2 meV that would correspond to the SQRE of the 'unperturbed' rotor, that would correspond to the peak shown in red.

Only the SQRE at 27.8 meV is observable while the other at 29.2 meV is not, indicating that the rotor peaks are not coupled. This suggest that there are two independent environments for the hydrogen molecule. The ratio of the areas the rotors $J_{0\to1}$ is 2.7 (see Table 3), which is a direct measurement of the ratio of hydrogen populating each environment.

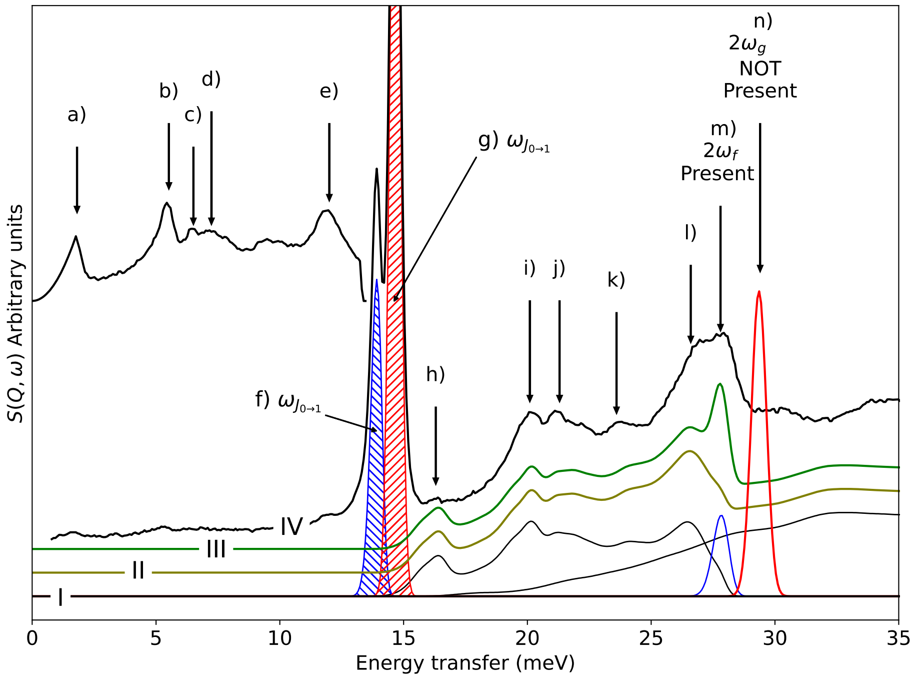

Fig. 20. INS spectra of $Mg(BD_4)_2$ with a load of 23.5 mmol/g of p-H_2 (black). The vibrational modes DoS [a-e] (grey upper area) are convoluted with the rotor transitions [f,g] to obtain the overtones [h-l] (16-28 meV grey area). The sum of the overtones and the bifold rotor [m] (green line) reproduces the experiment (E > 16 meV). The blue peak m) in necessary to account for the intensity. The peak n) does not exist in the data. This is because peak f) and g) are distinct p-H_2 molecules. Note that in the case of p-H_2 there are no rotational transitions that can account for peak m).

5.5. Comparison with H_2 at high-pressure

As a means of comparison, we present additional INS experiments of p-H_2 and n-H_2 at high-pressure. The experiments were measured using VISION spectrometer at the Spallation Neutron Source (SNS) at Oak Ridge National Laboratory (ORNL) in Tennessee, USA. A high-pressure dosing device was used coupled with CuBe high-pressure cell at 5K to reach pressures up to 4000 bar.

Fig. 21 show the INS spectra of solid p-H_2 at 200 bar and 2500 bar. In both cases, there is a peak near 14.6 meV that corresponds to the rotation $J_{0\rightarrow1}$, and overtones $\omega_{J_{0\rightarrow1}} \otimes \omega_{vib}$ are located between 16 meV and 28 meV. The overtone modes located between 16 meV and 29 meV shift to higher energies with increasing pressure. Interestingly, at the higher pressure a SQRE is observed near 29.0 meV, which corresponds to the convolution of the rotor peak with itself. Because the experiment was performed using p-H_2, the rotational transition ($J_{1\rightarrow2}$) that should be located at 29.2 meV is not observed as the J=1 state is not populated because it corresponds to an ortho-spin-state.

Fig. 22 a comparison of the experiment using p-H_2 in $Mg(BD_4)_2$. The high-pressure experiment was scaled (y-axis) and translated (x-axis) so the rotational peak matches the high-pressure peak a 13.2 meV (red) and the unperturbed peak at 14.6 meV (blue) (see Fig. 16 right). Hence, the SQRE (Fig. 21) energy is shifted the same as the rotor, showing that the corresponding SQRE of the "perturbed" peak (m) is observable while the SQRE of the "unperturbed" (n) is not visible in the spectra.

Fig. 23 shows the INS spectra of solid n-H_2 for two high-pressure points (~0 bar and 2400 bar), the spectra show the rotational transition ($J_{0\rightarrow1}$) shifting to lower energies with increasing pressure. The shift to lower energies of the rotor is due to the quadrupole interaction of o-H_2. A vibrational mode (rattling) is located at lower energies (5 meV to 13 meV) and moves towards higher energies with increasing pressure. The overtones of the vibrational modes are located between 15 and 29 meV, and shift to higher energies with increasing pressure. Contrary to the p-H_2 high-pressure experiment, the peak located at 29.2 meV is the ortho rotation ($J_{1\rightarrow2}$) (green-line), and a SQRE ($2\omega_{J_{0\rightarrow1}}$) is not well-defined.

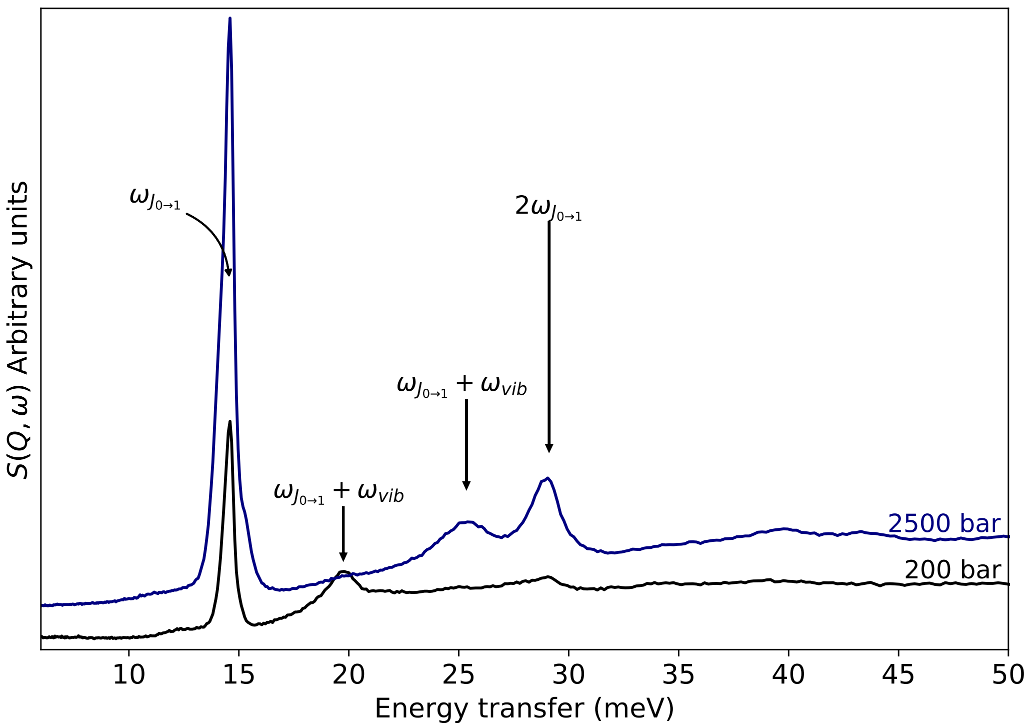

Fig. 21. INS spectra of solid p-H_2 at 200 (black) and 2500 bar (blue). The peak near 14.6 meV correspond to the rotation $J_{0\to1}$ and its intensity increases with pressure while its energy is reduced. At higher pressure, SQRE is located at 29.0 meV.

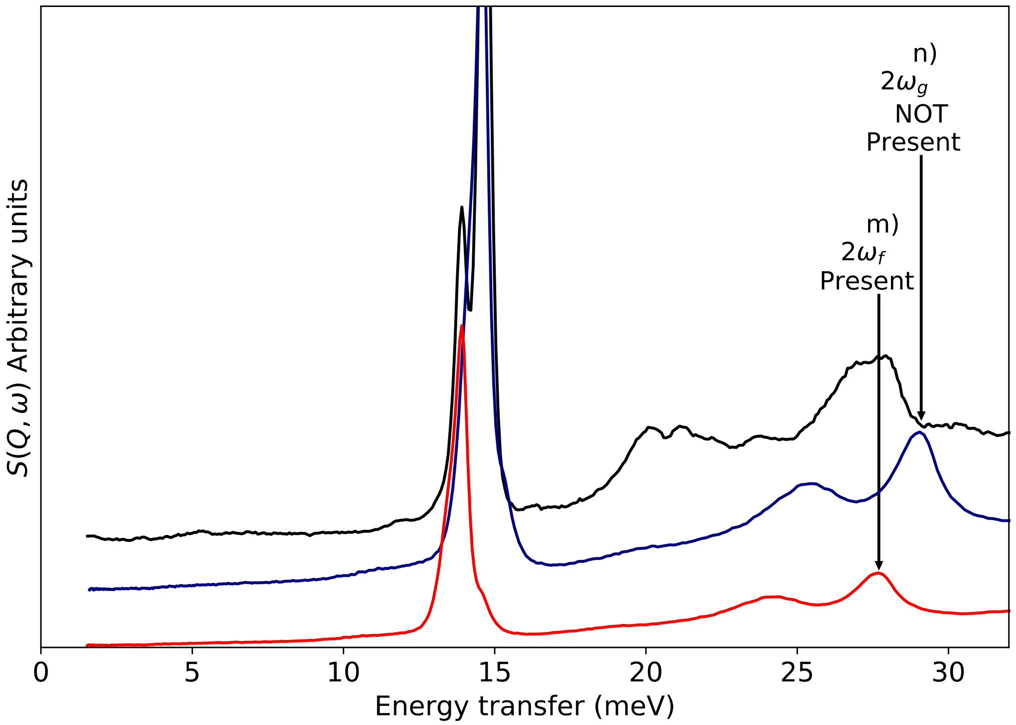

Fig. 22. INS spectra $Mg(BD_4)_2$ with a load of 23.5 mmol/g of p-H_2 (black). To compared intensities and energies the INS spectrum of para-hydrogen, p-H_2, at $p(H_2)$ = 2500 bar is scaled (x- and y-wise) to correspond to both rotor intensities (13.9 meV (red) and 14.6 meV (blue)).

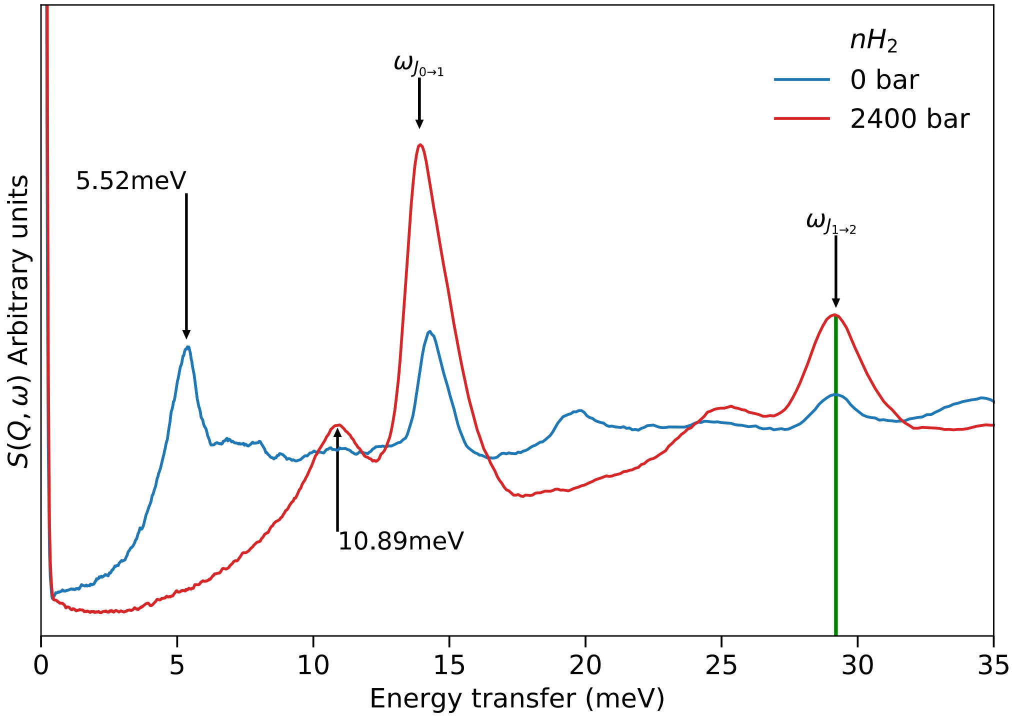

Fig. 23. INS spectra of solid n-H_2 at high-pressure collected at 5 K. The INS spectra near 0 bar and at 2400 bar show a mode that is located at 5.52 meV and 10.89 meV, respectively. The peaks located near 14.0 meV and at 29.2 meV correspond to rotational transitions $J_{0\to1}$ and $J_{1\to2}$, respectively.

5.6 Density of hydrogen confined in $Mg(BD_4)_2$

By looking at the effect of pressure on highly enriched pH_2 and nH_2 (see Supplementary section 5.5) on the phonon spectrum of hydrogen; we can calibrate a "representative density" required for the effect of pressure to be observable.

Fig. 24 upper left show a linear interpolation of the phonon energies of n-H_2 as a function of the pressure (orange line). The phonon energies (blue triangles) were taken from the full experiment of solid n-H_2 (0 bar to 4000 bar) (Fig. 23).

The different vibrational modes observed in the DoS (Fig. 16 left) are shown as green points correspond to a vibration in the three directions. The highest energy is 11.9 meV represents the higher vibration mode that correspond to a high-pressure environment of 2915 bar (Fig. 24 green circle).

The density of bulk hydrogen has been previously reported as function of pressure by Silvera et al. (*50*) (Fig 24 upper right). Assuming a pressure of 2915 bar (red- dotted), the corresponding density is 132 g/L. A value much higher than bulk-liquid (70.9 g/L) and -solid (86.7 g/L) density at 1 bar. This value is 1.51 times higher than the bulk-solid density at 1 bar.

The cross-sectional area of the H_2 molecule as function of pressure (Fig. S24 bottom left) was calculated assuming a hexagonal close packing and is given by:

$$\sigma = f \cdot \left({m}/{\rho N_A} \right)$$

where $f = {\sqrt{3}}/{\sqrt[3]{4}} = 1.091$ is the hexagonal closed-packing factor, *m* is the molar mass, ρ is the volumetric density and N_A is the Avogadro number (Rouquerol, Rouquerol et al. 1999, Lowell, Shields et al. 2004). The corresponding cross-sectional area for 2915 bar is 9.40 Å^2.

The H_2-H_2 intermolecular distance as function of pressure (Fig. S24 bottom right) is given by the relation:

$$\sigma = \frac{\sqrt{3}}{2} \cdot r^2$$

where σ is the cross-sectional area of H_2 and r is the intermolecular distance (*51*). The corresponding intermolecular distance for a pressure of 2915 bar is 3.29 Å.

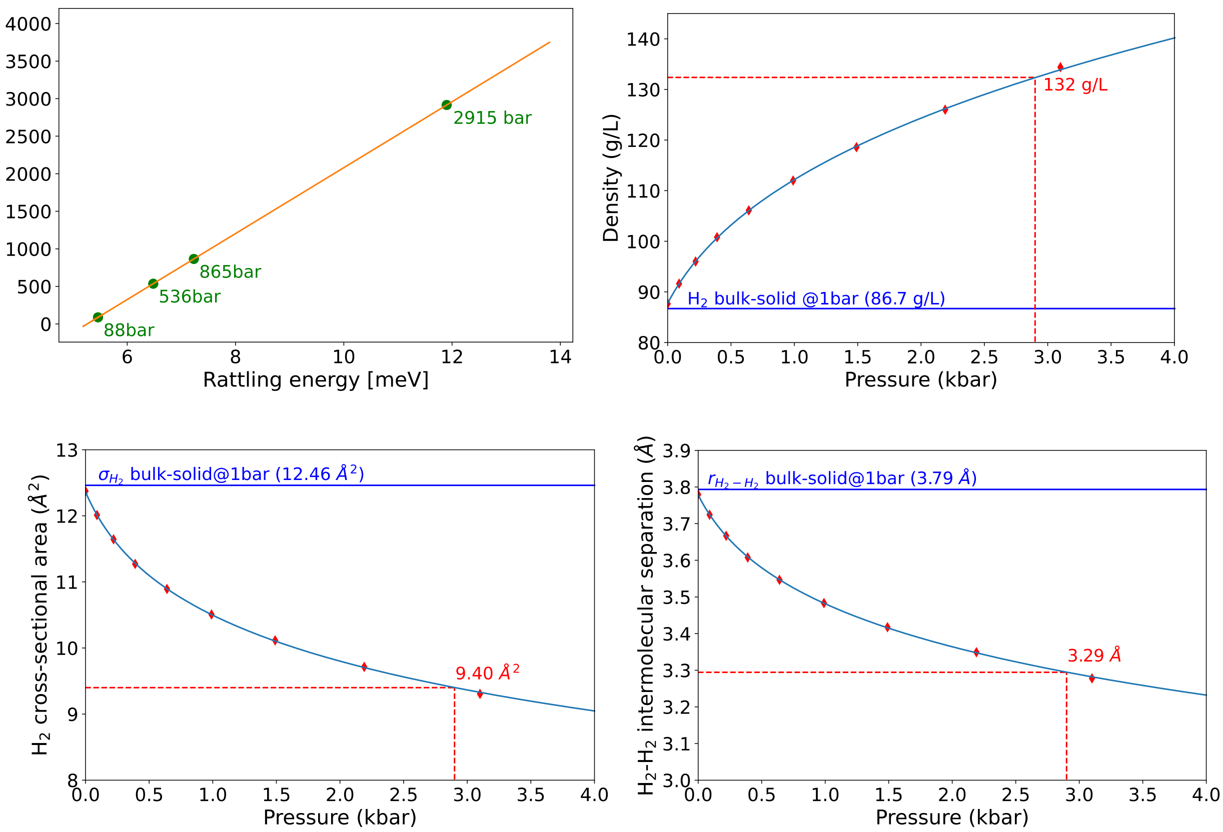

Fig. 24. Upper left, linear interpolation of the phonon energies of n-H_2 as a function of the pressure. The energies observed in the phonon modes [b-e] in Supplementary Fig. 2 are shown as green points. The phonon mode at 11.9 meV [e] corresponds to a pressure environment of 2915 bar. Upper right, hydrogen density as a function of pressure Bottom left, corresponding cross-sectional area of hydrogen as a function of the pressure. Bottom right, H_2-H_2 intermolecular separation as function of pressure. The corresponding separation for a phonon mode of 11.9 meV (Fig. 2 peak [e]) is 3.29 Å.

Chapter 6. DFT simulations

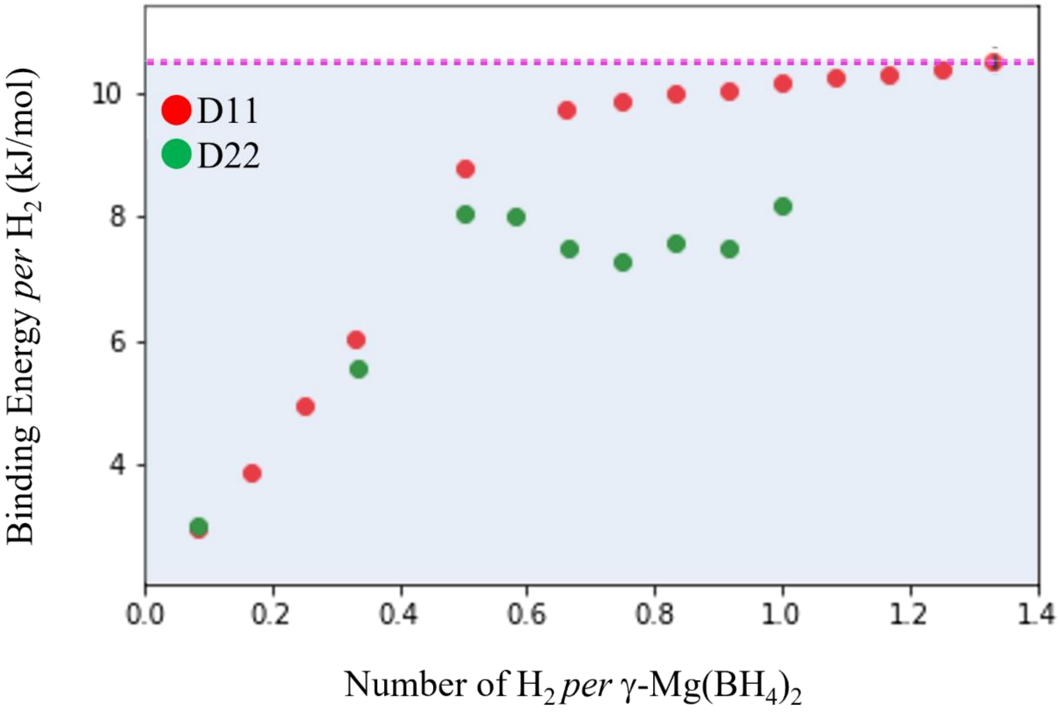

Fig. 25. Calculated binding energy *per* H_2 molecule adsorbed in γ-$Mg(BH_4)_2$. The binding energy is defined as the negative of γ-$Mg(BH_4)_2 + x\ H_2 \rightarrow$ γ-$Mg(BH_4)_2 \cdot xH_2$. This calculation uses sequential filling of the D11 site (red circle) until fully occupied (1.33 H_2/Mg), and the D22 site (green circle) until fully occupied. The D22 site can accommodate a maximum of 1.0 H_2/Mg. Dotted magenta line represents the most favored binding energy (fully loaded D11 site).

Explanation of thermodynamic analysis on Figures 25, 28 and manuscript Figure 6a,b

Our DFT calculations start with a model of γ-$Mg(BH_4)_2$ on which we adsorb H_2 molecules on different sites (D11 and D22) at different loadings (amount of H_2). For each these configurations, we compute the DFT total energy and compare it to the energy of γ-$Mg(BH_4)_2$ and H_2 obtaining reaction energies such as γ-$Mg(BH_4)_2$ + x H_2 → γ-$Mg(BH_4)_2$·xH_2 as plotted in Figure S25. While S25 is informative, it does not provide the full picture of stability of the system. If one considers for instance the loading of 1/12 H_2 per γ-$Mg(BH_4)_2$ (there are 12 Mg sites in the unit cell), we can obtain the reaction energy γ-$Mg(BH_4)_2$ + 1/12 H_2 → γ-$Mg(BH_4)_2$·1/12 H_2 but there might be other competing reactions such as γ-$Mg(BH_4)_2$ + 1/12 H_2 → 1/16 γ-$Mg(BH_4)_2$·1.33H_2 + 15/16 γ-$Mg(BH_4)_2$. In fact, for our system, the second reaction is favoured and the segregation into γ-$Mg(BH_4)_2$·1.33H_2 and γ-$Mg(BH_4)_2$ is energetically preferred. To take into account *all* decomposition paths, *all* reaction energies between configurations of H_2 in γ-$Mg(BH_4)_2$, we can rely on the often-used convex hull construction where we plot the total energy of all our H_2 configurations in the host versus two reference states, for instance γ-$Mg(BH_4)_2$·1.33H_2 and γ-$Mg(BH_4)_2$. By constructing the convex envelope connecting all these energies (the convex hull), we can directly see what phases will be the most stable (see Methods). For Figure 6a, we constructed the convex hull and found that any configuration of H_2 in γ-$Mg(BH_4)_2$ (up to 1.33 per γ-$Mg(BH_4)_2$) will energetically prefer to form the fully D11-loaded γ-$Mg(BH_4)_2$·1.33 H_2. This is the behaviour of what is called in materials thermodynamics a two-phase system (versus a solid solution). We note that the reference state chosen will not alter the conclusion on phase stability.A similar explanation can be used for Figure 28 and Figure 6b where binding energy and convex hull constructions are performed for the higher loadings of H_2. The convex hull indicates that over the full range of H_2 loading, three phases are stable: γ-$Mg(BH_4)_2$, γ-$Mg(BH_4)_2$.1.33H_2 (D11 fully loaded) and γ-$Mg(BH_4)_2$.2.33H_2.

We note that our model assumes to be at zero K as it does not take into account entropic factors.

Structure of γ-$Mg(BH_4)_2$ **with fully loaded D11 site:**

```
Full Formula (Mg12 B24 H96 H32)
abc   :  13.504136  13.504600  13.504708
angles: 109.442302 109.534838 109.458340
Sites (164)
  #  SP          a          b          c
---  ----  --------  --------  --------
  0  Mg    0.24999   0.625196  0.375201
  1  Mg    0.749971  0.374786  0.624786
  2  Mg    0.249968  0.124999  0.875009
  3  Mg    0.749994  0.874987  0.12498
  4  Mg    0.124764  0.37499   0.750006
  5  Mg    0.875208  0.625007  0.249983
  6  Mg    0.124875  0.249804  0.374906
  7  Mg    0.875082  0.750181  0.625082
```

8	Mg	0.375199	0.125194	0.250202
9	Mg	0.624759	0.874789	0.74979
10	Mg	0.375091	0.749978	0.124915
11	Mg	0.624877	0.250009	0.875082
12	B	0.063647	0.627199	0.31363
13	B	0.93632	0.372798	0.686354
14	B	0.563587	0.250011	0.436471
15	B	0.436377	0.749972	0.563512
16	B	0.186427	0.250031	0.813547
17	B	0.813566	0.749976	0.186456
18	B	0.313589	0.127132	0.063623
19	B	0.686381	0.872852	0.936368
20	B	0.127204	0.31367	0.563647
21	B	0.872783	0.686337	0.436356
22	B	0.186412	0.436391	0.372835
23	B	0.81356	0.563591	0.627148
24	B	0.372778	0.9364	0.186434
25	B	0.6272	0.063589	0.813572
26	B	0.936338	0.063497	0.249991
27	B	0.063617	0.936494	0.74999
28	B	0.749969	0.436478	0.063555
29	B	0.250009	0.563514	0.936443
30	B	0.313526	0.686416	0.249966
31	B	0.686434	0.313563	0.750019
32	B	0.249974	0.186408	0.313511
33	B	0.749983	0.813574	0.686475
34	B	0.436296	0.186301	0.872789
35	B	0.563668	0.813683	0.127206
36	H	0.418141	0.194252	0.148729
37	H	0.581827	0.805731	0.851261
38	H	0.081871	0.23053	0.7761000
39	H	0.918125	0.769485	0.223906
40	H	0.545611	0.269562	0.351458
41	H	0.454351	0.730423	0.648525
42	H	0.045678	0.694251	0.276124
43	H	0.954293	0.305744	0.723858
44	H	0.305745	0.851324	0.081913
45	H	0.69423	0.148668	0.918091
46	H	0.351259	0.081778	0.805708
47	H	0.648708	0.918209	0.194288
48	H	0.194239	0.276206	0.54565
49	H	0.805741	0.723797	0.454347
50	H	0.276087	0.581919	0.230517
51	H	0.723867	0.41806	0.769465
52	H	0.269472	0.22387	0.418035
53	H	0.730486	0.776115	0.581951
54	H	0.851362	0.045591	0.269524
55	H	0.148594	0.954402	0.730456
56	H	0.769461	0.351459	0.045595
57	H	0.230519	0.648533	0.954402
58	H	0.223802	0.454371	0.305757
59	H	0.776171	0.545609	0.694228
60	H	0.081897	0.351272	0.305763
61	H	0.918074	0.64871	0.694218
62	H	0.418044	0.723865	0.269421
63	H	0.581916	0.276115	0.730569
64	H	0.954324	0.14849	0.230447
65	H	0.045635	0.851502	0.769534
66	H	0.454269	0.223797	0.805757
67	H	0.545698	0.776188	0.194238
68	H	0.194264	0.418176	0.648707
69	H	0.805714	0.581834	0.351287
70	H	0.148686	0.694287	0.418205
71	H	0.851282	0.305707	0.581778
72	H	0.305695	0.954341	0.223837
73	H	0.694277	0.045648	0.776164
74	H	0.223901	0.269495	0.918092
75	H	0.776092	0.730515	0.081909
76	H	0.230466	0.081898	0.276087
77	H	0.769492	0.918085	0.723899

78	H	0.648571	0.230436	0.454386
79	H	0.351392	0.769545	0.5456
80	H	0.730489	0.454401	0.148561
81	H	0.26949	0.54559	0.851437
82	H	0.276149	0.194213	0.045608
83	H	0.723813	0.805765	0.954377
84	H	0.318657	0.060493	0.980272
85	H	0.681306	0.939484	0.019714
86	H	0.181336	0.161602	0.741827
87	H	0.818644	0.838397	0.258171
88	H	0.580293	0.338458	0.519798
89	H	0.419675	0.661527	0.480187
90	H	0.080262	0.56053	0.241842
91	H	0.919697	0.439456	0.758142
92	H	0.439401	0.019739	0.181341
93	H	0.560569	0.980252	0.818662
94	H	0.480235	0.818833	0.060555
95	H	0.51973	0.181155	0.939445
96	H	0.060536	0.241892	0.580278
97	H	0.93945	0.758118	0.419727
98	H	0.241715	0.681232	0.161341
99	H	0.758248	0.318753	0.838647
100	H	0.338443	0.258165	0.318688
101	H	0.661515	0.741818	0.681299
102	H	0.019772	0.080266	0.338579
103	H	0.980182	0.919724	0.661402
104	H	0.838445	0.519773	0.080264
105	H	0.161525	0.480216	0.919726
106	H	0.258267	0.41966	0.439517
107	H	0.741701	0.580323	0.560471
108	H	0.181296	0.519689	0.439437
109	H	0.818671	0.480291	0.56054
110	H	0.318659	0.758148	0.338387
111	H	0.681298	0.241831	0.661597
112	H	0.080318	0.019792	0.838435
113	H	0.919638	0.980199	0.161548
114	H	0.580286	0.741901	0.060566
115	H	0.419678	0.258084	0.939429
116	H	0.060526	0.318785	0.480189
117	H	0.939445	0.681209	0.51981
118	H	0.019675	0.439458	0.681273
119	H	0.980291	0.560545	0.318714
120	H	0.560489	0.080296	0.741723
121	H	0.439489	0.919697	0.258283
122	H	0.258161	0.338496	0.81865
123	H	0.741833	0.661508	0.181345
124	H	0.161347	0.1812	0.241697
125	H	0.838613	0.818786	0.75829
126	H	0.480234	0.161567	0.41978
127	H	0.519731	0.838425	0.580212
128	H	0.338629	0.580312	0.019866
129	H	0.661342	0.419668	0.980131
130	H	0.241803	0.060471	0.080279
131	H	0.758164	0.939513	0.919709
132	D11	0.101416	0.622912	0.606982
133	D11	0.836347	0.358235	0.345698
134	D11	0.335344	0.986151	0.476594
135	D11	0.600858	0.991766	0.477251
136	D11	0.525742	0.509644	0.15862
137	D11	0.475059	0.490762	0.84197
138	D11	0.974695	0.129203	0.982235
139	D11	0.966906	0.862508	0.974616
140	D11	0.599411	0.123516	0.108592
141	D11	0.338478	0.857516	0.845664
142	D11	0.099933	0.988948	0.476532
143	D11	0.836303	0.988892	0.477233
144	D11	0.969707	0.3631	0.974886
145	D11	0.97227	0.62734	0.979926
146	D11	0.527481	0.506077	0.395659
147	D11	0.528325	0.513591	0.660439

148	D11	0.530702	0.511526	0.898953
149	D11	0.472318	0.493664	0.604269
150	D11	0.471424	0.486113	0.339518
151	D11	0.470216	0.488838	0.101505
152	D11	0.400975	0.875955	0.891401
153	D11	0.025221	0.870842	0.018004
154	D11	0.163751	0.641627	0.653317
155	D11	0.027558	0.372307	0.020128
156	D11	0.033016	0.137481	0.025541
157	D11	0.163837	0.011906	0.523008
158	D11	0.398948	0.008084	0.522933
159	D11	0.664497	0.013817	0.523606
160	D11	0.900162	0.011631	0.52364

Table 11.
Reported H-$H^{\delta-}$ distances (Å) for fully loaded D11 site system on γ-$Mg(BH_4)_2$. Distances larger than 3.00 Å have been omitted.

Site D11	Site $H^{\delta-}$	Distance (Å)
132	108	2.96
132	117	2.61
132	118	2.99
133	109	2.96
133	119	2.99
134	110	2.91
135	111	2.99
135	121	2.61
135	127	2.97
136	123	2.79
137	122	2.80
138	131	2.78
139	83	3.00
139	113	2.90
139	125	2.58
140	92	2.81
140	95	2.66
141	84	2.90
142	97	2.79
142	102	2.70
143	87	2.85
144	99	2.55
145	91	2.87
145	104	2.90
146	100	3.00
146	107	2.57
147	88	2.95
147	101	2.86
148	75	2.99
148	114	2.83
148	129	2.63
149	106	2.56
150	89	2.95
150	100	2.86
151	74	3.00
151	115	2.83
151	128	2.64
152	93	2.81
152	94	2.65
153	130	2.78

154	108	2.94
154	118	3.00
155	90	2.87
155	105	2.90
156	112	2.90
156	124	2.58
157	86	2.85
158	110	2.99
158	120	2.61
158	126	2.98
159	111	2.90
160	96	2.79
160	103	2.69
161	85	2.89
162	109	2.97
162	116	2.62
162	119	2.98
163	98	2.55

Table 12.
Reported H-H distances (Å) between neighbor H_2 molecules for fully loaded D11 site system on γ-$Mg(BH_4)_2$. The H_2 bond length (0.75 Å) has been omitted, as well as distances larger than 3.00 Å have been omitted.

**D11**	**D11**	**Distance (Å)**
133	161	2.66
134	157	2.69
136	150	2.86
137	147	2.86
138	155	2.94
141	154	2.66
143	159	2.69
145	153	2.94

Structure of γ-Mg$(BH_4)_2$ with **fully loaded D22 site:**

Full Formula (Mg12 B24 H96 H24)
Reduced Formula: MgB2H8Ne2
abc : 13.536869 13.544572 13.384006
angles: 109.027246 109.264171 109.429859
Sites (156)

#	SP	a	b	c
0	Mg	0.249568	0.620571	0.374161
1	Mg	0.750351	0.378582	0.621847
2	Mg	0.247862	0.124172	0.872747
3	Mg	0.752275	0.875334	0.124321
4	Mg	0.124195	0.377814	0.75129
5	Mg	0.875902	0.621708	0.245395
6	Mg	0.123813	0.245567	0.373382
7	Mg	0.875525	0.753807	0.623236
8	Mg	0.37555	0.121853	0.250195
9	Mg	0.624292	0.87777	0.746967
10	Mg	0.377762	0.74905	0.124505
11	Mg	0.621766	0.250279	0.87154
12	B	0.059108	0.613816	0.30779
13	B	0.94106	0.385687	0.687222
14	B	0.561375	0.250827	0.4377
15	B	0.438156	0.747453	0.559029
16	B	0.182318	0.249336	0.812481
17	B	0.817703	0.750045	0.184267
18	B	0.311852	0.119367	0.059477
19	B	0.688814	0.880951	0.937831
20	B	0.121665	0.31024	0.561059
21	B	0.879626	0.690191	0.436238
22	B	0.191813	0.43353	0.3744
23	B	0.805831	0.565081	0.620367
24	B	0.382649	0.938616	0.18975
25	B	0.616086	0.060413	0.804864
26	B	0.936982	0.06179	0.249849
27	B	0.062856	0.937712	0.748337
28	B	0.74762	0.436595	0.057952
29	B	0.251513	0.562667	0.938426
30	B	0.309806	0.687784	0.248851
31	B	0.691555	0.312164	0.74828
32	B	0.246638	0.179714	0.309626
33	B	0.753251	0.820583	0.686939
34	B	0.434637	0.191219	0.871728
35	B	0.564558	0.807513	0.123716
36	H	0.415235	0.187655	0.144104
37	H	0.585372	0.81233	0.853377
38	H	0.079182	0.232527	0.77593
39	H	0.920733	0.766563	0.219611
40	H	0.538984	0.27084	0.352177
41	H	0.459528	0.729115	0.645663
42	H	0.042934	0.679402	0.266738
43	H	0.955091	0.317286	0.725021
44	H	0.317957	0.856854	0.084223
45	H	0.683878	0.143036	0.909828
46	H	0.351252	0.087671	0.803198
47	H	0.647491	0.911288	0.191635
48	H	0.184546	0.266997	0.542554
49	H	0.816986	0.733867	0.454597
50	H	0.272272	0.583197	0.227253
51	H	0.729198	0.416766	0.769854
52	H	0.264391	0.2145	0.414052
53	H	0.735171	0.785038	0.582364
54	H	0.851178	0.043894	0.268158
55	H	0.147573	0.957279	0.728246
56	H	0.764479	0.35037	0.041129
57	H	0.235368	0.649273	0.954174
58	H	0.231215	0.453942	0.308654
59	H	0.766997	0.544778	0.686548

60	H	0.087683	0.350957	0.305952
61	H	0.910227	0.647078	0.688325
62	H	0.414908	0.726256	0.271559
63	H	0.586283	0.273685	0.725084
64	H	0.955884	0.146921	0.230476
65	H	0.045332	0.851406	0.765792
66	H	0.454482	0.231022	0.805973
67	H	0.545102	0.76848	0.190113
68	H	0.19154	0.413419	0.645087
69	H	0.809458	0.586953	0.352345
70	H	0.14176	0.680916	0.412933
71	H	0.85937	0.321433	0.58147
72	H	0.314459	0.953792	0.22712
73	H	0.681241	0.043164	0.763845
74	H	0.218303	0.265888	0.916637
75	H	0.780496	0.732933	0.079889
76	H	0.228319	0.076234	0.271481
77	H	0.771093	0.923903	0.724262
78	H	0.648133	0.234837	0.452986
79	H	0.352025	0.76472	0.542257
80	H	0.728214	0.456797	0.143775
81	H	0.272171	0.541739	0.85347
82	H	0.270473	0.183848	0.041894
83	H	0.730667	0.816804	0.955501
84	H	0.318857	0.056445	0.975441
85	H	0.681806	0.943756	0.021913
86	H	0.176161	0.161516	0.739387
87	H	0.824515	0.838205	0.257653
88	H	0.57683	0.336842	0.522076
89	H	0.422073	0.660059	0.475657
90	H	0.077571	0.546374	0.239071
91	H	0.92226	0.451812	0.756849
92	H	0.449221	0.023743	0.187083
93	H	0.55114	0.976466	0.81024
94	H	0.481825	0.812287	0.055699
95	H	0.517041	0.185555	0.93912
96	H	0.052939	0.242958	0.579841
97	H	0.948667	0.756997	0.417351
98	H	0.241357	0.685408	0.160142
99	H	0.75958	0.314652	0.837294
100	H	0.33465	0.251958	0.315258
101	H	0.665561	0.748368	0.681815
102	H	0.018499	0.077156	0.338934
103	H	0.980401	0.923169	0.660724
104	H	0.836773	0.516978	0.073259
105	H	0.161544	0.482806	0.922558
106	H	0.260785	0.412867	0.439189
107	H	0.73701	0.58634	0.556269
108	H	0.187406	0.515896	0.442889
109	H	0.809378	0.482455	0.551215
110	H	0.311088	0.755966	0.336498
111	H	0.690747	0.244039	0.660969
112	H	0.079093	0.018514	0.838162
113	H	0.921688	0.979985	0.161546
114	H	0.584385	0.738456	0.058406
115	H	0.415354	0.260366	0.937724
116	H	0.058465	0.317544	0.477084
117	H	0.942392	0.682751	0.520361
118	H	0.02698	0.45137	0.684859
119	H	0.974451	0.549462	0.312735
120	H	0.548975	0.079216	0.735811
121	H	0.448131	0.919448	0.259986
122	H	0.254852	0.33733	0.818343
123	H	0.745552	0.66241	0.179258
124	H	0.158854	0.175313	0.237923
125	H	0.841368	0.82556	0.758876
126	H	0.481851	0.161312	0.422838
127	H	0.518421	0.835672	0.572989
128	H	0.337296	0.57737	0.022961
129	H	0.660964	0.421655	0.974177

130	H	0.243217	0.050604	0.07717
131	H	0.757087	0.949864	0.919942
132	D22	0.21454	0.818942	0.664913
133	D22	0.709332	0.053455	0.413086
134	D22	0.325216	0.404499	0.174941
135	D22	0.610609	0.571028	0.809153
136	D22	0.650724	0.33196	0.260817
137	D22	0.306499	0.680091	0.759379
138	D22	0.789271	0.222647	0.170407
139	D22	0.185607	0.94866	0.377073
140	D22	0.044291	0.718399	0.91062
141	D22	0.901129	0.202899	0.867559
142	D22	0.395069	0.346612	0.676054
143	D22	0.556422	0.64736	0.337697
144	D22	0.276641	0.827834	0.680845
145	D22	0.100431	0.778727	0.949575
146	D22	0.205627	0.90247	0.369421
147	D22	0.337911	0.654638	0.73093
148	D22	0.604475	0.651359	0.320336
149	D22	0.674777	0.588793	0.825058
150	D22	0.389202	0.434288	0.188275
151	D22	0.44457	0.353168	0.660523
152	D22	0.71234	0.345027	0.269815
153	D22	0.953009	0.191393	0.874762
154	D22	0.850278	0.270349	0.189349
155	D22	0.77601	0.092677	0.455188

Table 13.
Reported H-$H^{\delta-}$ distances (Å) for 50% loaded D22 site system on γ-$Mg(BH_4)_2$. Distances larger than 3.00 Å have been omitted.

Site D22	Site $H^{\delta-}$	Distance (Å)
132	46	2.99
132	55	2.38
132	70	2.88
132	79	2.97
133	47	2.60
133	78	2.79
133	121	2.92
134	50	2.69
134	58	2.56
134	74	2.87
134	82	2.61
135	83	2.76
136	36	2.64
136	40	2.35
136	69	2.93
136	80	2.84
137	41	2.98
137	68	2.98
137	81	2.58
138	45	2.93
138	56	2.84
138	64	2.77
139	76	2.60
140	57	2.98
140	61	2.54
140	91	2.99
141	43	2.94
141	73	2.55
141	99	2.82
142	48	2.41
142	52	2.88
142	66	2.79
143	49	2.94
143	53	2.80
143	62	2.54
143	67	2.95
143	89	2.99
144	46	2.98
144	55	2.93
144	70	2.96

144	79	2.45
145	44	2.49
145	57	2.92
145	61	2.99
145	65	2.90
146	42	2.59
146	72	2.88
146	110	2.81
147	37	2.79
147	41	2.43
147	68	2.77
147	81	2.74
148	49	2.42
148	53	2.89
148	67	2.82
149	51	2.63
149	59	2.58
149	75	2.84
149	83	2.69
150	50	2.97
150	82	2.81
151	48	2.94
151	52	2.82
151	63	2.54
151	66	2.94
151	88	2.98
152	40	2.93
152	69	2.74
152	80	2.62
153	38	2.51
154	56	2.68
154	60	2.68
154	64	2.61
155	47	2.99
155	54	2.96
155	71	2.59
155	78	2.99

Table 14.
Reported H-H distances (Å) between neighbor H_2 molecules for 50% loaded D22 site system on γ-$Mg(BH_4)_2$. The H_2 bond length (0.75 Å) has been omitted, as well as distances larger than 3.00 Å have been omitted.

Site D22	Site D22	Distance (Å)
132	137	2.96
135	142	2.84
135	151	2.55
137	144	2.63
138	152	2.50
143	150	2.52
144	147	2.93
148	150	2.79
152	154	2.75

a)

b)

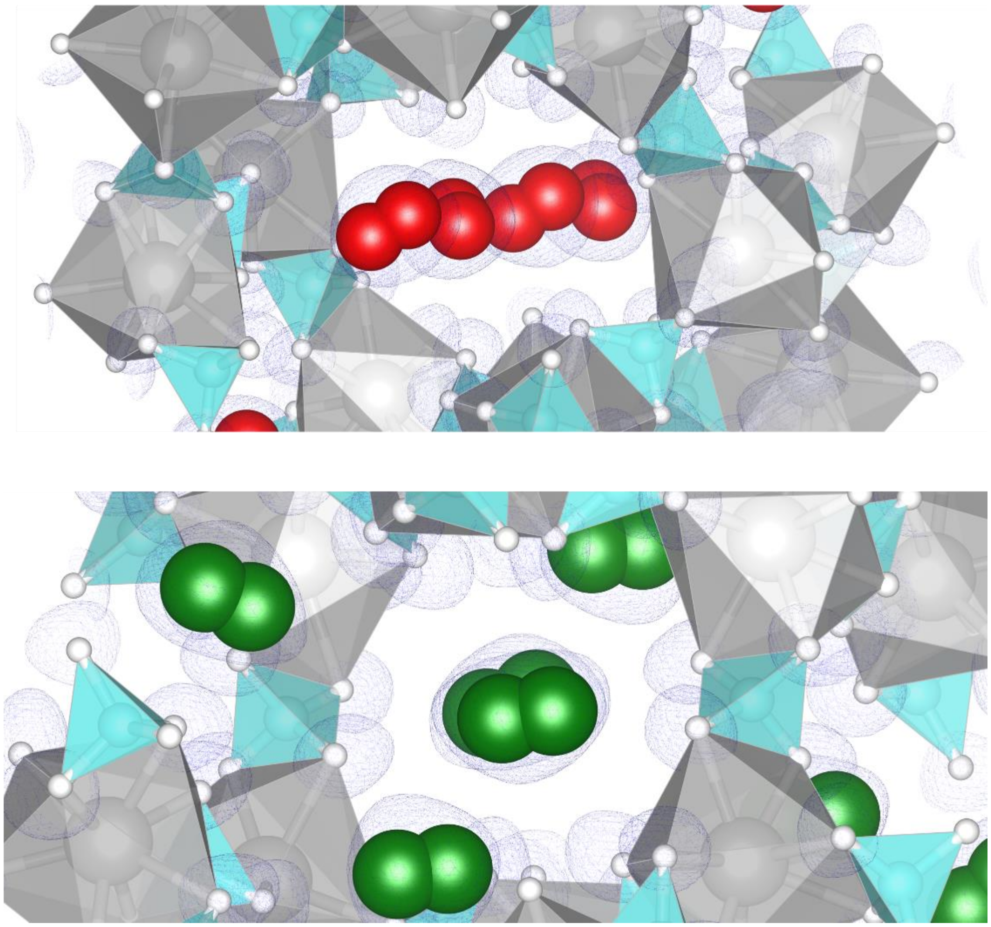

Fig. 26. Electron localization function plots of H_2 molecule adsorbed on D11 sites (a) and D22 sites (b) of γ-$Mg(BH_4)_2$.

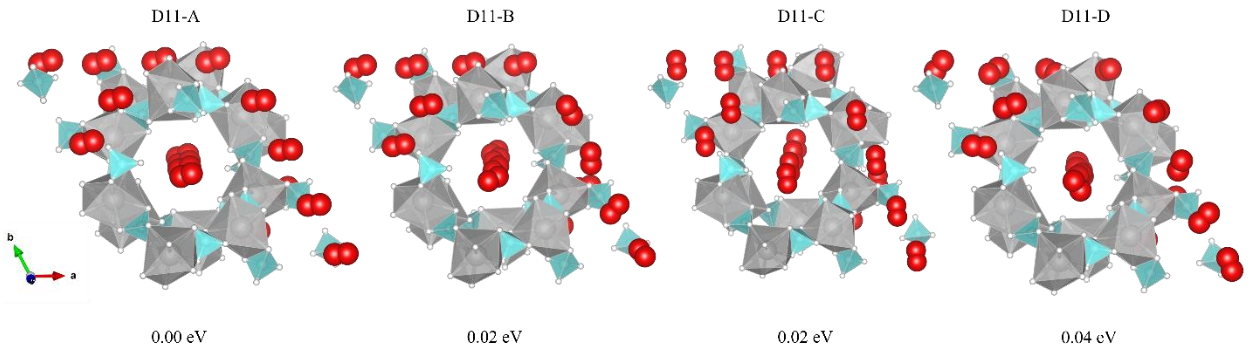

Fig. 27. Different H_2 orientations of fully loaded D11 site and their corresponding relative energies with respect to the lowest energy system.

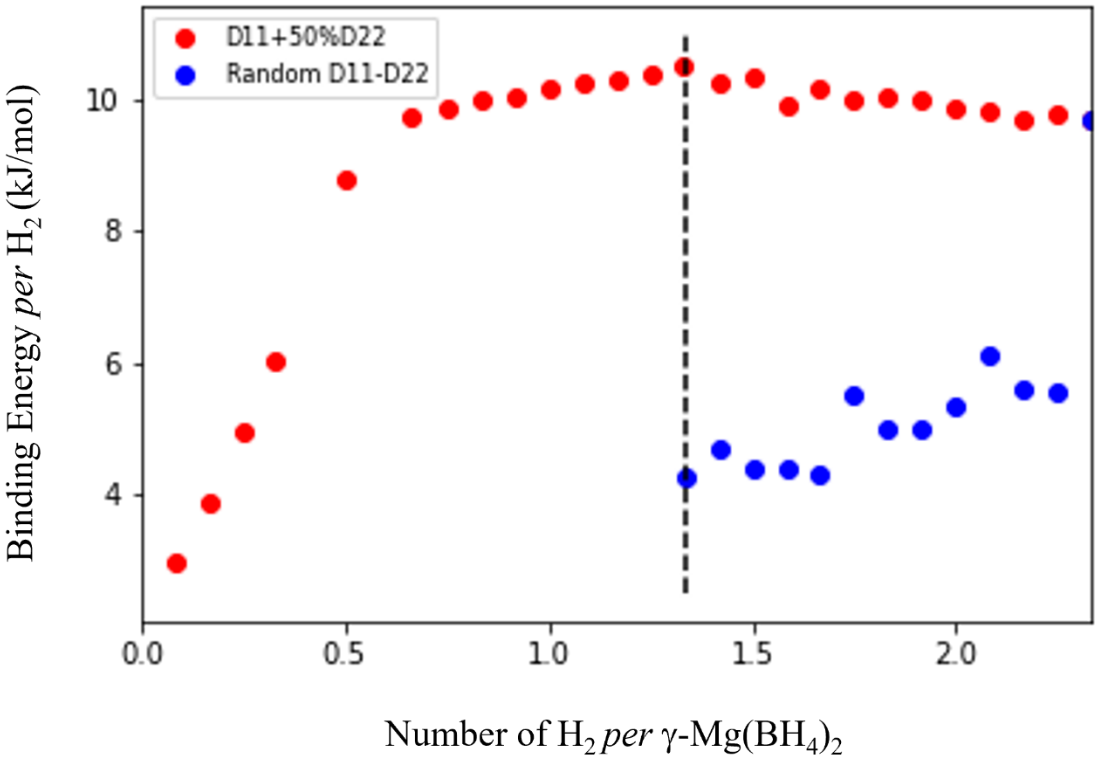

Fig. 28. Binding energy *per* H_2 molecule adsorbed on γ-$Mg(BH_4)_2$ defined as in Figure 25. Black dashed line indicates the ratio (1.33 H_2/Mg) where D11 site is fully loaded. Red dots show the sequential loading where the uptake of D22 sites occurs after the full loading of D11 (from 1.33 H_2/Mg), whereas blue dots show the energies for configuration with H_2 adsorption mixed on both sites, i.e., D11 is not fully loaded before to start D22 adsorption.

a) b)

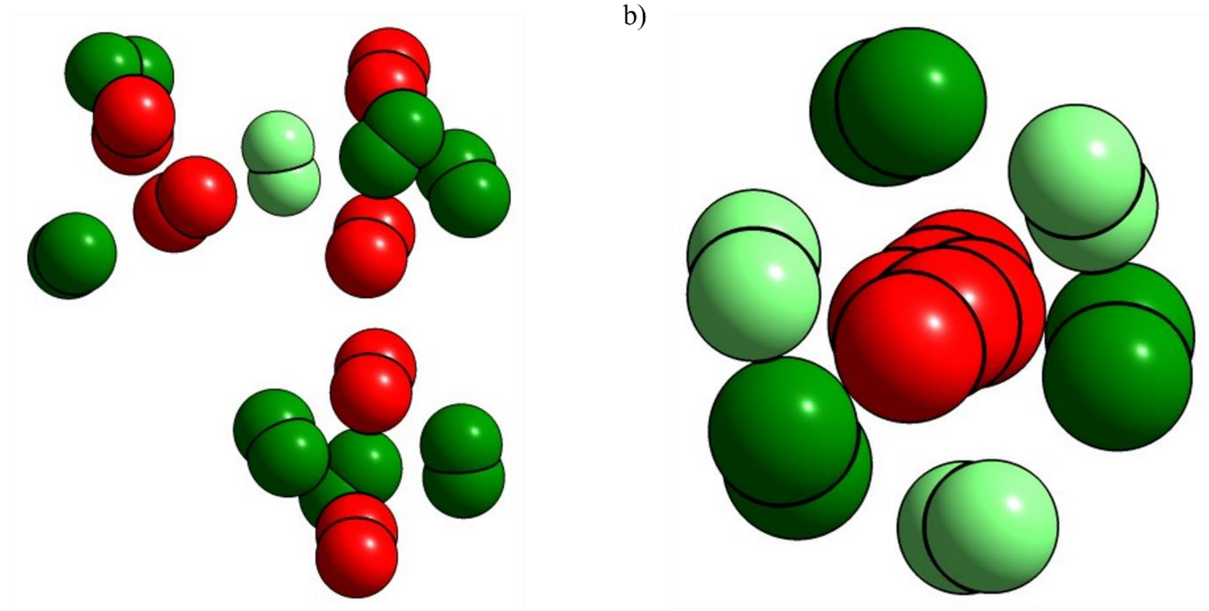

Fig. 29. (A) Sketches of H_2 distribution on fully loaded system, forming bipyramidal clusters also found experimentally. These clusters are linked by sharing the H_2 (D22) vertex (light green). Two units of 5 H_2 clusters are connected by means of two H_2 molecules on D11 without sharing vertex **(B)** Top view of two bipyramid cluster. Dark and light green correspond to the H_2 on D22 sites of the different trigonal bipyramid cluster.

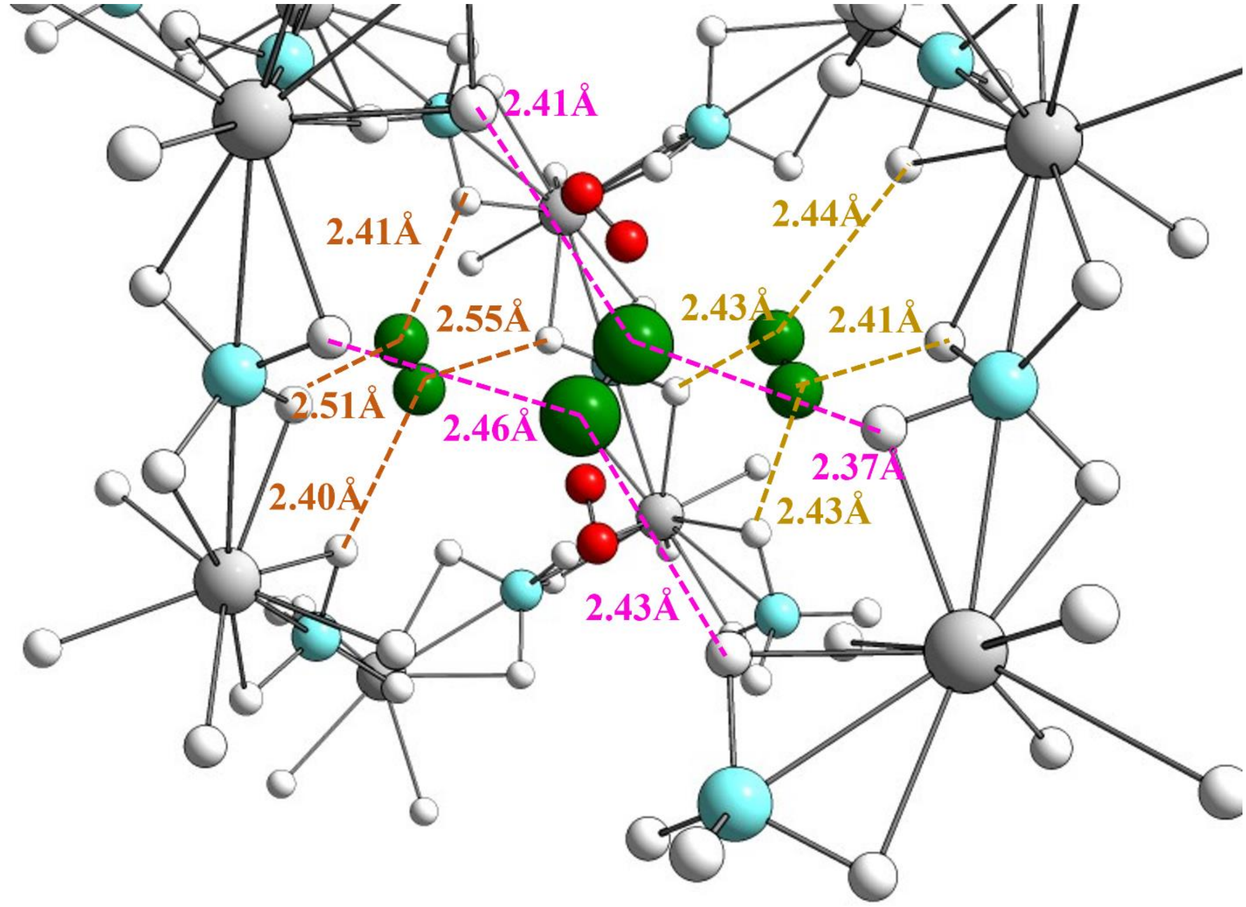

Fig. 30. Schematic representation of the connectivity between BH_4 tetrahedra and the H_2 adsorbed on D22 sites. The three H_2 (D22, green) molecules form the base of the bipyramid cluster completed by two H_2 molecules adsorbed on D11 (red), that are placed on the top and on the bottom of the bipyramid. This structure is the full loaded configuration (full D11 and 50% D22). Several H_2, BH_4, and MgH_8 polyhedral have been omitted for clarity.

During the optimization process, the H_2 molecules are re-oriented and reorganized to find the energy minimum. All the attempts to find meta-stable systems slightly higher in energy reach the minimum structure after the optimization. To further evaluate the effect of H_2 directionality, single-point calculations were performed. As illustrated in Figure S31, the directionality plays a key role. Note that structure b) is close in energy since the H_2 molecules adsorbed on D22 sites have similar orientation than the optimized structure. In contrast, structure c has different random directionality, which implies larger energies. Tables 15 and 16 collect the host-guest and guest-guest distances of the optimized structure (a) and single-point (c). It can be observed that shorter distances provide lower stability.

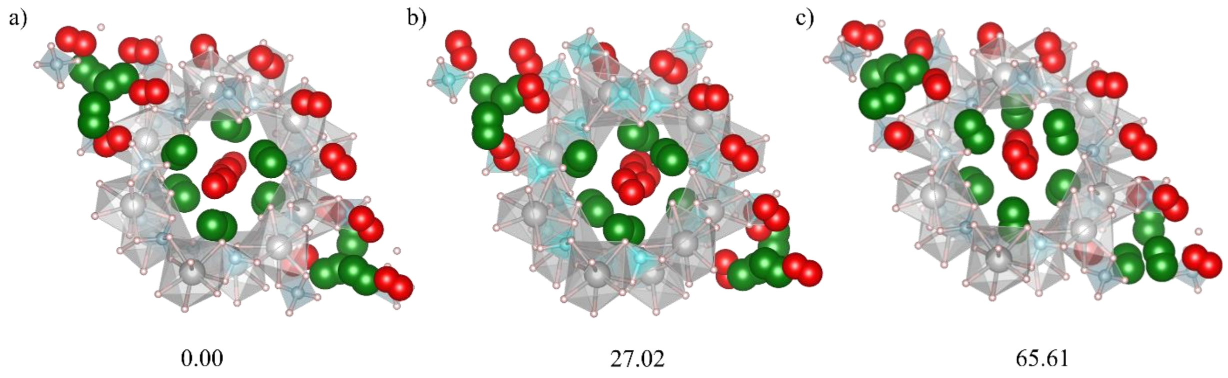

Fig. 31. (A) Sketch of optimized γ-$Mg(BH_4)_2{\cdot}2.33H_2$ structure. (B) and (C) Sketches of non-optimized γ-$Mg(BH_4)_2{\cdot}2.33H_2$ structures to investigate the influence of H_2 directionality on the energetics. The relative total energies are in kJ/mol.

Table 15.
Reported H-H distances (Å) between neighbor H_2 molecules for the optimized γ-$Mg(BH_4)_2 \cdot 2.33H_2$ structure (Figure S31a). The H_2 bond length (0.75 Å) has been omitted, as well as distances larger than 3.00 Å.

Site D22	Site $H^{\delta-}$	Distance **(Å)**	Site D11	Site $H^{\delta-}$	Distance **(Å)**	Site D11	Site D22	Distance **(Å)**
164	46	2.80	132	70	2.98	133	168	2.88
164	55	2.41	132	108	2.80	134	171	2.87
164	70	2.55	132	117	2.75	134	178	2.70
164	79	2.75	132	118	2.83	135	173	2.91
165	47	2.52	133	69	2.98	136	166	2.64
165	54	2.80	133	109	2.73	136	180	2.69
165	71	2.82	133	119	2.83	136	182	2.83
165	78	2.40	134	110	2.98	136	184	2.81
166	50	2.41	134	126	2.87	137	169	2.94
166	58	2.76	135	73	2.80	137	174	2.81
166	74	2.37	135	111	2.53	138	185	2.87
166	82	2.78	135	127	2.85	138	186	2.89
167	51	2.73	136	115	2.80	141	176	2.87
167	59	2.43	136	128	2.85	141	177	2.85
167	75	2.72	137	114	2.81	143	187	2.91
167	83	2.47	137	122	2.60	145	181	3.00
168	36	2.81	137	129	2.95	146	182	2.77
168	40	2.38	138	131	2.95	147	167	2.77
168	69	2.52	139	83	2.92	147	169	2.97
168	80	2.80	139	113	2.78	147	174	2.82
169	37	2.54	139	125	2.58	147	179	2.66
169	41	2.76	140	36	2.3	147	183	2.59
169	68	2.77	140	92	2.72	148	167	2.76
169	81	2.39	140	95	2.63	149	168	2.66
170	45	2.87	141	84	2.93	149	175	2.58
170	56	2.41	141	93	2.94	149	180	2.79
170	60	2.51	142	48	2.89	149	182	2.75
170	64	2.75	142	86	2.68	150	180	2.78
171	39	2.44	142	102	2.71	150	184	3.00
171	42	2.74	143	103	2.94	151	167	2.83
171	72	2.76	144	90	2.47	151	169	2.80
171	76	2.40	144	99	2.87	151	174	2.68

172	44	2.84	145	104	2.93	151	181	2.66
172	57	2.40	146	58	2.86	152	187	2.98
172	61	2.51	146	89	2.88	155	165	2.95
172	65	2.75	146	100	2.49	156	165	2.90
173	38	2.75	147	106	2.81	156	186	2.79
173	43	2.44	148	59	2.81	157	164	2.89
173	73	2.43	148	88	2.86	157	171	2.96
173	77	2.80	148	101	2.50	159	164	3.00
173	99	2.92	148	107	2.98	159	172	2.99
174	48	2.46	149	89	2.99	159	179	2.91
174	52	2.75	149	107	2.81	160	173	2.69
174	63	2.78	150	115	2.87	160	185	2.91
174	66	2.53	150	123	2.56	163	177	2.99
175	49	2.82	150	128	2.93			
175	53	2.40	151	114	2.82			
175	62	2.45	151	129	2.83			
175	67	2.84	152	71	2.90			
175	89	2.94	152	109	2.89			
176	46	2.50	152	116	2.63			
176	55	2.79	152	119	2.99			
176	70	2.86	153	49	2.85			
176	79	2.42	153	87	2.60			
177	44	2.53	153	103	2.78			
177	57	2.74	154	110	2.91			
177	61	2.81	154	120	2.51			
177	65	2.39	154	126	2.97			
178	39	2.77	155	121	2.72			
178	42	2.41	155	127	2.90			
178	72	2.44	156	92	2.98			
178	76	2.77	157	86	3.00			
178	110	2.95	158	37	2.87			
179	37	2.82	158	93	2.75			
179	41	2.38	158	94	2.52			
179	68	2.51	159	108	2.90			
179	81	2.78	159	118	2.91			
180	49	2.48	160	105	2.84			
180	53	2.75	161	82	2.96			
180	62	2.76	161	112	2.75			
180	67	2.52	161	124	2.62			
181	51	2.42	162	91	2.73			
181	59	2.75	162	98	2.73			
181	75	2.35	162	104	2.93			
181	83	2.78	163	113	3.00			
182	50	2.74	163	130	2.83			
182	58	2.46						

182	74	2.72						
182	82	2.43						
183	48	2.83						
183	52	2.42						
183	63	2.44						
183	66	2.83						
183	88	2.97						
184	36	2.55						
184	40	2.76						
184	69	2.76						
184	80	2.38						
185	38	2.41						
185	43	2.78						
185	73	2.73						
185	77	2.43						
186	45	2.55						
186	56	2.74						
186	60	2.83						
186	64	2.40						
187	47	2.77						
187	54	2.41						
187	71	2.54						
187	78	2.78						

Table 16.
Reported H-H distances (Å) between neighbor H_2 molecules for the non-optimized γ-$Mg(BH_4)_2{\cdot}2.33H_2$ structure (Figure 31c). The H_2 bond length (0.75 Å) has been omitted, as well as distances larger than 3.00 Å.

Site D22	Site $H^{\delta-}$	Distance **(Å)**	Site D11	Site $H^{\delta-}$	Distance **(Å)**	Site D11	Site D22	Distance **(Å)**
164	46	2.80	132	70	2.98	133	168	2.88
164	55	2.42	132	108	2.80	133	184	2.24
164	70	2.55	132	117	2.74	134	171	2.97
164	79	2.75	132	118	2.84	134	178	2.70
165	47	2.52	133	69	2.98	136	166	2.64
165	54	2.80	133	109	2.73	136	175	2.76
165	71	2.82	133	119	2.83	136	180	2.69
165	78	2.40	134	110	2.98	136	182	2.42
166	50	2.41	134	126	2.87	137	174	2.81
166	58	2.76	135	73	2.80	137	183	2.77
166	74	2.37	135	111	2.53	138	173	2.53
166	82	2.78	135	127	2.85	138	185	2.87
167	51	2.68	136	115	2.80	138	186	2.89
167	75	1.86	136	128	2.85	141	172	2.56
167	83	2.94	137	114	2.81	141	176	2.56
167	104	2.63	137	122	2.60	141	177	2.82
167	123	3.00	137	129	2.95	141	182	2.77
167	129	2.54	138	131	2.95	143	187	2.91
168	36	2.81	139	83	2.92	145	167	2.95
168	40	2.38	139	113	2.78	147	169	1.98
168	69	2.52	139	125	2.58	147	174	2.82
168	80	2.80	140	36	2.93	147	179	2.66
169	37	2.64	140	92	2.72	147	183	2.99
169	41	2.33	140	95	2.63	148	169	2.47
169	68	2.93	141	84	2.93	149	168	2.66
169	106	3.00	141	93	2.94	149	175	2.98
170	54	2.91	142	48	2.89	149	180	2.79
170	60	2.54	142	86	2.68	150	175	2.71
170	64	1.82	142	102	2.71	150	180	2.78
170	85	3.00	143	103	2.94	151	167	2.51
171	39	2.12	144	90	2.47	151	169	2.83
171	42	1.88	144	99	2.87	151	174	2.68

172	55	2.92	145	104	2.93	151	181	2.66
172	61	2.67	146	58	2.86	151	187	2.78
172	65	1.88	146	89	2.88	152	170	2.96
172	84	2.89	146	100	2.49	152	184	2.96
173	73	2.11	147	106	2.81	152	187	2.78
173	77	2.21	148	59	2.81	155	165	2.96
174	48	2.48	148	88	2.86	156	165	2.90
174	52	2.75	148	101	2.50	156	170	2.67
174	63	2.78	148	107	2.98	156	186	2.80
174	66	2.53	149	89	2.99	157	164	2.89
175	49	1.86	149	107	2.81	159	164	3.00
175	53	2.77	150	115	2.87	159	172	2.90
175	67	2.88	150	123	2.56	159	176	2.83
175	69	2.73	150	128	2.93	159	179	2.91
175	123	2.64	151	114	2.82	160	173	2.77
176	46	2.77	151	129	2.83	160	185	2.92
176	55	2.86	152	71	2.90	161	173	2.98
176	70	2.73	152	109	2.89	163	177	2.99
176	79	2.29	152	116	2.63			
177	44	2.53	152	119	2.99			
177	57	2.74	153	49	2.85			
177	61	2.81	153	87	2.60			
177	65	2.39	153	103	2.78			
178	39	2.77	154	110	2.91			
178	42	2.41	154	120	2.51			
178	72	2.44	154	126	2.97			
178	76	2.77	155	121	2.72			
178	110	2.95	155	127	2.90			
179	37	2.82	156	92	2.98			
179	41	2.38	157	86	3.00			
179	68	2.51	158	37	2.87			
179	81	2.78	158	93	2.75			
180	49	2.49	158	94	2.52			
180	53	2.75	159	108	2.90			
180	62	2.76	159	118	2.91			
180	67	2.52	160	105	2.84			
181	51	2.42	161	82	2.96			
181	59	2.75	161	112	2.75			
181	75	2.35	161	124	2.62			
181	83	2.78	162	91	2.74			
182	50	2.54	162	98	2.73			
182	74	2.00	162	104	2.93			
182	105	2.65	163	113	3.00			
182	128	2.44	163	130	2.84			
183	48	1.84						

183	52	2.75						
183	66	2.92						
183	68	2.71						
183	122	2.65						
184	40	2.79						
184	69	2.08						
184	80	2.36						
184	109	2.99						
185	38	2.41						
185	43	2.78						
185	73	2.73						
185	77	2.43						
186	45	2.55						
186	56	2.74						
186	60	2.83						
186	64	2.40						
187	47	2.77						
187	54	2.41						
187	71	2.54						
187	78	2.78						

References

38. V. Ban, A. V. Soloninin, A.V. Skripov, J. Hadermann, A. Abakumov, Y. Filinchuk. Pressure-collapsed amorphous $Mg(BH_4)_2$: an ultra-dense complex hydride showing a reversible transition to the porous framework *J. Phys. Chem. C*, **118**, 23402-23408 (2014).
39. Y. Filinchuk, R. Černý, H. Hagemann, Insight into $Mg(BH_4)_2$ with synchrotron X-ray diffraction: Structure revision, crystal chemistry, and anomalous thermal expansion. *Chem. Mater.* **21**, 925–933 (2009).
40. H. Oh, I. Savchenko, A. Mavrandonakis, T. Heine, M. Hirscher, Highly effective hydrogen isotope separation in nanoporous metal-organic frameworks with open metal sites: direct measurement and theoretical analysis. *ACS Nano* **8**, 761-770 (2014).
41. W. L. Queen, E. D. Bloch, C. M. Brown, M. R. Hudson, J. A. Mason, L. J. Murray, A. J. Ramirez-Cuesta, V. K. Peterson, J. R. Long, Hydrogen adsorption in the metal–organic frameworks Fe_2(dobdc) and $Fe_2(O_2)$(dobdc). *Dalton Trans.*, **41**, 4180-4187 (2012).
42. V. K. Peterson, Y. Liu, C. M. Brown, C. J. Kepert, V. K. Peterson, Y. Liu, C. M. Brown, C. J. Kepert, Neutron powder diffraction study of D_2 sorption in Cu_3(1,3,5-benzenetricarboxylate)$_2$. *J. Am. Chem. Soc.*, **128**, 15578-15579 (2006).
43. P. Krawiec, M. Kramer, M. Sabo, R. Kunschke, H. Fröde, S. Kaskel, Improved hydrogen storage in the metal-organic framework $Cu_3(BTC)_2$. *Adv. Eng. Mater.*, **8**, 293-296 (2006).
44. Y. Liu, H. Kabbour, C. M. Brown, D. A. Neumann, C. C. Ahn, Increasing the density of adsorbed hydrogen with coordinatively unsaturated metal centers in metal-organic frameworks. *Langmuir*, **24**, 4772-4777 (2008).
45. I. F. Silvera, The solid molecular hydrogens in the condensed phase: Fundamentals and static properties. *Rev. Mod. Phys.* **52**, 393-452 (1980).
46. T. A. Strobel, A. J. Ramirez-Cuesta, L. L. Daemen, V. S. Bhadram, T. A. Jenkins, C. M. Brown, Y. Q. Cheng, Quantum dynamics of H_2 trapped within organic clathrate cages. *Phys. Rev. Lett.* **120**, 120402 (2018).
47. L. Ulivi, M. Celli, A. Giannasi, A. J. Ramirez-Cuesta, D. J. Bull, M. Zoppi, Quantum rattling of molecular hydrogen in clathrate hydrate nanocavities. *Phys. Rev. B* **76**, 161401(R) (2007).
48. I. Weinrauch, I. Savchenko, D. Denysenko, S. M. Souliou, H. H. Kim, M. Le Tacon, L. L. Daemen, Y. Cheng, A. Mavrandonakis, A. J. Ramirez-Cuesta, D. Volkmer, G. Schutz, M. Hirscher, T. Heine, Capture of heavy hydrogen isotopes in a metal-organic framework with active Cu(I) sites. *Nat. Commun.* **8**, 14496 (2017).
49. J. A. Young, J. U. Koppel, Slow neutron scattering by molecular hydrogen and deuterium. *Phys. Rev.* **135**, A603-A611 (1964).
50. I. F. Silvera, V. V. Goldman, The isotropic intermolecular potential for H_2 and O_2 in the solid and gas phases. *J. Chem. Phys.* **69**, 4209-4213 (1978).
51. R. Balderas-Xicohténcatl, "High-density hydrogen monolayer formation and isotope diffusion in porous media", thesis, University of Stuttgart, Stuttgart, Germany (2019).
