# Supplementary figures and images for: Small-pore hydridic frameworks store densely packed hydrogen
Source: Nat Chem. 2024 Feb 6;16(5):809–16. doi: 10.1038/s41557-024-01443-x (PMC11087247; doi:10.1038/s41557-024-01443-x)

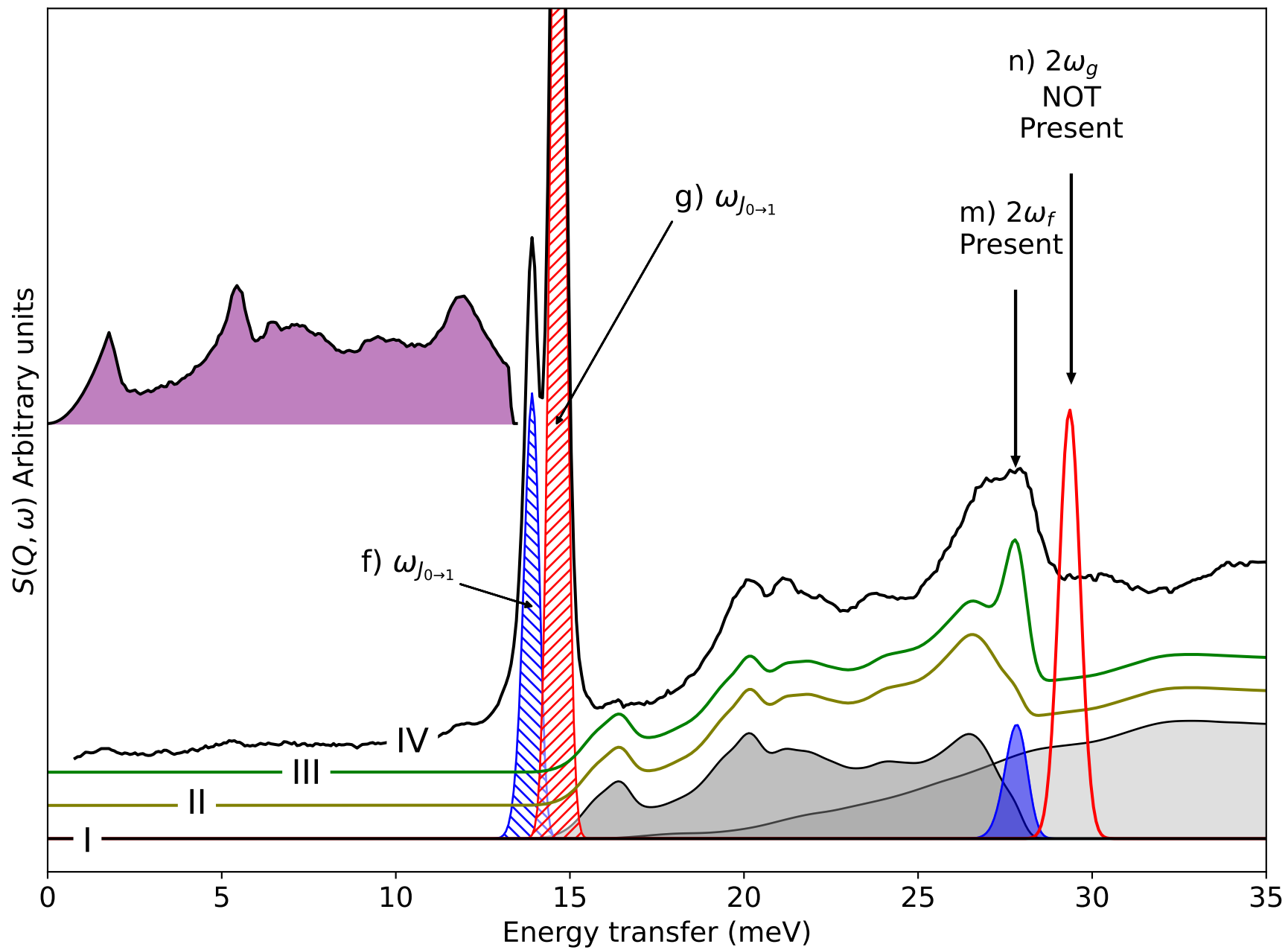

Supplement: Supplementary file 11 — Pdf in high res [file 41557_2024_1443_MOESM11_ESM.zip › Fig3a_high_resolution.pdf]

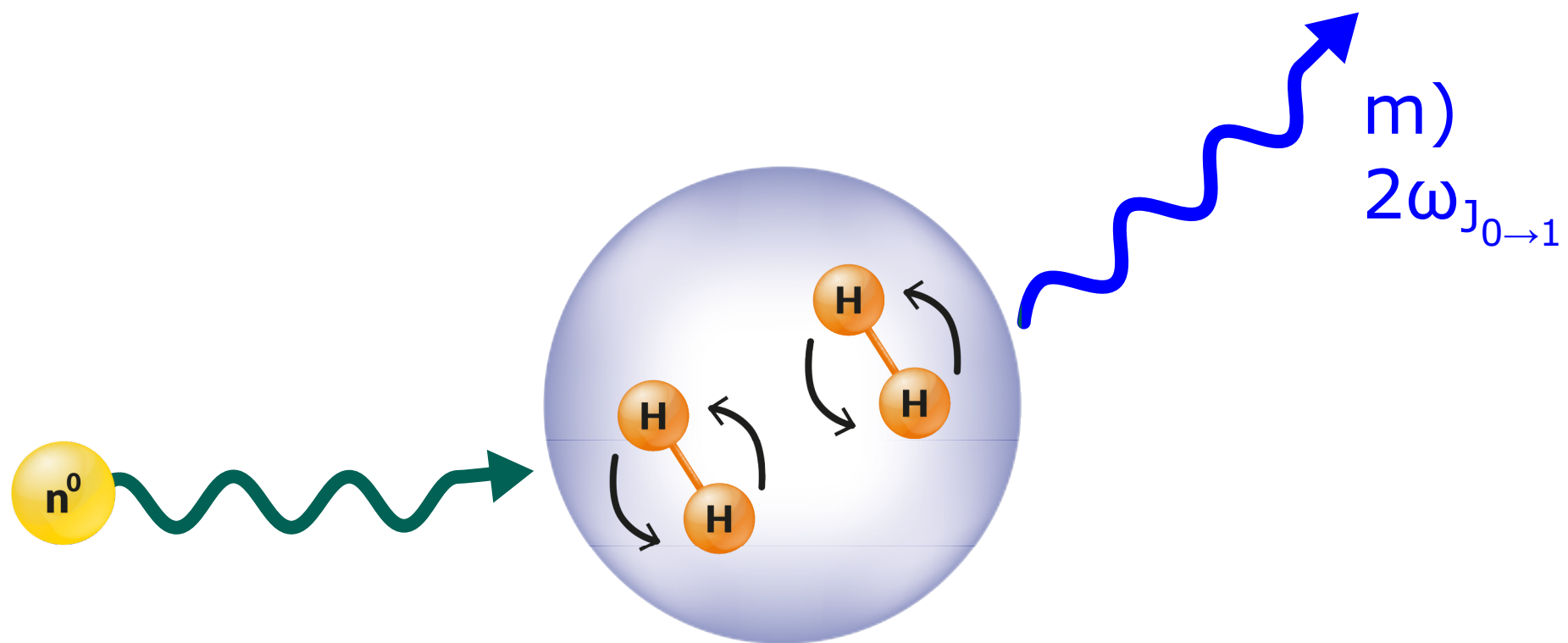

Supplement: Supplementary file 11 — Pdf in high res [file 41557_2024_1443_MOESM11_ESM.zip › Fig3b_high_resolution.pdf]
