# Supplementary material for: Small-pore hydridic frameworks store densely packed hydrogen
Source: Nat Chem. 2024 Feb 6;16(5):809–16. doi: 10.1038/s41557-024-01443-x (PMC11087247; doi:10.1038/s41557-024-01443-x)
Supplement: Supplementary file 12 — Pdf and Excell files, Eps and text files [file 41557_2024_1443_MOESM12_ESM.zip › Fig4-5.pptx]

## Slide 1
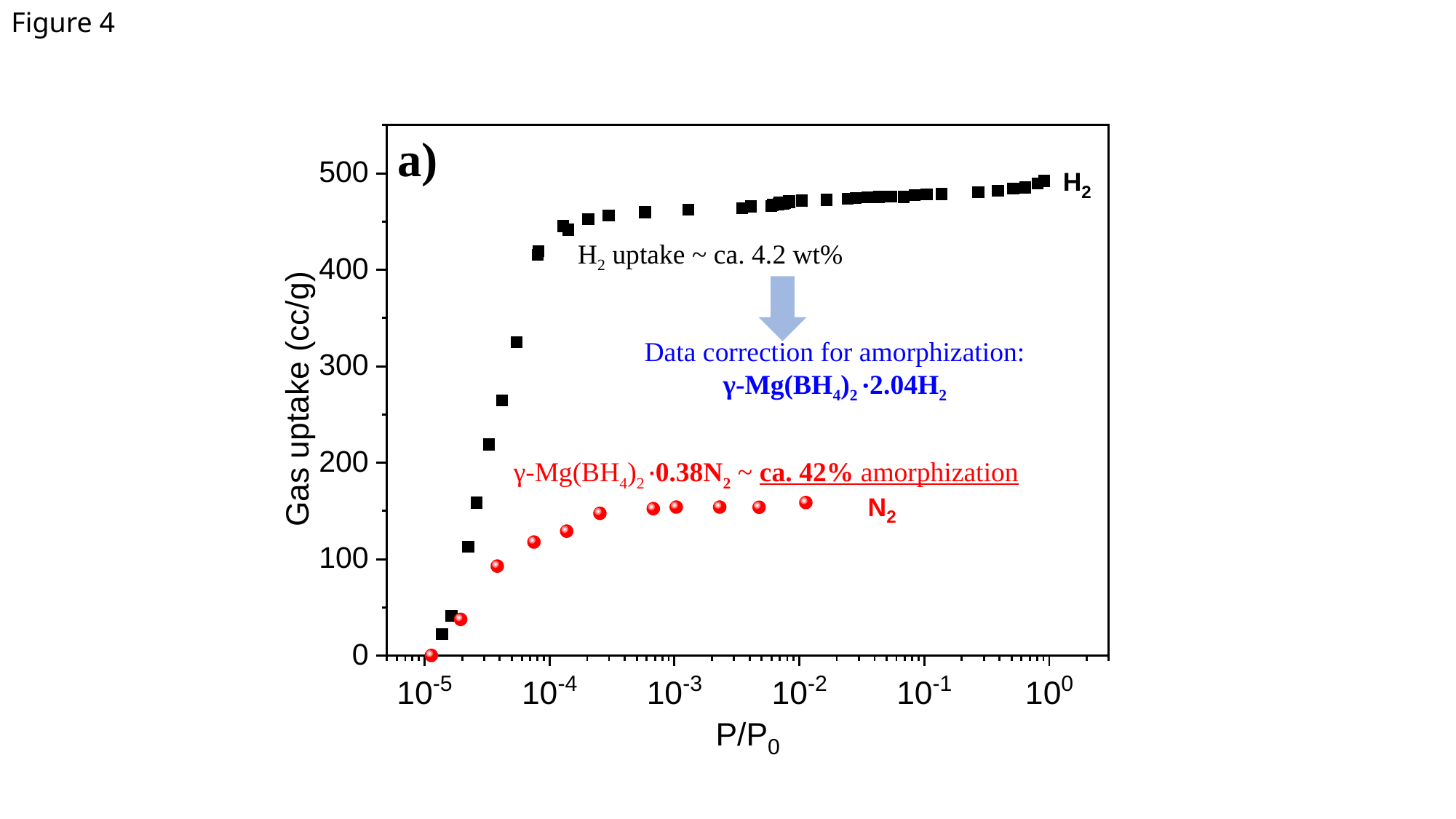

Figure 4
a)
H2 uptake ~ ca. 4.2 wt%
Data correction for amorphization:
 γ-Mg(BH4)2 ‧2.04H2
γ-Mg(BH4)2 ‧0.38N2 ~ ca. 42% amorphization

## Slide 2
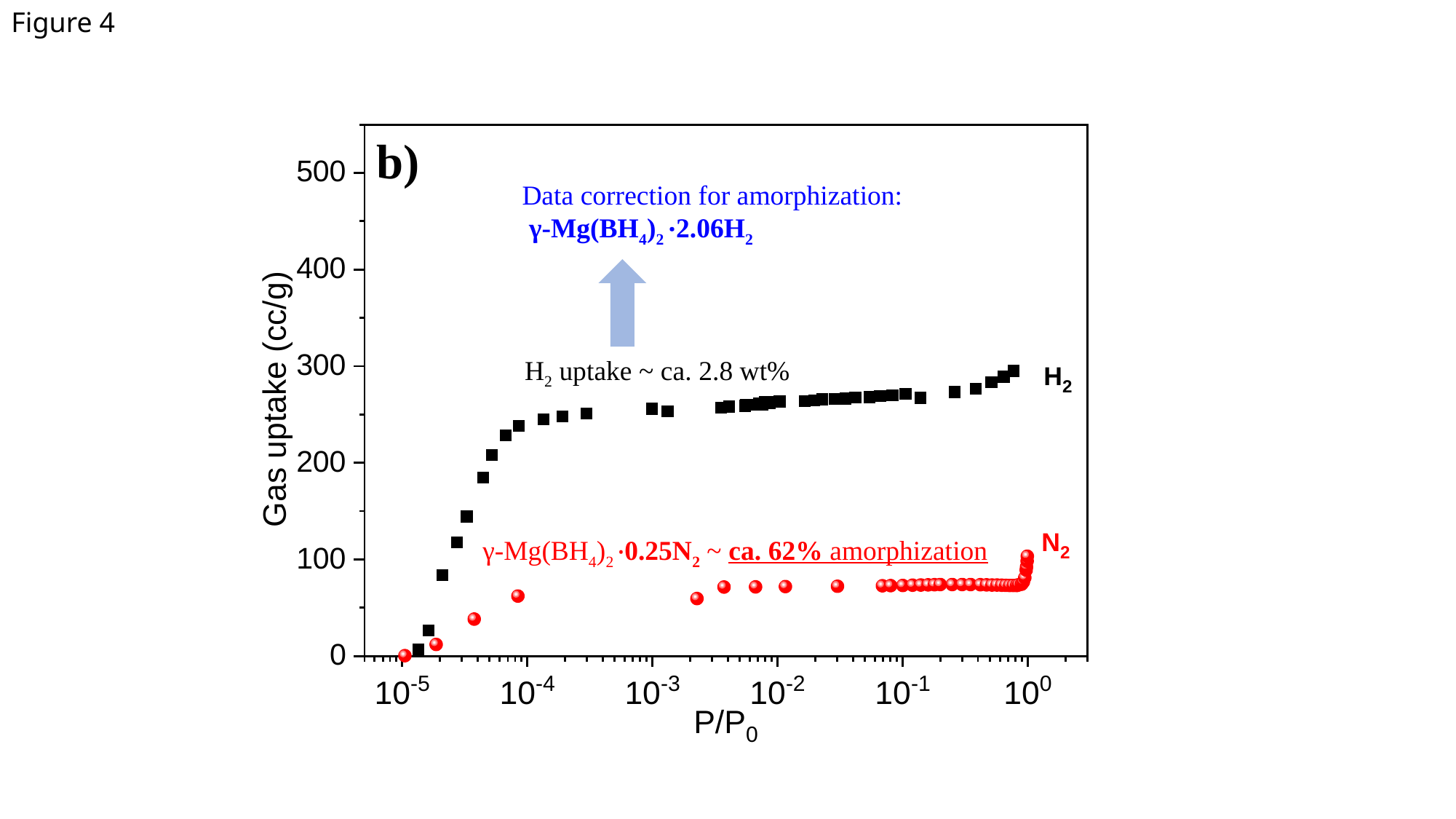

Figure 4
b)
Data correction for amorphization:
 γ-Mg(BH4)2 ‧2.06H2
H2 uptake ~ ca. 2.8 wt%
γ-Mg(BH4)2 ‧0.25N2 ~ ca. 62% amorphization

## Slide 3
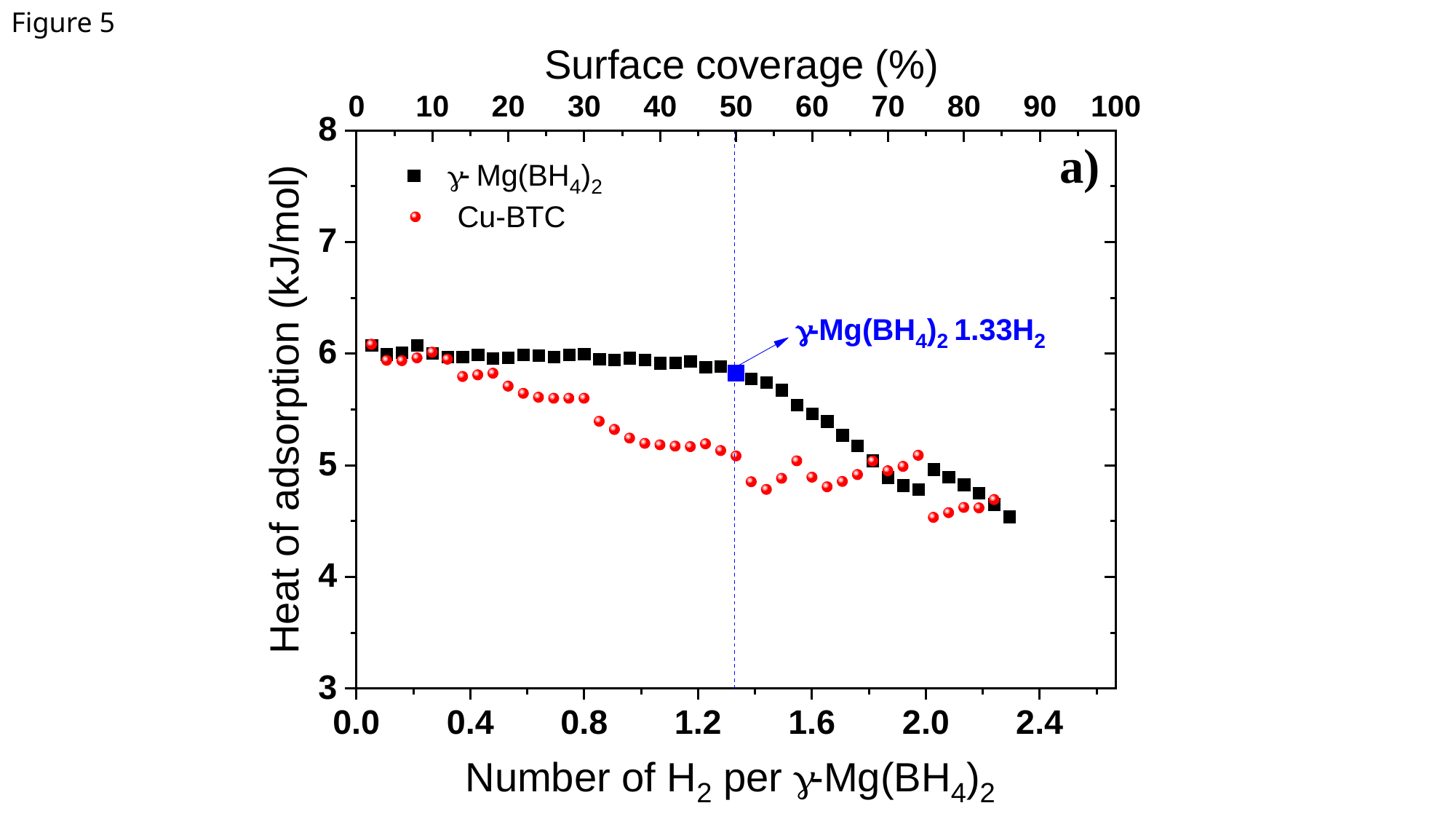

Figure 5
a)

## Slide 4
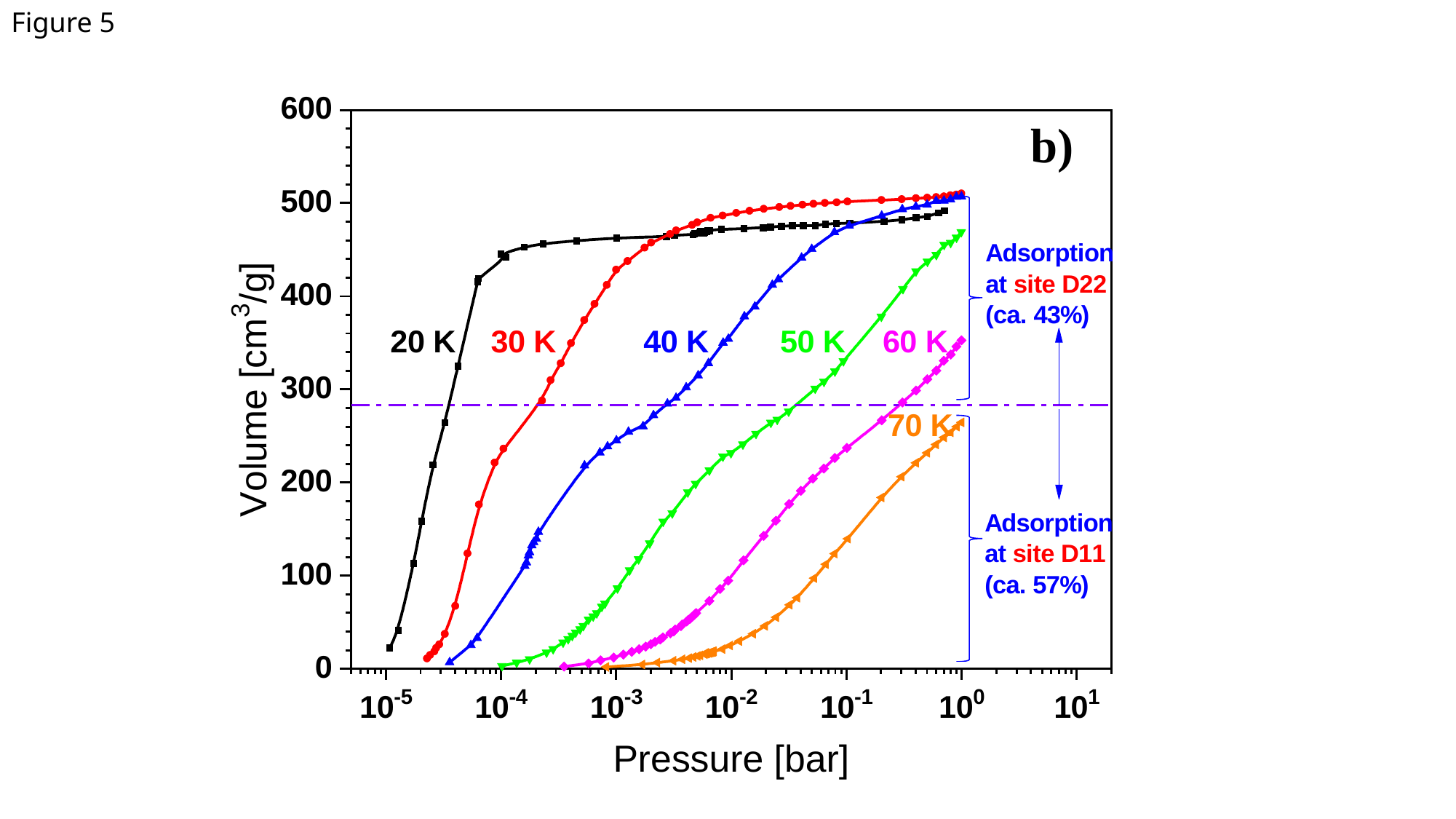

Figure 5
b)
