# Supplementary material for: Small-pore hydridic frameworks store densely packed hydrogen
Source: Nat Chem. 2024 Feb 6;16(5):809–16. doi: 10.1038/s41557-024-01443-x (PMC11087247; doi:10.1038/s41557-024-01443-x)
Supplement: Supplementary file 14 — Excel and ppt files [file 41557_2024_1443_MOESM14_ESM.zip › Fig6_editable.pptx]

## Slide 1
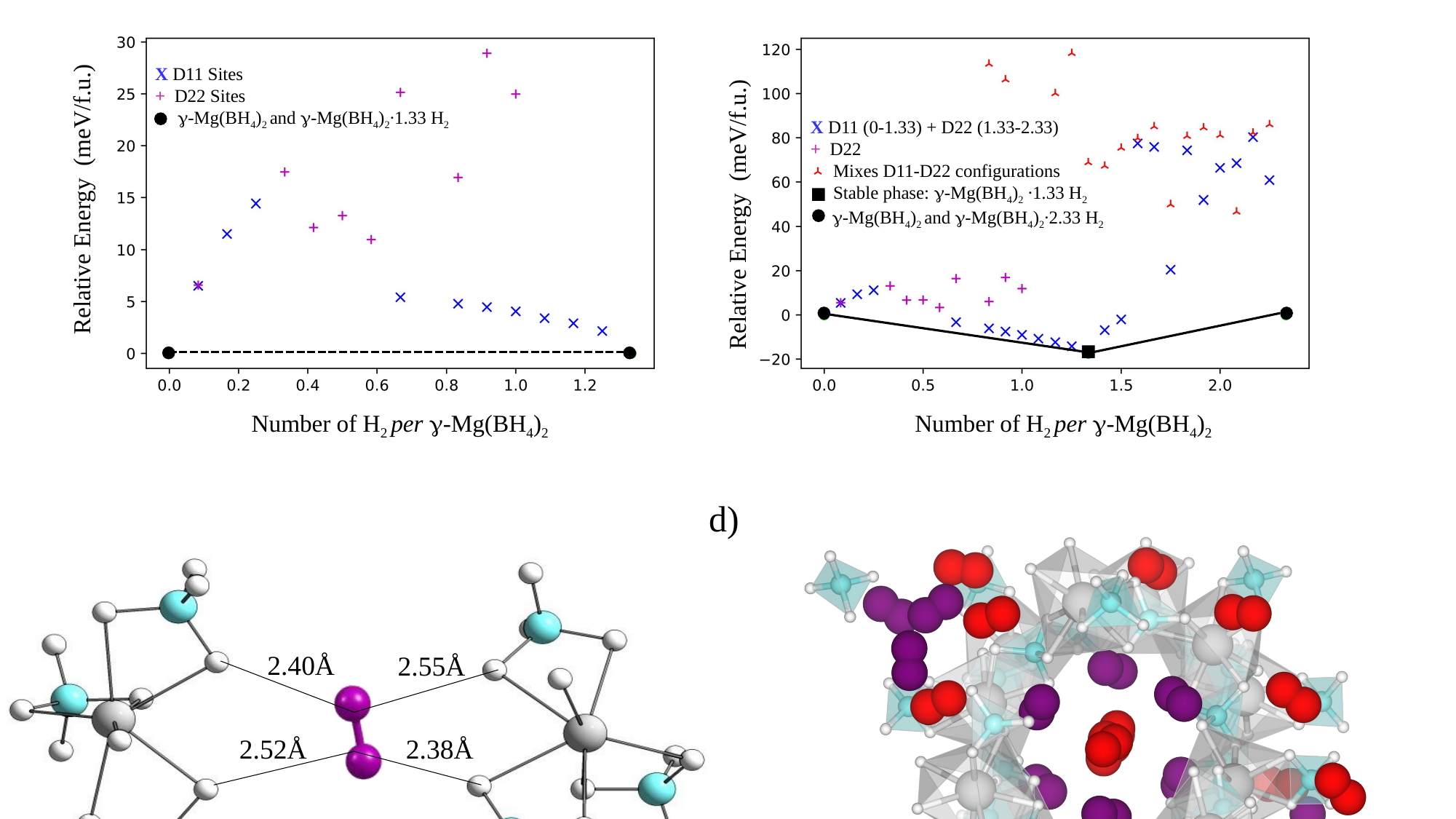

a)
b)
X D11 Sites
+ D22 Sites
 γ-Mg(BH4)2 and γ-Mg(BH4)2∙1.33 H2
X D11 (0-1.33) + D22 (1.33-2.33)
+ D22
 Mixes D11-D22 configurations
 Stable phase: γ-Mg(BH4)2 ∙1.33 H2
 γ-Mg(BH4)2 and γ-Mg(BH4)2∙2.33 H2
Number of H2 per γ-Mg(BH4)2
Number of H2 per γ-Mg(BH4)2
c)
d)
2.40Å
2.55Å
2.52Å
2.38Å
e)
f)
Relative Energy (meV/f.u.)
Relative Energy (meV/f.u.)

## Slide 2
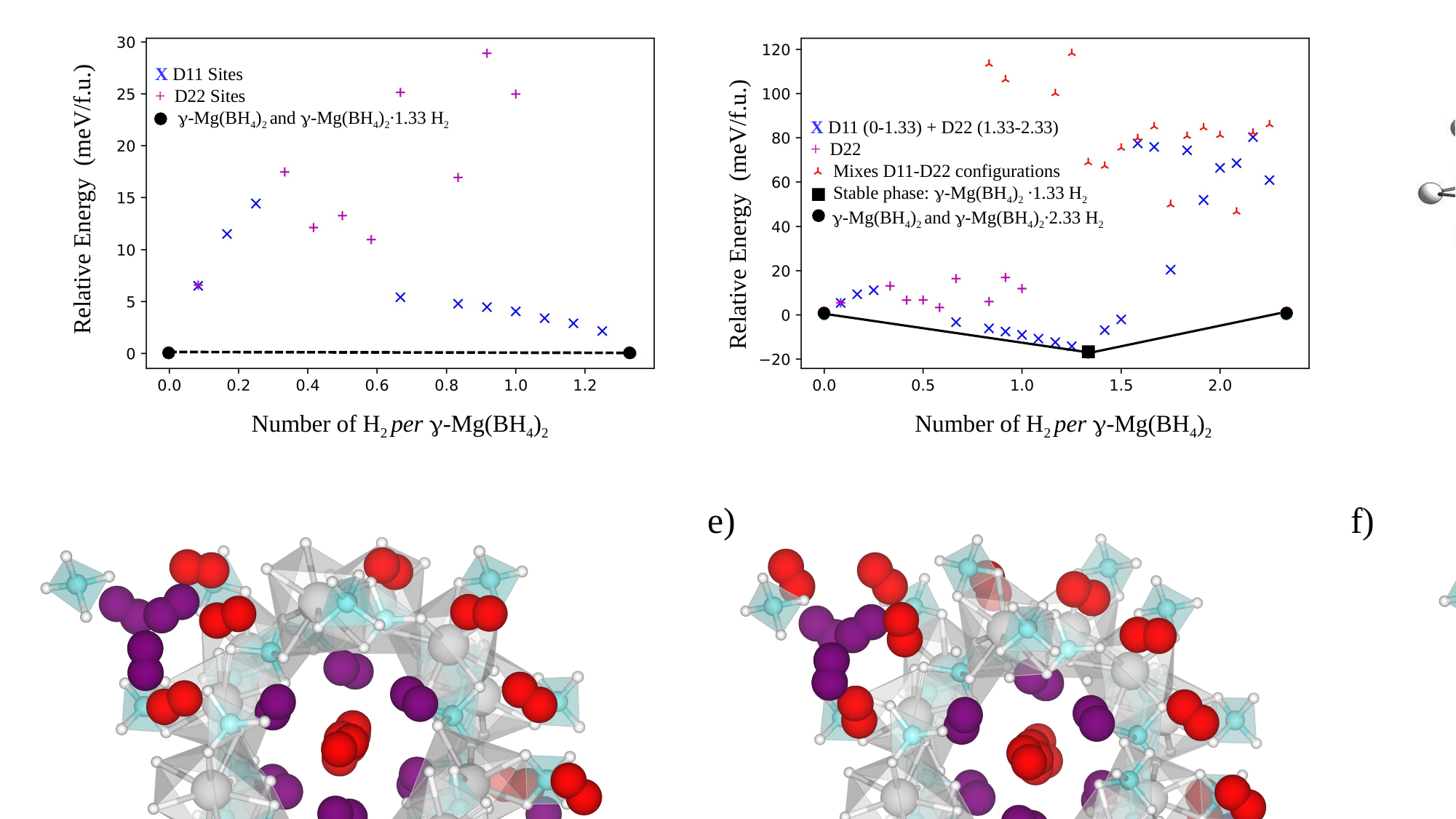

a)
b)
c)
2.40Å
2.55Å
2.52Å
2.38Å
X D11 Sites
+ D22 Sites
 γ-Mg(BH4)2 and γ-Mg(BH4)2∙1.33 H2
X D11 (0-1.33) + D22 (1.33-2.33)
+ D22
 Mixes D11-D22 configurations
 Stable phase: γ-Mg(BH4)2 ∙1.33 H2
 γ-Mg(BH4)2 and γ-Mg(BH4)2∙2.33 H2
Relative Energy (meV/f.u.)
Relative Energy (meV/f.u.)
Number of H2 per γ-Mg(BH4)2
Number of H2 per γ-Mg(BH4)2
d)
e)
f)
